# Multicolor, Cell-Impermeable, and High Affinity BACE1 Inhibitor Probes Enable Superior Endogenous Staining and Imaging of Single Molecules

**DOI:** 10.1021/acs.jmedchem.4c00339

**Published:** 2024-06-06

**Authors:** Florian Stockinger, Pascal Poc, Alexander Möhwald, Sandra Karch, Stephanie Häfner, Christian Alzheimer, Guillaume Sandoz, Tobias Huth, Johannes Broichhagen

**Affiliations:** † Institut für Physiologie und Pathophysiologie, Friedrich-Alexander-Universität Erlangen-Nürnberg, Erlangen 91054, Germany; ‡ Department of Chemical Biology, Max Planck Institute for Medical Research, Heidelberg 69120, Germany; § Leibniz-Forschungsinstitut für Molekulare Pharmakologie, Berlin 13125, Germany; ∥ Université Côte d’Azur, CNRS, INSERM, iBV, Nice 06108, Cedex 2, France; ⊥ Laboratories of Excellence, Ion Channel Science and Therapeutics, Nice 06108, Cedex 2, France

## Abstract

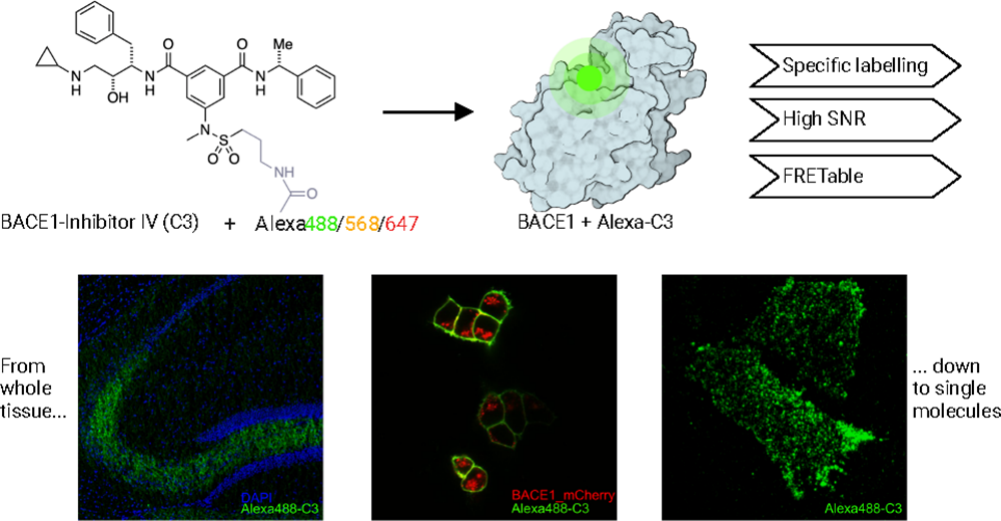

The prevailing but not undisputed amyloid cascade hypothesis places the β-site of APP cleaving enzyme 1 (BACE1) center stage in Alzheimer′s Disease pathogenesis. Here, we investigated functional properties of BACE1 with novel tag- and antibody-free labeling tools, which are conjugates of the BACE1-inhibitor IV (also referred to as C3) linked to different impermeable Alexa Fluor dyes. We show that these fluorescent small molecules bind specifically to BACE1, with a 1:1 labeling stoichiometry at their orthosteric site. This is a crucial property especially for single-molecule and super-resolution microscopy approaches, allowing characterization of the dyes′ labeling capabilities in overexpressing cell systems and in native neuronal tissue. With multiple colors at hand, we evaluated BACE1-multimerization by Förster resonance energy transfer (FRET) acceptor-photobleaching and single-particle imaging of native BACE1. In summary, our novel fluorescent inhibitors, termed **Alexa-C3**, offer unprecedented insights into protein–protein interactions and diffusion behavior of BACE1 down to the single molecule level.

## Introduction

According to the amyloid cascade hypothesis, the proteolytic generation of amyloid β (Aβ) is a central mechanism in Alzheimer’s Disease (AD) pathology.^1,2^ β-Secretase 1 (β-site of APP cleaving enzyme 1, BACE1) is the rate-limiting protease in the generation of Aβ from the amyloid-precursor-protein (APP).^3−6^ Consequently, pharmacological inhibition of BACE1 was advanced as a most promising therapeutic strategy against AD.^7^ So far, however, the outcome of a number of major clinical trials dashed all hopes put in BACE1 inhibitors. The compounds were found to be toxic, had no effect, or, even worse, caused cognitive decline that appeared reversible upon discontinuation of the drug.^8−10^ Presumably, there is a delicate balance of Aβ levels, with deviations in either direction leading to synaptic dysfunction and subsequent cognitive impairment.^11,12^ Despite the failure of BACE1 inhibitors to reverse or at least slow AD progression, the amyloid hypothesis is still supported by a large body of evidence nourishing the hope that BACE1 inhibitors in combination with Aβ antibodies will be effective,^10^ provided that the adverse effects of BACE1 inhibitors are abrogated.^13^

The advent of BACE1 knockout mice was essential to elucidate the many targets and effects of the enzyme apart from Aβ production. Genetic disruption of BACE1 activity in mice engenders a complex phenotype, exhibiting synaptic alterations (reviewed in Das & Yan, 2017),^14^ seizure activity,^15^ sensorimotor and cognitive deficits,^16,17^ metabolic abnormalities (reviewed in^18^), increased neonatal lethality,^19^ and hearing loss.^20^ The molecular underpinnings of the physiological functions of BACE1 are gradually unveiled, as proteins other than APP^21^ are emerging as BACE1 substrates, including neuregulin 1, seizure protein 6, and neural cell adhesion molecule L1-like protein.^10^ In addition, BACE1 was found to affect neuronal excitability through non-proteolytic interactions with ion channel proteins.^22^ On all accounts, a better understanding of the physiological functions of BACE1 is mandatory to further pursue its inhibition in AD therapy. Whereas BACE1 is probably expressed in all central and peripheral neurons as well as in glial cells and many non-neuronal tissues, immunohistochemistry (IHC) of BACE1 is notoriously difficult. In our hands, all antibodies tested so far produced a considerable background in BACE1 knockout mice (e.g., ref (20)). Areas with high expression of the secretase such as the mossy fiber bundle (MFB) in the hippocampus are easily recognized. In contrast, wild-type CA1 neurons and even CA3 neurons, which receive direct mossy fiber input, yield poor signals compared to neurons from BACE1-KO mice.^23^ Undoubtedly, better tools are needed to monitor the dynamic behavior of BACE1, e.g., for investigating trafficking and interaction with other proteins. The ultimate goal here are functional assays to visualize BACE1 in native tissue.

**Figure 1:**
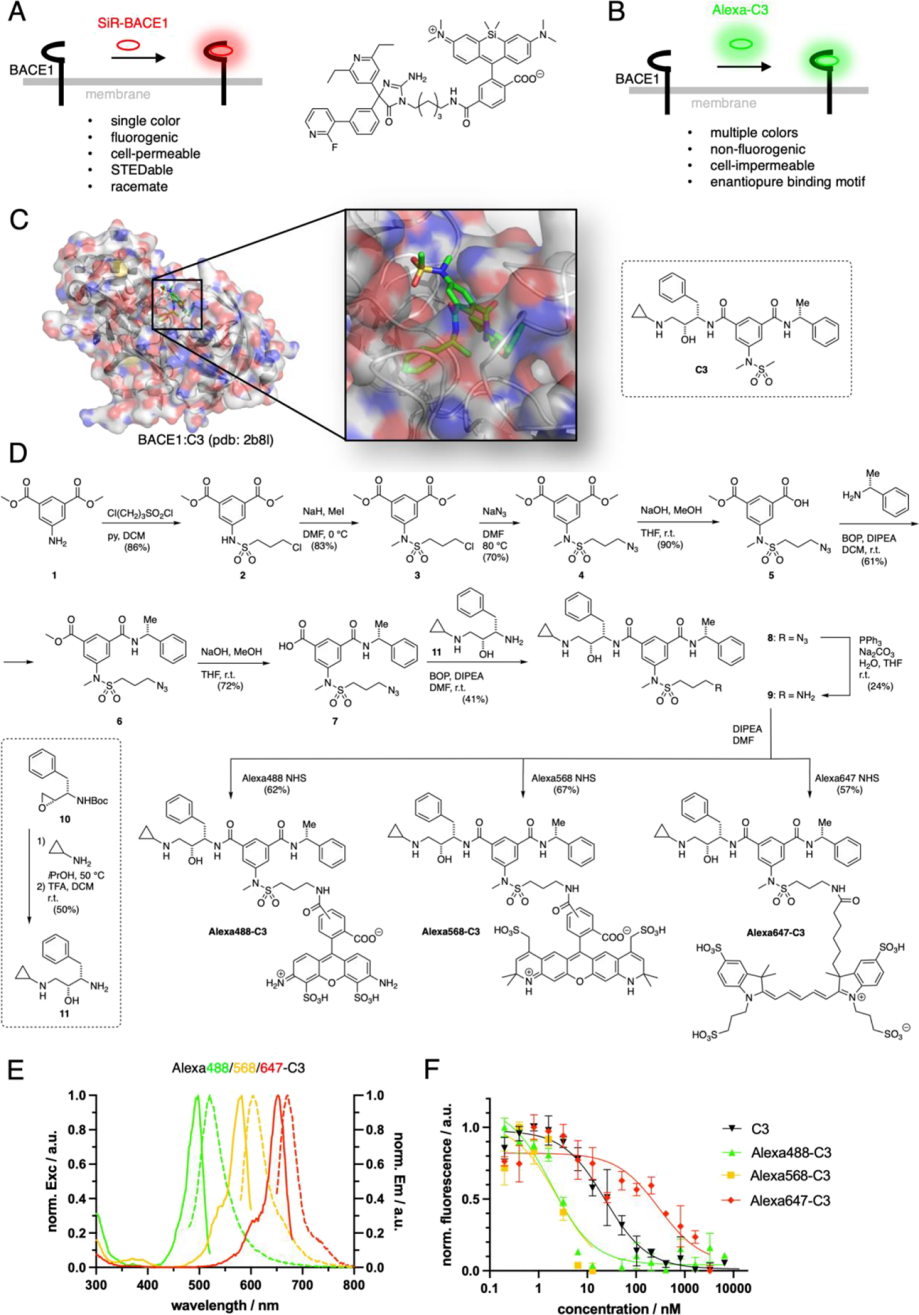
Alexa-C3 conjugates are a novel tool to visualize BACE1 expression. (A) Previously developed small-molecule probe SiR-BACE1 was designed as a conjugate of BACE inhibitor (S)-39^32^ and the far-red dye SiR647^33^ with a linker of four methylene spacers. Due to its fluorogenic properties, fluorescence occurred upon binding to BACE1. (B) New Alexa conjugates are designed as either Alexa Fluor 488, 568, or 647 conjugated to the BACE inhibitor C3 (inhibitor IV),^34^ which is rather hydrophilic compared to (S)-39. (C) Small molecule BACE inhibitor C3 is bound to the catalytic center of BACE1 protease (PDB ID: 2b8l). (D) Multistep chemical scheme depicting the synthesis and late-stage fluorophore functionalization to obtain Alexa-C3 inhibitors. (E) Normalized excitation and emission spectra of the Alexa488-C3, Alexa568-C3, and Alexa647-C3, respectively, were recorded in phosphate-buffered saline solution (PBS). (F) Inhibitory potency of the Alexa-C3 conjugates was assessed using a FRET-based enzymatic assay (see methods). The unconjugated inhibitor C3 was included for reference. The negative logarithm of the half-inhibitory concentration (pIC_50_) was determined using a logistic function. p*IC*_*50*_ were 8.73 (95% CI: 8.58–8.89), 8.61 (95% CI: 8.32–8.90), and 6.53 (95% CI: 6.05–7.05) for Alexa488-C3, Alexa568-C3, and Alexa647-C3, respectively. Because of interference of Alexa568-C3 emission with the emission of the FRET assay, we did not investigate higher concentrations. *n* = 3 for each compound.

Custom-tailored probes that comprise fusion molecules of fluorophores and selective binders to address cell surface proteins are in high demand.^24−30^ In a recent publication,^31^ we made a big step forward by introducing small fluorescent molecules that bind to the catalytic center of BACE1. Our former design was based on the BACE1 inhibitor (S)-39,^32^ which was fused to a far-red silicon rhodamine (SiR647) fluorophore,^33^ to yield fluorogenic SiR-BACE1^31^ (Figure 1A). This construct allowed selective labeling of BACE1 for a number of imaging approaches, including super-resolution stimulated emission depletion (STED) microscopy and single-particle tracking. Unfortunately, partially due to the lipophilic properties of SiR-BACE1, we observed a similar background as in immunostainings.^31^ In addition, SiR-BACE1 accumulated in acidic compartments of living cells, limiting its application in functional assays. In this study, we aimed to overcome the limitations by designing comparable hydrophilic BACE1 inhibitor constructs that are incapable of readily diffusing across cell membranes. This was achieved by fusing sulfonated Alexa dyes to the potent BACE1 inhibitor C3 (also denoted compound IV, Merck).^34^ In contrast to SiR-BACE1, the Alexa congeners are enantiopure, non-fluorogenic, and cell-impermeable in nature (Figure 1B). In addition, we expanded the versatility of the inhibitor constructs for functional assays by having multiple fluorescent colors at our disposal, ensuring compatibility with a broad set of applications.

## Results

### Design and Synthesis of C3 Congeners

Consulting crystal structures of selective BACE1 inhibitors with high affinity lead us to C3 that was introduced for *in vitro* experiments^34^ and became a benchmark inhibitor. Due to its properties, it does not readily cross the blood–brain barrier and was later improved for *in vivo* experiments.^34,35^ C3 forms multiple hydrogen bonds when forming a complex with BACE1, leading to its outstanding efficacy with an IC_50_ in the nanomolar range.^34^ We noticed the solvent’s exposed methyl group residing on the sulfonamide (Figure 1C), which we envisioned to serve as a handle to install a fluorophore. As such, we aimed to prolong this aliphatic residue with a terminal amine group, to late-stage install different Alexa Fluor molecules via their commercially available NHS esters. Indeed, we obtained three different conjugates, after adapting a synthetic sequence from Stachel et al.^34^ (Figure 1D). Commencing with aniline **1** and 3-chloro propanesulfonyl chloride, sulfonamide **2** was obtained, which was further methylated by using sodium hydride and methyl iodide to yield **3**. Chloride **3** was reacted with sodium azide in an S_N_2 reaction to give azide **4**, which was meant to be a masked amine for late-stage installation of the fluorophores. Careful saponification of one methyl ester by stoichiometric amounts of hydroxide gave access to carboxylic acid **5**, which was subsequently amide coupled to (*R*)-1-phenylethan-1-amine using BOP yielding molecule **6**. In a similar sequence, saponification of the remaining methyl ester to free acid **7** and amide coupling to **11** lead to the enantiopure C3 derivative **8** with an azide handle. Amino alcohol **11** was obtained by opening epoxide **10** with propylamine and subsequent deprotection of the Boc-group using TFA. Reducing azide **8** to amine **9** under Staudinger conditions using PPh_3_ in a mixture of THF and water set the stage for fluorophore attachment, which was performed with the NHS esters of AlexaFluor488, 568, and 647, to finally obtain Alexa488-C3, Alexa568-C3, and Alexa647-C3, respectively. The last-stage introduction of the fluorophore has several advantages, i.e., (i) the reduction of more synthetic steps and therefore (ii) the ability to use nanomolar quantities of the costly Alexa-NHS dyes, for which yields could be determined using the available extinction coefficients employing UV/vis measurements and Lambert–Beer’s law.

Assessing the excitation and emission spectra of these compounds showed the expected spectral characteristics of each probe depending on the conjugated dye (Figure 1E). In addition, we performed a fluorescence resonance energy transfer (FRET)-based BACE1 inhibition assay using the recombinant BACE1 extracellular domain and a BACE1 substrate coupled to a donor fluorophore, which is unquenched inversely proportional to the activity of BACE1. All compounds inhibited BACE1 activity with *pIC*_50_s for Alexa488-, Alexa568-, and Alexa647-C3 being 8.73, 8.61, and 6.53, respectively. The original BACE1 inhibitor C3 served as a control and was within the reported range with a *pIC*_50_ = 7.63^34^ (Figure 1F).

### Affinity Studies in Aqueous Solution with Fluorescence Correlation Spectroscopy (FCS)

**Figure 2:**
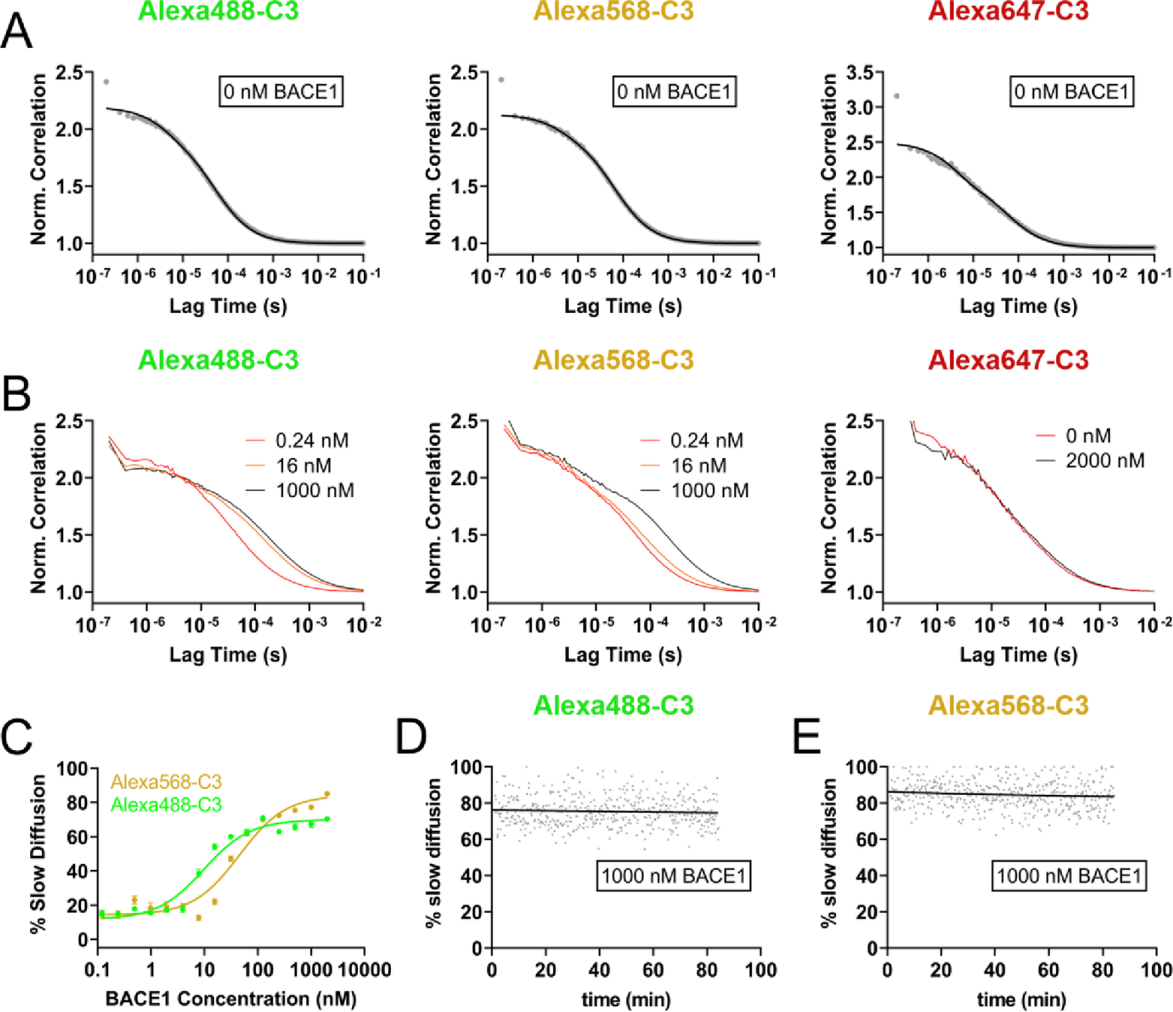
Fluorescence correlation spectroscopy (FCS) confirms high affinity of Alexa-C3 compounds to BACE1. (A, B) 100 nM of free Alexa488-C3, Alexa568-C3 and Alexa647-C3 were recorded using FCS in solution at pH5. (B) Additionally, recombinant BACE1 extracellular domain (ECD) was added with the concentrations as stated in the inset. We fitted the autocorrelated data to a one-component 3D translational model in panel (A). The fast diffusion time constant obtained from the one-component fit (*n* = 3) was used as a constant in the fit of a two-component 3D translational model in panel (B) to obtain the slow diffusion time constant (*n* = 3). (C) Binding of 100 nM Alexa488-C3 and Alexa568 respectively to BACE1 ECD in solution was investigated at different BACE1 concentrations. The amplitude factor (mean of triplicates) of the slow diffusion time constant was plotted against BACE1 concentration. pEC_50_ was then determined with a logistic fit. (D, E) 1000 nM BACE1 ECD and 100 nM Alexa488-C3 or Alexa568-C3 were incubated together and recorded with FCS over a longer period. The slow diffusion time constant determined with the two-component 3D translational model was monitored at intervals of 10 s.

In contrast to the previously developed first-generation fluorescent SiR-BACE1 inhibitor,^31^ the FRET-based BACE1 inhibition assays revealed a higher inhibitory potency of the Alexa-C3 constructs in an aqueous solution. We further characterized the affinity of the constructs to BACE1 using fluorescence correlation spectroscopy (FCS). First, we analyzed free diffusion of 100 nM Alexa-C3 in a HEPES buffered solution (HBS) in the absence of BACE1 (Figure 2A). All FCS experiments were conducted in pH = 5 since this recapitulates the acidic environment of endosomes where BACE1 is mainly located.^3^ For analysis we used an approach described previously.^31^ The autocorrelated FCS data was well fitted with a one-component 3D translational model yielding a single time constant. The diffusion time constants of 100 nM Alexa488-, Alexa568-, and Alexa647-C3 were 35 ± 3, 49 ± 2, and 53 ± 18 μs (mean ± SD), respectively. Next, we determined the diffusion of Alexa-C3 compounds with recombinant BACE1 extracellular domain (ECD) added to the solution (Figure 2B). We fitted each 10 s recording interval to a two-component 3D translational model with one time constant fixed (Figure 2A). As expected, depending on the concentration of BACE1, the second diffusion time constant was higher than that of the freely diffusing Alexa-C3, revealing a slower diffusing population bound to the BACE1 protein. Given the comparably low pIC_50_ for Alexa647-C3 determined by the FRET assay (Figure 1F), it was not surprising that no additional slow component was observed (Figure 2B). The diffusion time constants of the second population were 310 ± 10 and 300 ± 20 μs (mean ± SEM, *n* = 3 preparations) for Alexa488-C3 and Alexa568-C3, respectively. For the determination of the half-maximal effect concentration (pEC_50_), we performed analogous FCS experiments of Alexa488-C3 and Alexa568-C3 in solution with increasing concentrations of BACE1 ECD (Figure 2C). All autocorrelation curves were fitted to a two-component 3D translational model, with both time constants fixed to the diffusion of free and BACE1-bound Alexa-C3 as determined in the preceding experiments. The pEC_50_s determined from the FCS experiments were slightly lower than the pIC_50_s determined with the FRET-assay: 8.01 (95% CI: 7.90–8.13) for Alexa488-C3 and 7.34 (95% CI: 7.21–7.47) for Alexa568-C3. Notably, in contrast to our previous study,^31^ we did not observe an increase of the bound fraction over a longer time period for both Alexa-C3 constructs (Figure 2D,E).

### Determining Affinity and Specificity in a Cellular System

**Figure 3:**
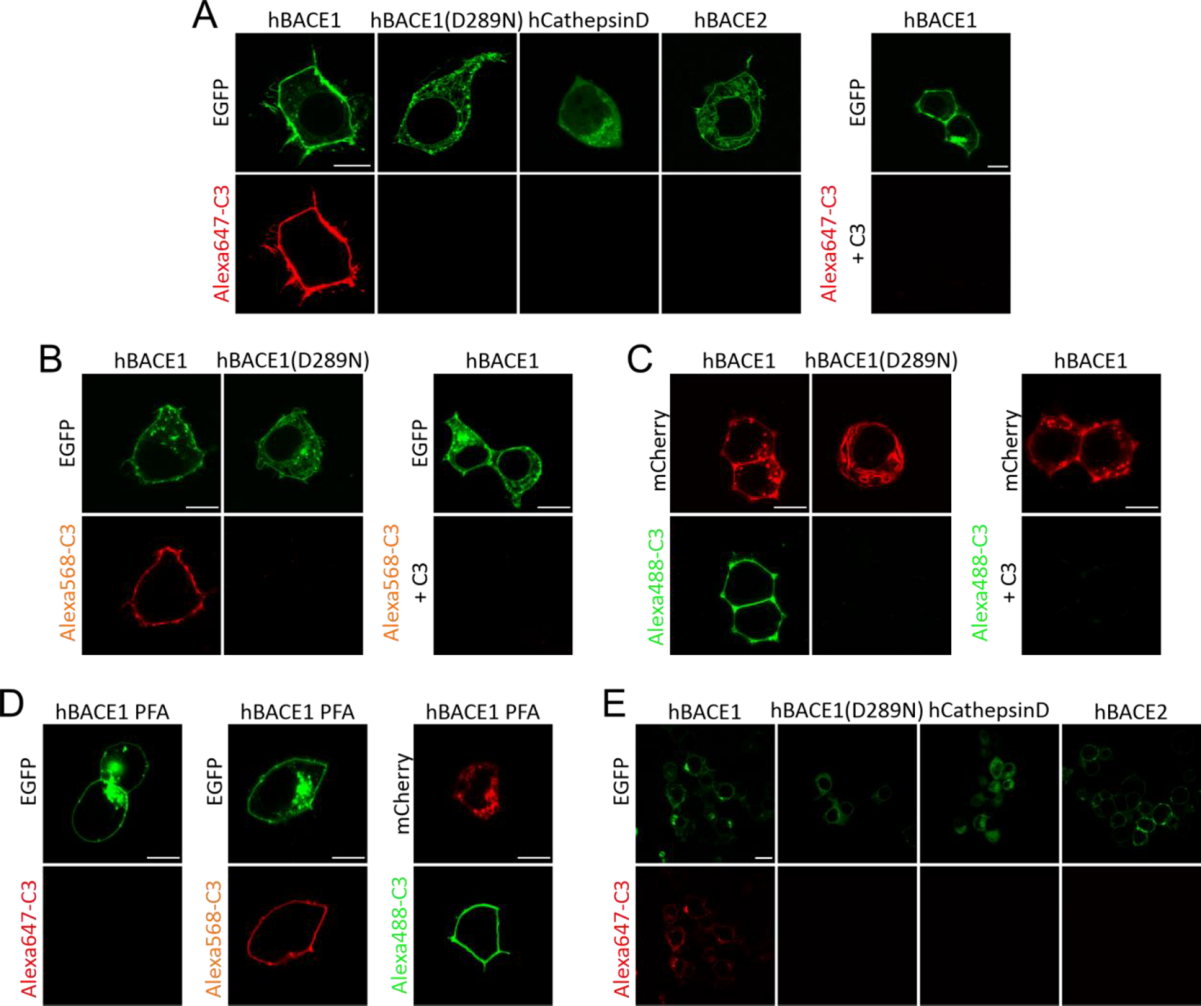
Alexa-C3 compounds bind to BACE1 with high specificity in living HEK293T cells. Cells were transfected with EGFP or mCherry c-terminal fusion constructs of BACE1, BACE2, BACE1 D289N, or cathepsin D. 24 h after transfection living cells were stained with (A, E) 100 nM Alexa647-C3, (B) 10 nM Alexa568-C3, or (C) 50 nM Alexa488-C3. In addition, all compounds were tested after preincubation with 2 μM unconjugated BACE1 inhibitor C3. (D) All conjugates were also probed on cells fixed with 4% paraformaldehyde (PFA) for 15 min prior to staining. (E) To exclude that the protein are not accessible to the Alexa-C3, compounds were permeabilized with 50 μg/mL saponin prior to staining with Alexa647-C3. Scale bars represent 5 μm (A–D) and 20 μm (E). Emission was collected at 493–598 nm (for EGFP), 572–712 nm (for mCherry), 490–568 nm (for Alexa488-C3), 572–712 nm (for Alexa568-C3), and 638–755 nm (for Alexa647-C3). Samples were excited at 488, 561, and 633 nm, respectively.

Next, we explored the performance of the new Alexa-C3 compounds in living cells. Because of the rather hydrophilic properties, we exclusively targeted the cell membrane. To prevent intracellular accumulation, we first performed stainings at 4 °C arresting ATP-dependent endocytosis. In addition to BACE1, the proteolytic inactive BACE1 mutant D289N,^36,37^ the homologue protease BACE2, and another aspartyl protease, Cathepsin D, were transfected in HEK293T cells. All of the constructs were tagged at the C-terminus with either EGFP or mCherry to visualize subcellular expression. Overall, this approach demonstrated the staining of BACE1 with Alexa647-C3 at the plasma membrane without significant background fluorescence (Figure 3A). The other proteins where not labeled by the conjugate indicating high selectivity, which was confirmed by preincubation with 2 μM of unconjugated C3 inhibitor leading to the absence of any staining. We observed similar labeling of BACE1 with Alexa568-C3 (Figure 3B) and Alexa488-C3 (Figure 3C) whereas the inactive BACE1 variant D289N or preincubation with the unconjugated C3 inhibitor gave no appreciable fluorescence. In addition, we performed experiments with membrane permeabilization prior to the staining with Alexa647-C3. With these experiments, we want to exclude that a lack of staining is caused by an impaired accessibility of the protein by Alexa-C3 compounds. Qualitatively, the same specificity was observed in permeabilized cells (Figure 3E). We then tested samples with BACE1 expression after fixation of the cells with paraformaldehyde (PFA). Interestingly, the Alexa647-C3 staining did not yield a considerable signal while we obtained an analogous staining pattern compared to living cells with the other Alexa-C3 conjugates (Figure 3D).

### Visualization of Endogenous BACE1

**Figure 4:**
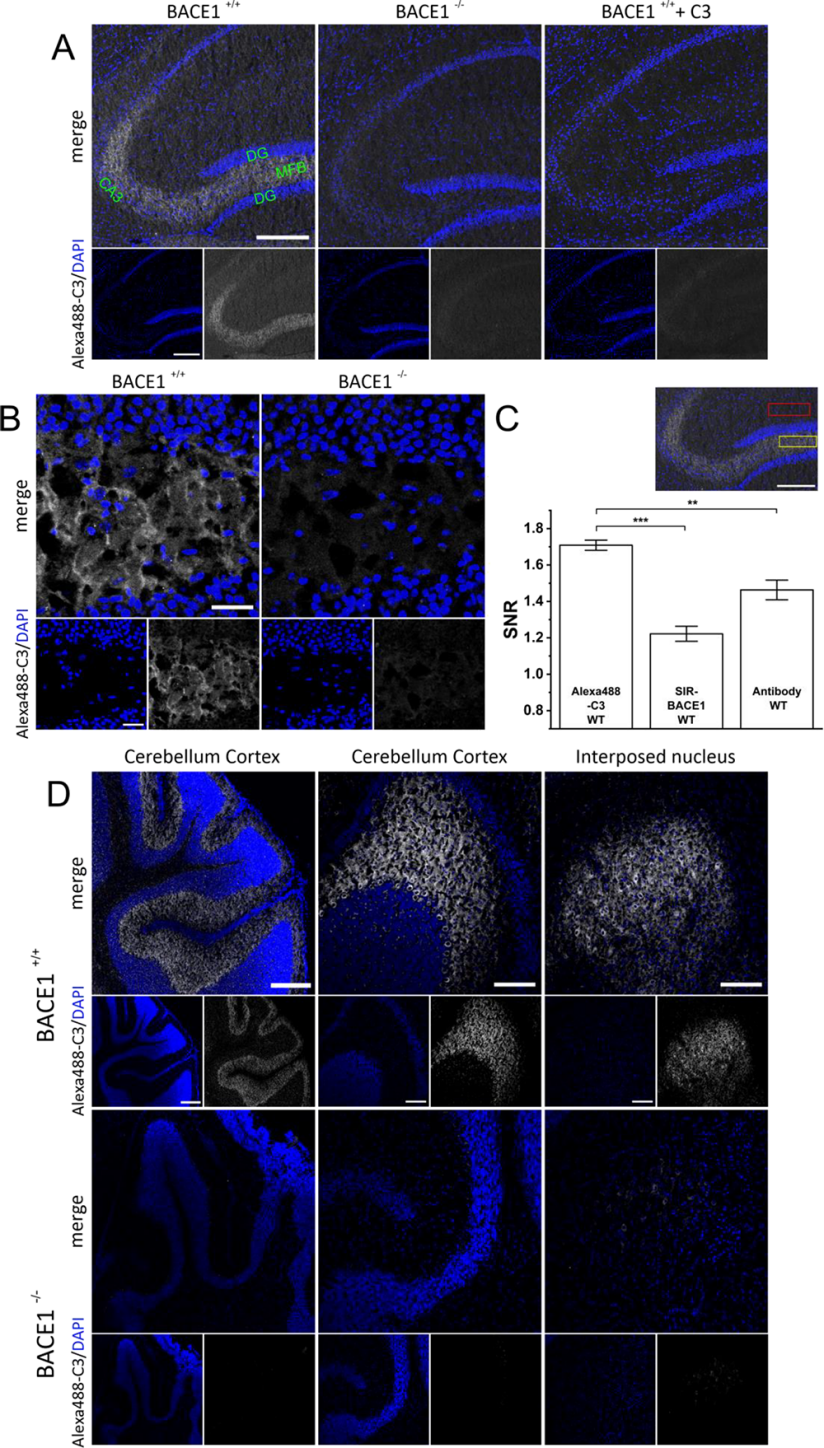
Alexa488-C3 labels endogenous BACE1 in native brain slices of the hippocampus and the cerebellum. Native brain slices from BACE1^+/+^ or BACE1 ^–/–^ mice were incubated with 250 nM Alexa488-C3 conjugate and subject to confocal imaging. (A) Images of the hippocampus depict the mossy fiber bundle (MFB) with high BACE1 expression with the intervening CA3 pyramidal cell layer (CA3) and proximal dentate gyrus (DG). Additionally, slices were preincubated with 2 μM of the unconjugated C3 inhibitor before staining with Alexa488-C3 (right panel) and counterstained with DAPI. The scale bar represents 200 μm. (B) Hilar region of the MFB is displayed with higher magnification. The scale bar represents 25 μm. (C) Ratio of fluorescence intensities of the MFB (yellow ROI) divided by the intensity of the molecular layer of the DG (red rectangle) revealed a high signal to background ratio for Alexa488-C3 compared to the previous construct SIR-BACE1 and a typical antibody staining (rabbit anti-BACE antibody). Alexa488-C3 (*n* = 6 slices), SIR-BACE1 (*n* = 5 slices) (taken from ref (31)), and BACE1 antibody (*n* = 7 slices). The scale bar represents 200 μm. **, *p* < 0.01; ***, *p* < 0.0001. (D) High BACE1 expression of the cerebellar cortex and interposed nucleus is visualized using the Alexa488-C3 conjugate together with DAPI nuclear staining. The scale bar represents 200 μm (left panel) and 100 μm (middle and right panel). Emission was collected at 410–498 nm (for DAPI) and 498–630 nm (for Alexa488-C3). Samples were excited at 405 and 488 nm, respectively.

Staining of endogenous BACE1 proved to be challenging using antibodies. Areas with high expression such as the hippocampal MFB^38^ or cerebellar Purkinje cells^39,40^ are readily distinguished. However, all primary anti-BACE1 antibodies tested in our lab produced a significant background in samples of BACE1 knockout mice.^20,23^ Fluorescent signals in knockout tissue were also observed with our previous SiR647-(S)-39 compound SiR-BACE1.^31^ We wondered if our new Alexa-C3 compounds could outperform existing probes in endogenous tissue. To address this question, we used cryosections of hippocampal and cerebellar brain slices from wild-type (BACE1^+/+^) against knockout (BACE1^–/–^) mice, with wild-type slices incubated with 2 μM of unconjugated C3 inhibitor as a control. In the hippocampus, incubation with 250 nM Alexa488-C3 labeled BACE1 in a typical pattern, including the hilus of the dentate gyrus (DG), the mossy fiber bundle and the CA3 region (Figure 4A). Close to the hilus, we depicted the MFB additionally at higher magnification (Figure 4B). Notably, a certain amount of background fluorescence was present in BACE1^–/–^ tissue (Figure 4A, panel in the middle and Figure 4B, right panel). However, additional incubation of the slices with 2 μM of the unconjugated C3 inhibitor gave a fluorescence signal that was comparable to knockout tissue (Figure 4A, right panel), indicating background fluorescence, which was confirmed by imaging untreated slices. From this set of data and from previous experiments using anti-BACE1 antibodies and SiR-BACE1 we calculated the ratio of the signal in the hilar region to the signal in the molecular layer (Figure 4C), since the molecular layer (red box in Figure 4C) is more affected by background fluorescence due to the relatively low BACE1 expression level. In comparison, Alexa488-C3 yielded a superior ratio.

In addition, we successfully labeled BACE1 in the cerebellum (Figure 4D). The Purkinje cell layer, in particular, showed high BACE1 expression, which is in line with previous results.^39,40^ With higher magnification, individual Purkinje cells displaying high BACE1 expression can be distinguished (Figure 4D, panel in the middle), while the BACE1 staining revealed a high BACE1 expression in the interposed nucleus (Figure 4D, right panel). In comparison, Alexa568-C3 stained hippocampal structures in a similar pattern as Alexa488-C3 with a lower signal–to-background ratio estimated in BACE1^–/–^ tissue (data not shown). Unfortunately, labeling endogenous BACE1 in brain slices with Alexa647-C3 was deemed unsuccessful (data not shown).

### Alexa-C3 Conjugates Allow Investigation of Physical BACE1 Interaction via FRET

**Figure 5:**
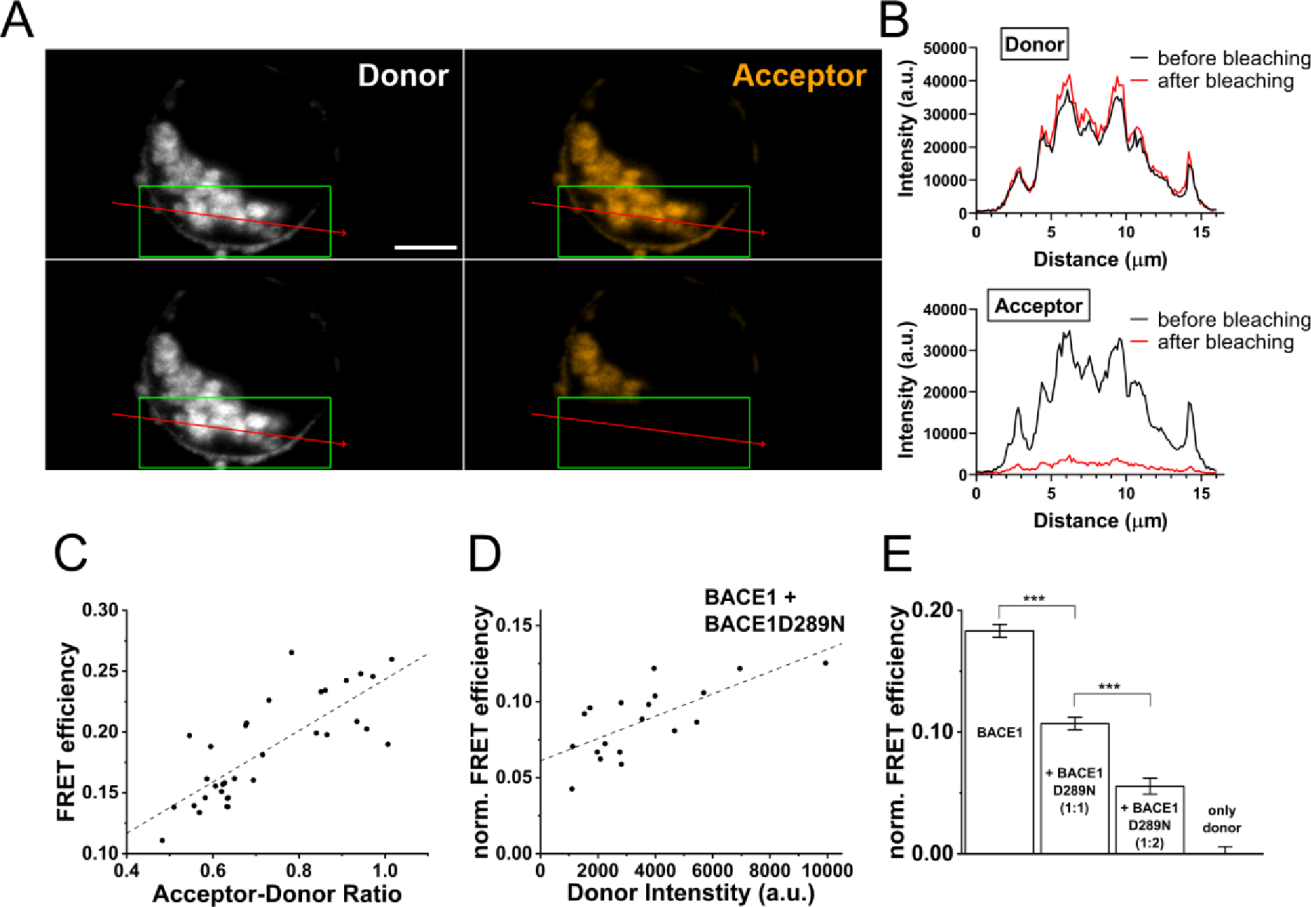
Alexa-C3 conjugates were used to investigate BACE1 multimerization using Förster resonance energy transfer (FRET). HEK293T cells were transfected with BACE1 and inactive BACE1 variant D289N. To access BACE complexes, PFA-fixed cells were incubated with a 1:3 ratio of the FRET pair Alexa488-C3 and Alexa568-C3. (A) Representative images show the fluorescence intensities of the fluorophores before and after photobleaching in the acceptor and donor channel, respectively. The green box denotes the bleaching area. Note the additional accumulation of dye resulting from increased incubation temperature and concentrations than the cellular staining in Figure 3. Scale bar represents 2.5 μm. (B) In addition, fluorescence intensities are displayed along the red arrow in panel (A). Analysis of the fluorescence profile revealed an increasing intensity of Alexa488-C3 (donor) and bleaching of Alexa568-C3 (acceptor). (C) For acceptor-/donor-ratio correction, an additional experiment with constant donor-intensity levels and altering acceptor-intensity levels was performed to obtain the correction factor from the slope of the depicted linear regression. (D) Normalized FRET efficiency with cotransfection of BACE1 and inactive BACE1 D289N in a 1:2 ratio. The FRET efficiency was determined from the constant component of a linear regression. (E) Normalized FRET efficiencies are depicted for transfection of wild-type BACE1 (200 ng), additional transfection of proteolytically inactive BACE1 D289N in a ratio of 1:1 and 1:2 and a control without the acceptor. *n* = 30 (BACE1), *n* = 24 (1:1 ratio), *n* = 20 (1:2 ratio), *n* = 19 (donor only). ***p < 0.0001. Emission was collected at 516–524 nm for Alexa488-C3 and 595–603 nm for Alexa568-C3. Samples were excited at 488 and 561 nm, respectively.

BACE1 is known to dimerize or to form aggregates with higher stoichiometry in overexpressing assays, which was later confirmed *in vivo*.^41,42^ We next asked whether our Alexa-C3 tools would be capable of resolving BACE1 multimerization. Utilizing the availability of different colors, we set up a Förster resonance energy transfer (FRET) acceptor-bleaching assay. In contrast to tag-based approaches, which might lead to altered molecular properties, this method does not require manipulation of BACE1. Alexa488-C3 and Alexa568-C3 should yield a functional FRET pair due to the overlapping emission and excitation spectra (Figure 1E) with a calculated Förster radius of 6.1 nm (according to Wu & Brand, 1994).^43^ To perform the photobleaching experiments, BACE1 was expressed in HEK293T cells and stained in an approximate 1:3 donor-to-acceptor ratio as illustrated in Figure 5A (green box depicts bleaching area). Fluorescent intensities along the red arrow demonstrate the almost complete bleaching of the acceptor and the resulting emission increase of the donor (Figure 5B). It is important to note that the FRET efficiency increases when the donor/acceptor ratio decreases.^44^ To correct for this influencing factor we performed an additional experiment providing a correction factor, estimated from the slope of the linear regression (Figure 5C). The FRET efficiency for different transfections of the wild type and proteolytically inactive BACE1 was estimated from the constant factor of the linear regression as illustrated in (Figure 5D). In these experiments, a considerable FRET efficiency was observed for BACE1 wild type indicating a high degree of multimeric complexes (Figure 5E). As the proteolytically inactive BACE1 variant D289N does not bind Alexa-C3 conjugates (Figure 3B,C), FRET efficiency dropped significantly by incorporating inactive BACE1 into the complexes (Figure 5E). This finding is important since it shows that mutation of the catalytic center does not prevent the formation of multimers. In summary, usage of Alexa488-C3 and Alexa568-C3 as a FRET pair could serve as a valuable tool to investigate BACE1 multimerization.

### Alexa-C3 Conjugates Are Suitable for Single Particle Tracking of Native BACE1

**Figure 6:**
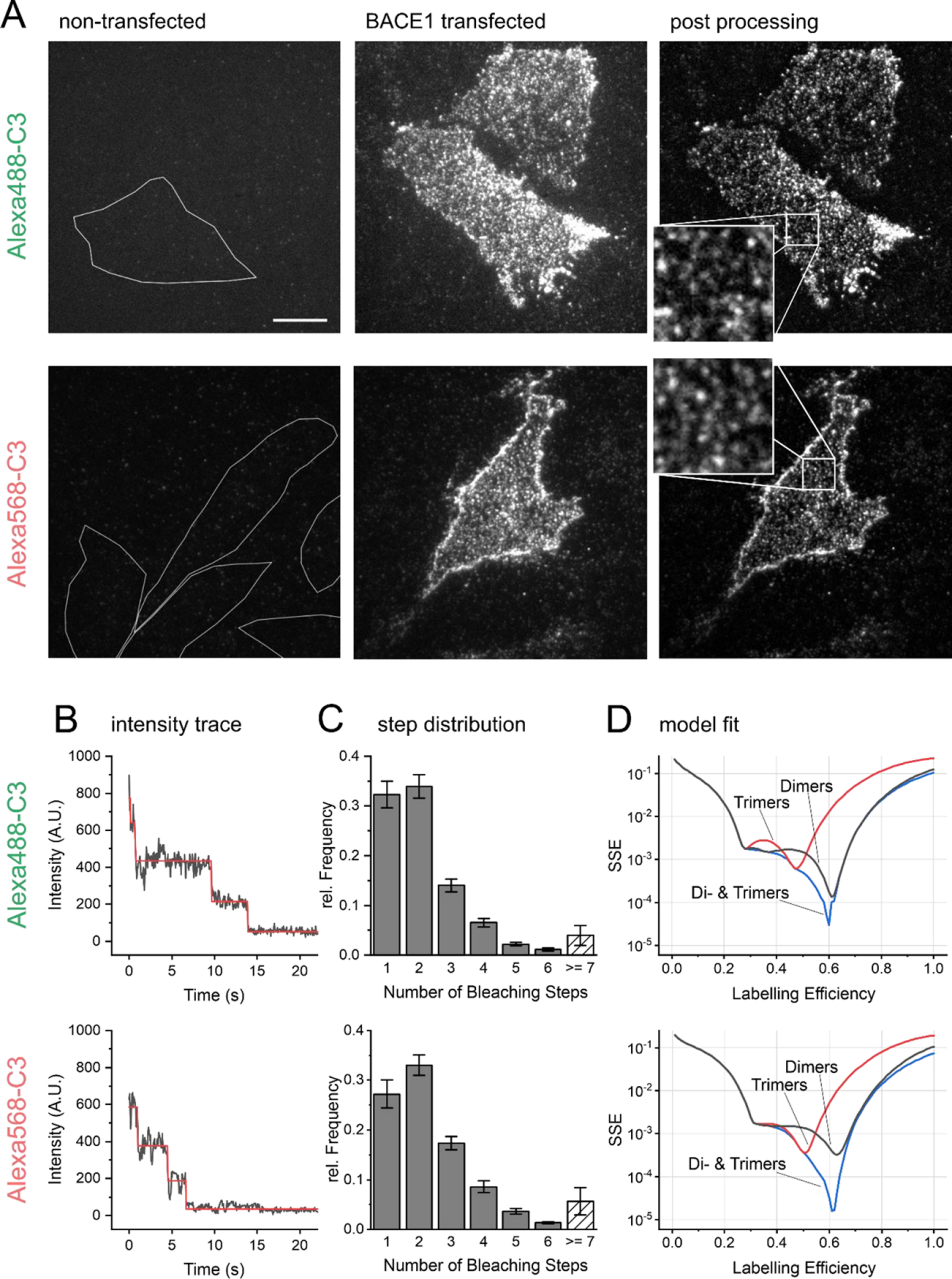
Estimation of Alexa-C3 labeling efficiency using photobleaching step-analysis. In order to obtain fluorescence data on a single molecule level, CHO K1 cells were incubated at 4 °C with 200 nM Alexa488-C3 or 200 nM Alexa568-C3, respectively, fixed using 4% PFA/0.2% glutaraldehyde solution and subsequently captured in TIRF-M (total internal reflection fluorescence microscopy) time lapse recordings. (A) Left panel shows recordings of non-transfected cells, overlaid with cell outlines from corresponding bright field images (not shown). The panel in the middle depicts typical single molecule complexes 48 h after transfection with 50 ng wild type BACE1. The right panel shows the same image after rolling background subtraction using ImageJ to improve the signal-to-background ratio prior to semiautomatic analysis. The insets highlight single fluorescence spots at higher magnification. Scale bar represents 10 μm. (B) Single fluorescence spots were located and fluorescence intensities over time were extracted (see methods). Subsequently, bleaching steps were identified and counted (red: idealized time series). (C) Distributions of step counts proved similar for both Alexa488-C3 (*n* = 15 cells, on average 360 tracks per cell) and Alexa568-C3 (*n* = 12, on average 330 tracks per cell). (D) In order to estimate Alexa-C3 labeling efficiencies, the observed distributions were fitted with a sum of *n*th order binomial distributions for possible BACE1 stoichiometries, calculating the respective residual error: sum of 2nd, 4th, and 6th order for dimers (black line); sum of 3rd and 6th order for trimers (red line); and sum of 2nd, 3rd, 4th, 5th, and 6th order for a combination of dimer and trimers (blue line). Spots with more than six bleaching steps were excluded from analysis.

Due to the high photostability and quantum yield of the Alexa dyes, the C3-compounds are potential tools for single molecule fluorescence microscopy of BACE1. Since there are no tags required, labeling likely does not impair allosteric protein–protein interactions, trafficking or recruitment to microdomains. Because of the small size of the Alexa-C3 conjugates compared to BACE1, the alteration of diffusion within the plasma membrane ought to be minimal. CHO-K1 cells were transfected with wild-type BACE1. After 48 h, the cells were labeled with either 200 nM Alexa488-C3 or 200 nM Alexa568-C3 and subsequently fixed using 4% PFA and 0.2% glutaraldehyde. The samples were then recorded using total internal reflection fluorescence (TIRF) microscopy. With this approach, nontransfected cells did not yield a noticeable fluorescence signal (Figure 6A, left panel). In contrast, BACE1-transfected cells were well distinguishable and the registered fluorescence displayed a signal-to-background ratio suitable for identification of single molecule complexes (Figure 6A, panel in the middle). Prior to analysis, ImageJ rolling background subtraction was applied^45^ (Figure 6A, right panel). With this approach, the signal-to-background ratio was estimated for Alexa488-C3 and Alexa568-C3 to be 4.8 and 5.9, respectively. Single particles were identified and time traces of fluorescence intensity were extracted using GMimPro^46^ (Figure 6B). The time traces were then subjected to a bleaching-step analysis with quickPBSA^47^ (Figure 6B). Counting the number of bleaching steps for each trace, Figure 6C shows the obtained distributions for Alexa488-C3 and Alexa568-C3, respectively. Qualitatively, the distributions are very similar to a recent study that used a monomeric GFP superfolder (msfGFP) tag to label BACE1 protein in-between the propeptide and the protease domain.^41^ The msfGFP was reported previously to display a fluorescence probability of about 50%, defined as combined probability of fluorescence, detection in the specific imaging system and identification in the analysis of a BACE1 molecule.^48^ From the obtained distributions and from further experiments, the authors concluded that BACE1 complexes comprise homotrimers.^41^ Consequently, we fitted our step distributions with a sum of higher-order binomial distributions assuming colocalization of trimers to obtain an estimate for the probability that a BACE1 molecule is labeled and fluorescent. The best fit yielded a probability of roughly 50% for both Alexa-C3 compounds (Figure 6D, red traces). However, assuming the formation of dimers as suggested elsewhere,^42^ the minimum fit error was obtained for a considerable higher fluorescence probability of more than 60% (Figure 6D, dark gray traces). Finally, we assumed composition of the fluorescent spots of a combination of both, BACE1 dimers and trimers. Here, the best fits indicated also a fluorescence probability of about 60%, but with a considerably lower fit error compared to dimers only (Figure 6D, blue traces). Since our step distributions obtained with Alexa-C3 compounds were comparable to the previous study,^41^ it is safe to conclude that the Alexa-compounds exhibit a fluorescence probability of at least 50% in transfected CHO cells.

## Discussion

In this study, we designed and explored small molecule labeling tools to investigate Alzheimer’s protease BACE1. Generation of Aβ from the APP was thought to be central in the pathogenesis of Alzheimer’s disease.^2^ In this proteolytic cascade, the β-secretase BACE1 is the rate-limiting enzyme.^49^ Many efforts have been undertaken to develop and explore BACE1 inhibitors in a clinical setting to alleviate the burden of the disease. However, to our disappointment, all clinical trials with inhibitors have failed.^10^ Both lack of effect and side effects such as additional cognitive decline led to withdrawal of BACE1 inhibitors from clinical settings. Today, there is no clear evidence why BACE1 inhibition was ineffective and the issue has to be resolved before the continuation of clinical trials.^10^ Therefore, a better understanding of BACE1 physiology is mandatory. A refinement of the amyloid hypothesis might be required.^50^ With the development of the new inhibitor constructs, we aim to make a relevant contribution to advance the field. Our new compounds comprise the high affinity BACE1 inhibitor C3^34^ attached to different fluorescent dyes, i.e., Alexa488, Alexa568, and Alexa647.

### Alexa-C3 Conjugates Are Superior to Previously Developed SiR-S39 Inhibitor Constructs

In a previous study, we developed the first generation of BACE1-inhibitor constructs. We introduced a conjugate of the BACE1 inhibitor (S)-39^51^ and SiR647, a fluorogenic silicon rhodamine derivative.^33^ SiR-BACE1 was successfully employed as a tag-free and antibody-free label for BACE1.^31^ It was applicable for confocal, stimulated emission depletion and dynamic single-molecule microscopy. However, two major disadvantages were related to the chemical properties of SiR-BACE1. Prominent unspecific accumulation occurred in primary hippocampal neurons. We related this observation to acidic trapping followed by an on-switch of the fluorogenic SiR647 due to the low pH in lysosomes. Effectively, the silicon rhodamines’ fluorogenicity turned into a disadvantage in this specific setting. Accumulation might be additionally facilitated by BACE1 recycling from the plasma membrane to intracellular vesicles^52^ with the inhibitor construct attached. Consequently, without accumulation, only a small fraction of BACE1 molecules was labeled in plasma membrane lawns (PML).^31^ With a low fluorescence probability of BACE1 molecules, the application in single molecule assays is limited. In the previous study, we found that the affinity was dependent on the linker connecting inhibitor and fluorescence probe. Shorter linkers likely impose steric hindrance for binding to the catalytic center whereas longer linker lengths prevented fluorogenic on-switch of the SiR-dye.^31^ Nevertheless, even with the optimal length of four methylene spacers, the inhibitory potency was substantially lower by at least 100-fold, compared to the parent drug (IC_50_: (S)-39 = 10 nM, SiR647-(S)-39 = 1100 nM).^31^ In this study, attachment of Alexa647 did impair the affinity to the catalytic center by 10-fold, while this was not the case for Alexa488- and Alexa568-C3 conjugates. In fact, the opposite was true for the latter two, as we observed a 10-fold increase of potency of the pIC_50_ in a cell-free assay. We attribute this observation to the nature of the dyes, which are distinct, as Alexa488 and Alexa568 are rhodamine scaffolds and Alexa647 is based on Cyanine5. This vast structural difference is most probably key to these observations, and rhodamine scaffolds have been shown to have interactions with protein surfaces.^53^ We therefore measured excitation and emission spectra of all Alexa-C3 conjugates in PBS alone, in PBS supplemented with BSA, and, to mimic a more complex environment, in suspension with mock Expi293F cells, finding that Alexa568-C3 gives increased fluorescent output in this experiment (+228% and +302%, respectively) (Figure S1), substantiating the possibility of dye–protein interaction. Only subtle changes in elevated fluorescence were observed for Alexa488-C3 (+25%) and Alexa647-C3 (+8%) in the presence of cells. This, and the different number and positioning of sulfonate charges may be the cause for distinct protein surface interactions, influencing the *IC*_*50*_. We also tested if free acids of Alexa488 and Alexa568 (using 0.1 nM concentration due to the sensitivity of the assay) could bind to recombinant BACE1 in the range of 0.3–5000 nM in a nanotemper assay, and did not observe any indication for affinity, Alexa488-C3 served as a positive control with a log*K*_D_ = 6.0, which is in slight contrast to the FCS measurements (Figure 2C), which could be due to the different concentrations and setups used. One might argue that potentially lower IC_50_s come with a trade-off, as Stachel and colleagues^34^ observed with other compounds, that the affinity toward related proteases such as BACE2 and Cathepsin D could increase. At least in live and permeabilized HEK293T cells, we could not detect any relevant staining of overexpressed BACE2, Cathepsin D, and the BACE1 variant D289N with any of our Alexa-C3 compounds. Another important point that we have considered when designing the Alexa compounds, was to increase hydrophilicity to prevent intracellular or intravesicular accumulation. Compared to the (S)-39 inhibitor, which was designed with the aim of a good penetration of the blood-brain barrier,^32^ cell permeation of Alexa-C3 is limited as they display high hydrophilicity compared to SiR647 due to sulfonation. Indeed, this resulted in reduced membrane permeation and vesicular accumulation of the compounds in living cells, especially by staining at 4 °C. Therefore, the lack of fluorogenicity of the Alexa dyes appeared to be no considerable disadvantage. Finally, it is worth mentioning that in contrast to racemic SiR-(S)-39 inhibitors, the binding moiety of all three Alexa-C3 compounds are stemming from an enantiopure precursor, amine **9**, outsourcing the isomer-creating center to the created amide bond between the dye and C3, as Alexa488-NHS and Alexa568-NHS are a mix of 5/6-regioisomers. In summary, compared to the previous SiR-(S)-39 inhibitor constructs, the second-generation Alexa-C3 compounds have a very high affinity to the catalytic center of BACE1 and due to their comparably hydrophilic properties do not easily cross cell membranes.

### High Affinity of Alexa-C3 Compounds to the Catalytic Center of BACE1 Provides Superior Staining Results

Using a commercially available inhibitor assay containing recombinant BACE1 in cell free solution, we determined a pIC_50_ for Alexa488-C3 and Alexa568-C3 of 8.73 and 8.61, respectively. FCS recordings supported these results with the recombinant BACE1 ectodomain yielding slightly less potent pEC_50_ of 8.01 for Alexa488-C3 and 7.34 for Alexa568-C3. The high affinity of the compounds is associated with a superior staining in brain slices, whereas staining of endogenous BACE1 proved to be difficult using antibodies.^54^ Using 250 nM Alexa488-C3, we obtained a better signal-to-background ratio compared to a commonly used commercial antibody (see methods) and the previously published SiR-BACE1. Still, unspecific background signals were evident in BACE1 knockout mice. We can only speculate on whether the residual dye deposition is attributable to off-target binding or to a yet unknown BACE1-related target.

Interestingly, the properties of Alexa647-C3 were distinct from the other two Alexa-C3 compounds. Both, inhibitory potency and affinity were considerably lower. Alexa647-C3 was also not suitable for staining of endogenous BACE1 in neuronal tissue. Still, it was possible to label overexpressed BACE1 with high selectivity over related proteases in living HEK293T cells. Obviously, the high amount of recombinant protein in the experiments is sufficient to obtain a specific signal. As mentioned above, affinity and selectivity are not necessarily correlated.^34^ However, fixating the cells with PFA does completely abolish specific staining, suggesting that PFA cross-linking may mask the catalytic site or further decrease the affinity of the construct to the catalytic center. Other than Alexa488 and Alexa568, Alexa647 lacks nucleophilic groups (primary and secondary amines of Alexa488 and Alexa568, respectively) that may polymerize with PFA. With the design of two high-affinity compounds (Alexa488-C3 and Alexa568-C3), it is conceivable that the C3-BACE1 inhibitor does tolerate the attachment of other chemical moieties without losing its affinity to the catalytic center of BACE1. Thus, the versatility of our approach might be extendible, e.g., by attaching groups for conducting cross-linking experiments.

### Alexa-C3 Compounds Are an Excellent Tool for Studying Protein–Protein interaction

In addition to the numerous proteins cleaved by BACE1,^55−57^ the protease also interacts directly, that is non-proteolytically, with other proteins. The best studied example of such a non-enzymatic and functionally relevant interaction is the modulation by BACE1 of voltage-gated Na^+^ channels^37,58^ and K^+^ channels of the KCNQ family.^36^ In addition, BACE1 is a constituent of protease complexes, interacting with α-secretase,^59^ γ-secretase,^60^ or with itself assembling to homomeric multimers.^41,42,61−63^ Here, we used BACE1 homomerization as a feasible model system to study protein–protein interaction by means of the Alexa-C3 compounds. Indeed, we were able to show BACE1-BACE1 assembly in an assay using Alexa488-C3 and Alexa568-C3 as a FRET pair. Furthermore, we successfully employed both inhibitor constructs to investigate the stoichiometry of single molecule complexes of BACE1. In these assays, application of Alexa-C3 constructs had considerable advantages. No modification of BACE1 is required and since the inhibitor compounds are small molecules, it is reasonable to assume that BACE1 properties related to protein–protein interaction, trafficking, and diffusion are not appreciably altered. Furthermore, the high quantum yield and photostability of the Alexa dyes ensure an outstanding signal-to-noise ratio in single molecule applications. Finally, by using two differently colored constructs in an otherwise identical setting, data can be cross-matched.

Whereas earlier work favored a dimeric assembly of BACE1 (vs), a recent study argued for an exclusively trimeric composition. Importantly we were able to qualitatively reproduce the bleaching step distribution previously reported by Liebsch and colleagues^41^ that was obtained using a monomeric GFP-superfolder tag (msfGFP)-BACE1 fusion protein. In our modeling approach, we got the best approximation of the bleaching step distributions for both Alexa-C3 compounds, if we assumed a mixture of dimeric and trimeric BACE1 complexes. By contrast, model calculations with pure trimeric or mixed BACE1 composition yielded an effective fluorescence probability of around 50% or around 60%, respectively. Given the superior signal-to-noise ratio and the smaller step-size variability using polarized excitation light,^64^ we advance both Alexa-C3 constructs as excellent tools to study single BACE1 molecules.

## Conclusions

We succeeded in creating second-generation multicolor tools for labeling of Alzheimer’s secretase BACE1. Two of the novel Alexa-fused inhibitors retain the high affinity to the catalytic center of BACE1 of the parent small molecule inhibitor C3 (inhibitor IV) and are therefore superior compared to the previously reported SiR-BACE1 constructs. We obtained highly selective, cell impermeant labels, well suited for histochemical stainings of endogenous BACE1. Free of ponderous molecular attachments and endowed with high photostability, the new Alexa-C3 compounds proved very useful in studying protein–protein interactions in a FRET assay and in single-molecule experiments. Thus, the novel compounds are highly promising candidates to track BACE1 in the healthy and diseased brain.

## Experimental Section

### Chemical Synthesis

#### General

All chemical reagents and anhydrous solvents for synthesis were purchased from commercial suppliers (Sigma-Aldrich, Fluka, Acros, Fluorochem, TCI) and were used without further purification or distillation. If necessary, solvents were degassed either by freeze–pump–thaw or by bubbling N_2_ through the vigorously stirred solution for several minutes. For 5,6-regioisomers of Alexa488 and Alexa568 dyes, the 6-regioisomers are shown. All compounds are >95% pure by HPLC analysis.

NMR spectra were recorded in deuterated solvents on a Bruker AVANCE III HD 400 equipped with a CryoProbe and calibrated to residual solvent peaks (^1^H/^13^C in ppm): CDCl_3_ (7.26/77.00), DMSO-*d*_6_ (2.50/39.52), acetone-*d*_6_ (2.05/29.84), MeOD-*d*_4_ (3.31/49.00). Multiplicities are abbreviated as follows: s = singlet, d = doublet, t = triplet, q = quartet, p = pentet, br = broad, m = multiplet. Coupling constants *J* are reported in Hz. Spectra are reported based on appearance, not on theoretical multiplicities derived from structural information.

UPLC-UV/vis for purity assessment was performed on an Agilent 1260 Infinity II LC System equipped with Agilent SB- C18 column (1.8 μm, 2.1 × 50 mm). Buffer A: 0.1% FA in H_2_O Buffer B: 0.1% FA acetonitrile. The typical gradient was from 10% B for 0.5 min → gradient to 95% B over 5 min →95% B for 0.5 min → gradient to 99% B over 1 min with 0.8 mL/min flow. Chromatograms were imported into Graphpad Prism10 and plotted.

High-resolution mass spectrometry was performed using a Bruker maXis II ETD hyphenated with a Shimadzu Nexera system. The instruments were controlled via Brukers otof Control 4.1 and Hystar 4.1 SR2 (4.1.31.1) software. The acquisition rate was set to 3 Hz and the following source parameters were used for positive mode electrospray ionization: End plate offset = 500 V; capillary voltage = 3800 V; nebulizer gas pressure = 45 psi; dry gas flow = 10 L/min; dry temperature = 250 °C. Transfer, quadrupole, and collision cell settings are mass range-dependent and were fine-adjusted with consideration of the respective analyte’s molecular weight. For internal calibration sodium format clusters were used. Samples were desalted via fast liquid chromatography. A Supelco Titan C18 UHPLC Column, 1.9 μm, 80 Å pore size, 20 × 2.1 mm and a 2 min gradient from 10 to 98% aqueous MeCN with 0.1% FA (H_2_O: Carl Roth GmbH + Co. KG ROTISOLV Ultra LC-MS; MeCN: Merck KGaA LiChrosolv Acetonitrile hypergrade for LC-MS; FA - Merck KGaA LiChropur Formic acid 98%–100% for LC-MS) was used for separation. Sample dilution in 10% aqueous ACN (hyper grade) and injection volumes were chosen dependent on the analyte’s ionization efficiency. Hence, on-column loadings were between 0.25 and 5.0 ng. Automated internal recalibration and data analysis of the recorded spectra were performed with Bruker’s DataAnalysis 4.4 SR1 software.

Preparative RP-HPLC was performed on a Waters e2695 system equipped with a 2998 PDA detector for product collection (at 220, 490, 550, or 650 nm) on either a semipreparative Supelco Ascentis C18 HPLC Column (5 μm, 250 × 21.2 mm) or on an analytical Supelco Ascentis C18 HPLC Column (3 μm, 150 × 2.1 mm). Buffer A: 0.1% TFA in H_2_O Buffer B: MeCN. The typical gradient for semipreparative was from 10% B for 5 min → gradient to 90% B over 45 min → 90% B for 5 min → gradient to 99% B over 5 min with 8 mL/min flow. The typical gradient for analytical was from 10% B for 5 min → gradient to 90% B over 30 min → 90% B for 5 min → gradient to 99% B over 5 min with 4 mL/min flow.

Flash column chromatography (FCC) was performed on a Biotage Isolera One with prepacked silica columns (0.040–0.063 mm, 230–400 mesh, Silicycle). Reactions and chromatography fractions were monitored by thin layer chromatography (TLC) on Merck silica gel 60 F254 glass plates. The spots were visualized under UV light at 254 nm.

#### Dimethyl-5-((3-chloropropyl)sulfonamido)isophthalate (**2**)

A round-bottom flask was charged with dimethyl 5-aminoisophthalate (**1**) (5.00 g, 23.9 mmol, 1.0 equiv), 75 mL of DCM, and 25 mL of pyridine. 3-Chloropropane-1-sulfonyl chloride (4.23 g, 23.9 mmol, 1.0 equiv) was added dropwise to the suspension under vigorous stirring, and the reaction mixture was stirred for additional 4 h at r.t. while turning red. The mixture was quenched by addition of 200 mL aqueous HCl (1 M) and extracted with DCM (2 × 200 mL). The combined organic layers were washed with 200 mL aqueous HCl (1 M) and brine, filtered over MgSO_4_ and dried to obtain 6.94 g (20.6 mmol) of the desired product as a red powder in 86% yield.

##### ^1^H NMR (400 MHz, acetone-*d*_6_)

δ [ppm] = 8.35–8.30 (m, 1H), 8.22 (d, *J* = 1.5 Hz, 2H), 3.93 (s, 6H), 3.74 (t, *J* = 6.5 Hz, 2H), 3.51–3.30 (m, 2H), 2.36–2.22 (m, 2H).

##### ^13^C NMR (101 MHz, acetone-*d*_6_)

δ [ppm] = 166.0, 140.1, 132.8, 126.2, 125.0, 52.9, 49.7, 43.5, 27.8.

##### HRMS (ESI)

Calc. for C_13_H_17_ClNO_6_S [M + H]^+^: 350.0460 and 352.0430, found: 350.0460 and 352.0431.

#### Dimethyl-5-((3-chloro-*N*-methylpropyl)sulfonamido)isophthalate (**3**)

A flame-dried round-bottom Schlenk flask was charged with 6.44 g (18.4 mmol, 1.0 equiv) of **2** and dissolved in 100 mL DMF under a nitrogen atmosphere and cooled to 0 °C. MeI (2.39 mL, 5.44 g, 38.4 mmol, 2.1 equiv) was added dropwise under vigorous stirring, before 920 mg (23.0 mmol, 1.25 equiv) of NaH (60% in mineral oil) was added portionwise. The reaction mixture turned dark purple and was allowed to warm to r.t. under stirring over 3 h. 200 mL of EtOAc was added and was washed with dH_2_O (2 × 250 mL) and brine, filtered over MgSO_4_, and dried to obtain 5.56 g (15.3 mmol) of the desired product as a yellow oil that solidified upon standing in 83% yield.

##### ^1^H NMR (400 MHz, CDCl_3_)

δ [ppm] = 8.58 (t, *J* = 1.5 Hz, 1H), 8.22 (d, *J* = 1.5 Hz, 2H), 3.95 (s, 6H), 3.64 (t, *J* = 6.1 Hz, 2H), 3.40 (s, 3H), 3.26–3.13 (m, 2H), 2.33–2.24 (m, 2H).

##### ^13^C NMR (101 MHz, CDCl_3_)

δ [ppm] = 165.3, 141.9, 131.9, 131.0, 129.2, 52.6, 47.0, 42.7, 38.2, 26.3.

#### HRMS (ESI)

Calc. for C_14_H_19_ClNO_6_S [M + H]^+^: 364.0616 and 366.0587, found: 364.0613 and 366.0585.

#### Dimethyl-5-((3-azido-*N*-methylpropyl)sulfonamido)isophthalate (**4**)

A flame-dried round-bottom Schlenk flask was charged with 356 mg (0.99 mmol, 1.0 equiv) of **3** and dissolved in 30 mL of DMF under a nitrogen atmosphere. NaN_3_ (74.0 mg, 1.14 mmol, 1.15 equiv) was added and the reaction mixture was heated to 80 °C under stirring for 3 h, before dH_2_O (500 mL) was added and the desired product was sedimented by centrifugation (4,000 rpm for 60 min), the supernatant was collected, and the residue was dried to obtain the desired product as a yellow oil that solidifies upon standing. The aqueous layer was re-extracted with 600 mL of EtOAc, which was dried over MgSO_4_ before all volatiles were removed and dH_2_O (200 mL) was added and more product was sedimented by centrifugation (4,000 rpm for 60 min) to obtain a total of 258 mg (0.70 mmol) of the desired product as an orange solid in 70% yield.

##### ^1^H NMR (400 MHz, CDCl_3_)

δ [ppm] = 8.60 (t, *J* = 1.5 Hz, 1H), 8.22 (d, *J* = 1.5 Hz, 2H), 3.95 (s, 6H), 3.47 (t, *J* = 6.4 Hz, 2H), 3.19–2.97 (m, 2H), 2.10–2.03 (m, 2H).

##### ^13^C NMR (101 MHz, CDCl_3_)

δ [ppm] = 165.3, 141.9, 131.9, 131.0, 129.2, 52.7, 49.5, 46.7, 38.2, 23.1.

##### HRMS (ESI)

Calc. for C_14_H_19_N_4_O_6_S [M + H]^+^: 371.1020, found: 371.1020.

#### 3-((3-Azido-*N*-methylpropyl)sulfonamido)-5-(methoxycarbonyl)benzoic acid (**5**)

A round-bottom flask was charged with 173 mg (467 μmol, 1.0 equiv) of **4** and dissolved in 5 mL of THF, 5 mL of MeOH, and 240 μL of aqueous NaOH (2 M). The reaction mixture was stirred overnight at r.t. before all volatiles were removed *in vacuo* and to obtain 159 mg (420 μmol, Na-salt) of the desired product sufficiently pure as a white foam in 90% yield.

##### ^1^H NMR (400 MHz, MeOD-*d*_4_)

δ [ppm] = 8.53 (t, *J* = 1.5 Hz, 1H), 8.20 (dd, *J* = 2.3, 1.5 Hz, 1H), 8.10 (dd, *J* = 2.3, 1.6 Hz, 1H), 3.93 (s, 3H), 3.48–3.42 (m, 2H), 3.39 (s, 3H), 3.25–3.17 (m, 2H), 2.11–1.88 (m, 2H).

##### ^13^C NMR (101 MHz, MeOD-*d*_4_)

δ [ppm] = 172.8, 167.7, 142.9, 141.3, 132.0, 131.8, 130.4, 129.9, 52.8, 50.7, 47.5, 38.6, 24.3.

##### HRMS (ESI)

Calc. for C_13_H_17_N_4_O_6_S [M + H]^+^: 357.0863, found: 357.0862.

#### Methyl-(*R*)-3-((3-azido-*N*-methylpropyl)sulfonamido)-5-((1-phenylethyl)-carbamoyl)benzoate (**6**)

A round-bottom flask was charged with 159 mg (420 μmol, 1.0 equiv) of **5** (Na-salt), 61 mg (40 μL, 504 μmol, 1.2 equiv) of (*R*)-1-phenylethan-1-amine, and 223 mg (504 μmol, 1.2 equiv) of BOP dissolved in 5 mL DCM and 220 μL (1.26 mmol, 3.0 equiv) of DIPEA. The reaction mixture was stirred for 2 h at r.t. before it was filtered and directly subjected to FCC (DCM/MeOH, gradient from 100/0 → 90/10 over 15 CV) to obtain 118 mg (257 μmol) of the desired product as a clear oil in 61% yield.

##### ^1^H NMR (400 MHz, CDCl_3_)

δ [ppm] = 8.20 (dt, *J* = 3.4, 1.5 Hz, 1H), 8.09 (dd, *J* = 2.3, 1.4 Hz, 1H), 8.07–7.96 (m, 1H), 7.40–7.35 (m, 2H), 7.35–7.29 (m, 2H), 7.26–7.21 (m, 1H), 6.96 (d, *J* = 7.7 Hz, 1H), 5.28 (m, 1H), 3.86 (s, 3H), 3.42 (t, *J* = 6.5 Hz, 2H), 3.32 (s, 3H), 3.14–3.05 (m, 2H), 2.19–1.88 (m, 2H), 1.59 (d, *J* = 6.9 Hz, 3H).

##### ^13^C NMR (101 MHz, CDCl_3_)

δ [ppm] = 165.4, 164.3, 142.8, 141.9, 135.9, 131.4, 129.9, 129.1, 128.6, 127.4, 126.2, 125.9, 52.5, 49.6, 49.3, 46.5, 38.0, 22.9, 21.5.

##### HRMS (ESI)

Calc. for C_21_H_26_N_5_O_5_S [M + H]^+^: 460.1649, found: 460.1649.

#### (*R*)-3-((3-Azido-*N*-methylpropyl)sulfonamido)-5-((1-phenylethyl)carbamoyl)-benzoic acid (**7**)

A round-bottom flask was charged with 118 mg (257 μmol, 1.0 equiv) of **6** and dissolved in 2 mL of THF, 2 mL of MeOH, and 130 μL of aqueous NaOH (2 M). The reaction mixture was stirred overnight at r.t. before 20 μL of glacial HOAc was added and all volatiles were removed *in vacuo*. The residue was dissolved in DMF:dH_2_O (9:1) and subjected to RP-HPLC (MeCN:H_2_O + 0.1% TFA, gradient 10:90 → 90:10 over 60 min, flow 8 mL/min, λ = 220 nm) to obtain 83 mg (186 μmol) of the desired product after lyophilization as a white powder in 72% yield.

##### ^1^H NMR (400 MHz, DMSO-*d*_6_)

δ [ppm] = 9.12 (d, *J* = 7.8 Hz, 1H), 8.44–8.37 (m, 1H), 8.15–8.12 (m, 1H), 8.12–8.06 (m, 1H), 7.46–7.38 (m, 2H), 7.33 (t, *J* = 7.6 Hz, 2H), 7.23 (td, *J* = 7.0, 1.4 Hz, 1H), 5.20 (p, *J* = 7.2 Hz, 1H), 3.44 (t, *J* = 6.7 Hz, 2H), 3.35 (s, 3H), 3.29–3.22 (m, 2H), 1.88 (dq, *J* = 9.9, 6.9 Hz, 2H), 1.50 (d, *J* = 7.1 Hz, 3H).

##### ^13^C NMR (101 MHz, DMSO-*d*_6_)

δ [ppm] = 166.3, 163.9, 144.6, 141.8, 135.8, 131.9, 129.2, 129.1, 128.3, 126.7, 126.3, 126.1, 48.9, 48.7, 46.0, 37.7, 22.7, 22.0.

##### HRMS (ESI)

Calc. for C_20_H_23_N_5_O_5_S [M + H]^+^: 446.1493, found: 446.1493.

#### (2*R*,3*S*)-3-Amino-1-(cyclopropylamino)-4-phenylbutan-2-ol (**11**)

**11** was prepared according to a literature procedure^34^ with slight modifications: in a round-bottom flask, 1.2 mL (17.23 mmol, 8.6 equiv) of cyclopropyl amine were added to a solution of 526 mg (1.99 mmol, 1.0 equiv) (2*S*,3*S*)-1,2-epoxy-3-(Boc-amino)-4-phenylbutane (**10**) in 6 mL of *i*PrOH. The white suspension was stirred at 50 °C for 16 h. The reaction mixture was evaporated to dryness to afford a white solid. The solid was dissolved in 4 mL of DCM and 1 mL of neat TFA and stirred for 1 h. The solvent was evaporated, and the resulting oil was loaded on 15 g silica gel, washed with 40 mL of 5% MeOH:DCM, and eluted with 30% MeOH:DCM (50 mL). The solvent was evaporated to afford a yellow oil. The resulting oil was dissolved in 15 mL of dH_2_O:MeCN (9:1) and freeze-dried to obtain 670 mg of a crude product (double TFA salt: 1.0 mmol, 50%) as a yellowish oil, which was used without further purification.

##### HRMS (ESI)

Calc. for C_13_H_21_N_2_O [M + H]^+^: 221.1648, found: 221.1648.

#### 5-((3-Azido-*N*-methylpropyl)sulfonamido)-*N*^1^-((2*S*,3*R*)-4-(cyclopropylamino)-3-hydroxy-1-phenylbutan-2-yl)-*N*^3^-((*R*)-1-phenylethyl)isophthalamide (**8**)

A round-bottom flask was charged with 74.0 mg (164 μmol, 1.2 equiv.; calculated as double TFA salt) of **11** and 61.0 mg (137 mmol, 1.0 equiv) of **7** dissolved in 2 mL of DMF and 100 μL (74 mg, 573 mmol, 4.2 equiv) of DIPEA, to which 73.0 mg (164 μmol, 1.2 equiv) of BOP was added in one portion and the reaction mixture was stirred at r.t. for 4 h. 100 μL of HOAc and 200 μL of water were added, and the mixture was subjected to RP-HPLC (MeCN:H_2_O + 0.1% TFA, gradient 10:90 → 90:10 over 60 min, flow 8 mL/min, λ = 220 nm) to obtain 36.0 mg (55.6 μmol) of the desired product as a white powder after lyophilization in 41% yield.

##### ^1^H NMR (400 MHz, DMSO-*d*_6_)

δ [ppm] = 9.00 (d, *J* = 7.9 Hz, 1H), 8.73 (br s, 1H), 8.68–8.59 (m, 1H), 8.54 (d, *J* = 8.8 Hz, 1H), 8.27–8.11 (m, 1H), 8.09–7.97 (m, 1H), 7.93–7.83 (m, 1H), 7.43–7.37 (m, 2H), 7.37–7.30 (m, 2H), 7.29–7.18 (m, 5H), 7.17–7.09 (m, 1H), 5.91 (br s, 1H), 5.18 (p, *J* = 7.1 Hz, 1H), 4.28–4.09 (m, 1H), 3.98–3.82 (m, 1H), 3.47 (t, *J* = 6.7 Hz, 2H), 3.33 (s, 3H), 3.30–3.21 (m, 2H), 3.21–3.09 (m, 1H), 3.09–2.93 (m, 1H), 2.83 (dd, *J* = 13.8, 10.8 Hz, 1H), 2.78–2.66 (m, 1H), 2.03–1.82 (m, 2H), 1.50 (d, *J* = 7.1 Hz, 3H), 1.03–0.68 (m, 4H).

##### ^13^C NMR (101 MHz, DMSO-*d*_6_)

δ [ppm] = 165.7, 164.8, 145.0, 141.9, 139.3, 136.1, 135.6, 129.6, 128.7, 128.6, 128.2, 128.0, 127.2, 126.6, 126.5, 125.6, 69.1, 55.0, 51.4, 49.4, 49.1, 46.5, 38.3, 35.7, 30.4, 23.1, 22.5, 3.8, 3.5 (two distinct ^13^C signals from cyclopropyl CH_2_–groups confirmed by HSQC).

##### HRMS (ESI)

Calc. for C_33_H_42_N_7_O_5_S [M + H]^+^: 648.2963, found: 648.2958.

#### 5-((3-Amino-*N*-methylpropyl)sulfonamido)-*N*^1^-((2*S*,3*R*)-4-(cyclopropylamino)-3-hydroxy-1-phenylbutan-2-yl)-*N*^3^-((*R*)-1-phenylethyl)isophthalamide (**9**)

A round-bottom flask was charged with 16.0 mg (24.7 μmol, 1.0 equiv) of **8** dissolved in 4 mL of THF before 36.0 mg (136 μmol, 5.5 equiv) of triphenylphosphine was added in one portion and the solution was stirred at r.t. for 16 h. The solvent was evaporated to afford a white solid that was redissolved in 500 and 500 μL aqueous saturated Na_2_CO_3_ to obtain a suspension that was stirred for 1 h at r.t. before all volatiles were evaporated and the white residue was taken up in 500 μL of DMF and 300 μL of water and subjected to RP-HPLC (MeCN:H_2_O + 0.1% TFA, gradient 10:90 → 90:10 over 60 min, flow 8 mL/min, λ = 220 nm) to obtain 3.7 mg (5.9 μmol) of the desired product after lyophilization as a white powder in 24% yield.

##### ^1^H NMR (400 MHz, DMSO-*d*_6_)

δ [ppm] = 9.01 (d, *J* = 8.0 Hz, 1H), 8.71 (br s, 1H), 8.62 (br s, 2H), 8.55 (d, *J* = 8.9 Hz, 1H), 8.19 (d, *J* = 1.6 Hz, 1H), 7.99 (t, *J* = 1.8 Hz, 1H), 7.88 (t, *J* = 1.8 Hz, 1H), 7.75 (br s, 3H), 7.43–7.37 (m, 2H), 7.33 (dd, *J* = 8.5, 6.7 Hz, 2H), 7.28–7.20 (m, 5H), 7.18–7.08 (m, 1H), 5.90 (s, 1H), 5.18 (p, *J* = 7.2 Hz, 1H), 4.34–4.08 (m, 1H), 3.88 (t, *J* = 8.8 Hz, 1H), 3.32 (s, 3H), 3.30 (m, 2H), 3.14 (dd, *J* = 14.0, 3.3 Hz, 1H), 2.95–2.87 (m, 2H), 2.83 (dd, *J* = 13.9, 10.8 Hz, 1H), 2.73 (t, *J* = 5.3 Hz, 1H), 1.97 (p, *J* = 7.7 Hz, 2H), 1.50 (d, *J* = 7.0 Hz, 3H), 1.05–0.60 (m, 4H).

##### ^13^C NMR (101 MHz, DMSO-*d*_6_)

δ [ppm] = 165.2, 164.4, 144.6, 141.3, 138.9, 135.7, 135.2, 129.1, 128.3, 128.1, 128.0, 127.4, 126.8, 126.1, 126.0, 125.1, 68.6, 54.6, 50.9, 48.7, 45.7, 37.9, 37.5, 35.4, 29.9, 22.0, 21.1, 3.4, 3.0. (Two distinct ^13^C signals from cyclopropyl CH_2_–groups confirmed by HSQC.)

##### HRMS (ESI)

Calc. for C_33_H_45_N_5_O_5_S [M+2H]^2+^: 311.6565, found: 311.6565.

#### 2-(6-Amino-3-iminio-4,5-disulfo-3*H*-xanthen-9-yl)-4-((3-(*N*-(3-(((2*S*,3*R*)-4-(cyclopropylamino)-3-hydroxy-1-phenylbutan-2-yl)carbamoyl)-5-(((*R*)-1-phenylethyl)carbamoyl)phenyl)-*N*-methylsulfamoyl)propyl)carbamoyl)benzoate (Alexa488-C3)

A round-bottom flask was charged with 100 μL of **9** (200 μg/100 μL DMF, 200 μg, 322 nmol, 1.0 equiv), 1.0 μL of DIPEA (740 μg, 95 μmol, 295 equiv), and 20 μL of Alexa488 NHS ester (Thermo Fisher #A20000, 100 μg/10 μL DMSO, 200 μg, 317 nmol, 1.0 equiv) were mixed at r.t. for 30 min. 250 μL of water and 250 μL of MeCN were added, and the mixture was directly subjected to RP-HPLC (MeCN:H_2_O + 0.1% TFA, gradient 10:90 → 90:10 over 45 min, flow 4 mL/min, λ = 500 nm) to obtain 193 nmol of the desired product as an orange powder after lyophilization in 62% yield. Purity: 97%.

##### HRMS (ESI)

Calc. for C_54_H_56_N_7_O_15_S_3_ [M + H]^+^: 1138.2991, found: 1138.2993.

#### 4-((3-(*N*-(3-(((2*S*,3*R*)-4-(Cyclopropylamino)-3-hydroxy-1-phenylbutan-2-yl)carbamoyl)-5-(((*R*)-1-phenylethyl)carbamoyl)phenyl)-*N*-methylsulfamoyl)propyl)carbamoyl)-2-(1,2,2,10,10,11-hexamethyl-4,8-bis(sulfomethyl)-3,4,8,9,10,11-hexahydro-2*H*-pyrano[3,2-*g*:5,6-*g’*]diquinolin-1-ium-6-yl)benzoate (Alexa568-C3)

A round-bottom flask was charged with 100 μL of **9** (200 μg/100 μL DMF, 200 μg, 322 nmol, 1.27 equiv), 1.0 μL of DIPEA (740 μg, 95 μmol, 375 equiv), and 20 μL of Alexa568 NHS ester (Thermo Fisher #A20003, 100 μg/10 μL DMSO, 200 μg, 253 nmol, 1.0 equiv) were mixed at r.t. for 30 min. 250 μL of water and 250 μL of MeCN were added, and the mixture was directly subjected to RP-HPLC (MeCN:H_2_O + 0.1% TFA, gradient 10:90 → 90:10 over 45 min, flow 4 mL/min, λ = 570 nm) to obtain 169 nmol of the desired product as a purple powder after lyophilization in 67% yield. Purity: 97%.

##### HRMS (ESI)

Calc. for C_66_H_73_N_7_O_15_S_3_ [M+2H]^2+^: 649.7158, found: 649.7154.

#### 3-(6-((3-(*N*-(3-(((2*S*,3*R*)-4-(Cyclopropylamino)-3-hydroxy-1-phenylbutan-2-yl)carbamoyl)-5-(((*R*)-1-phenylethyl)carbamoyl)phenyl)-*N*-methylsulfamoyl)propyl)amino)-6-oxohexyl)-2-((1*E*,3*E*)-5-((*E*)-3,3-dimethyl-5-sulfo-1-(3-sulfopropyl)indolin-2-ylidene)penta-1,3-dien-1-yl)-3-methyl-5-sulfo-1-(3-sulfopropyl)-3*H*-indol-1-ium (Alexa647-C3)

A round-bottom flask was charged with 22 μL of **9** (550 μg/100 μL DMF, 121 μg, 195 nmol, 1.86 equiv), 1.39 μL of DIPEA (2 μL/50 μL DMF, 41 μg, 320 nmol, 3.05 equiv), and 100 μL of Alexa647 NHS ester (Thermo Fisher #A20006, 100 μg/100 μL DMF, 100 μg, 105 nmol, 1.0 equiv) were mixed at r.t. for 30 min. 100 μL of water was added, and the mixture was directly subjected to RP-HPLC (MeCN:H_2_O + 0.1% TFA, gradient 1:99 → 90:10 over 45 min, flow 4 mL/min, λ = 650 nm) to obtain 60 nmol of the desired product as a blue powder after lyophilization in 57% yield. Purity: 99%.

##### HRMS (ESI)

Calc. for C_69_H_89_N_7_O_18_S_5_ [M + H]^2+^: 731.7428, found: 731.7423.

### Cell Lines, Plasmids, and Transfection

HEK293T cells (ATCC accession number CRL-11268) were maintained in Dulbecco's modified Eagle's medium (Sigma-Aldrich D6046) with 1g/L glucose, supplemented with 10% FCS (Merck) and 1% penicillin/streptomycin (PAA). CHO-K1 cells (ATCC #CCL-61) were maintained in modified phenol red free RPMI-1640 medium (Sigma-Aldrich, R7509), supplemented with 2 mM l-glutamine (Sigma-Aldrich, G7513), and 10% FCS (Merck). Cells were cultured at 37 °C in a 5% CO_2_ atmosphere. The following plasmids were used for transfection with the amount of DNA indicated (per 35 mm Petri dish): hBACE1 (NM_012104.4), 50 ng for single molecule recordings, 200 ng otherwise; hBACE1 fused to EGFP or mCherry, 75 ng; hBACE1 D289N fused to EGFP or mCherry, 75 ng; hBACE1 D289N, 200–400 ng; hBACE2 (NM_012105.3) fused to EGFP, 75 ng; hCathepsinD (HsCD00438327), Harvard PlasmID, 75 ng. Transfection was performed 24 h after plating using jetPEI (Polyplus) according to the manufacturer’s protocol.

### Animals

BACE1^–/–^ mice (BACE1^tm1Psa^) were generated by inserting a neo-cassette into exon 1 of the BACE1 gene introducing a premature stop codon.^19^ Mice were backcrossed on a C57BL/6J background for more than 15 generations. Mice of each sex were used for experiments. For detection of the wild-type allele or neo-cassette, PCR amplification was performed. Housing, feeding, breeding, and handling of the mice were according to federal/institutional guidelines with the approval of the local government of Unterfranken.

### Buffers

HEPES-buffered saline (HBS) contained (in mM) 150 NaCl, 3 KCl, 2 CaCl_2_, 2 MgCl_2_, 10 HEPES, and 10 d-glucose, adjusted to pH 7.4.

### Fluorescence Spectroscopy

Emission and excitation spectra of all compounds were determined at a concentration of 33 nM in phosphate buffered saline (PBS) using an infinite 200pro (Tecan) with a resolution of 2 nm. The concentration of Alexa-C3 compounds in PBS with 0.5% SDS (C. Roth) were determined using a Nanodrop 2000 (Thermo Fisher Scientific) at 495, 578, and 650 nm, respectively. For sensitivity assessment, we used PBS, PBS + BSA (0.05%), and Expi293F cells in fluorobrite (2 × 10^6^ cells/mL).

### Nanotemper

For binding analysis, the Monolith NT.115 (Nanotemper) was used. BACE-1 (mouse Protein, recombinant, life technologies, 50002-M08H-250, LCL10JA2204)) was dissolved in 50 mM HEPES/100 mM NaCl, pH 5 to a concentration of 20 μM. A dilution series was made and incubated with Alexa488-C3 0.1 nM, Alexa488 0.1 nM, or Alexa568 1 nM for 30 min at room temperature. Samples were loaded on standard capillaries (Nanotemper, MO-K022) and measured in triplicate at 22 °C, using 1%/60% LED power for Alexa488/Alexa568 and 10% MST power. Fluorescence was plotted against protein concentrations.

### Laser Scanning Microscopy

Imaging was performed with an inverse microscope AxioObserver.Z1 equipped with a LSM 780 confocal imaging module (Zeiss), a 405 nm laser diode, an argon laser LGN 3001 (LASOS), DPSS 561–10, He Ne633 laser, Plan-Apochromat 63×/1.40 oil DIC (Zeiss), LCI Plan-Apochromat 25*x*/0.8 DIC M27 (Zeiss), EC Plan-Neofluar 10×/0.3 (Zeiss), and ZEN 2010 software (Zeiss).

### Live Cell Imaging

For labeling, we plated HEK293T cells onto sterile 18 mm 1.5H borosilicate coverslips (VWR), coated with poly-d-lysine (Sigma). Cells were washed twice with PBS and incubated with Alexa-C3 inhibitor compounds for 15 min at 4 °C. In some experiments, additional 2.5 μM BACE inhibitor C3 (inhibitor IV, Merck, Calbiochem) in HBS, pH 7.4 was added. In addition, some cells were also fixed with 4% PFA or permeabilized with HBS + 50 μg/mL of Saponin for 15 min prior to staining as stated in the figure legend. After staining, live cells were washed with HBS twice. Confocal imaging was conducted at room temperature in HBS using the 63× objective and the pinhole set to 1 AU. Confocal imaging was performed 24 h after transfection.

### BACE1-Activity FRET Assay

Protease activity was determined using a FRET-based assay with recombinant BACE1 protein (Thermo Fisher Scientific; P2985). BACE1 inhibition was determined in end-points assays according to the manufacturer’s protocol except using a reduced volume of 12 μL in 384-well plates (Greiner, 788896). We observed no relevant cross-talk (<10%) between Alexa-C3 constructs and the rhodamine dye of the kit except for Alexa568-C3 and Alexa647-C3 in concentrations above 20 nM and 2000 nM, respectively. Fluorescence intensities were quantified with an infinite 200pro reader (Tecan) using top-reading mode. Data points were approximated using a logistic fit to determine half-inhibitory concentrations (pIC_50_).

### Fluorescence Correlation Spectroscopy (FCS)

Alexa-C3 compounds in HBS, pH 5 were incubated with recombinant human BACE1 protein dissolved in H_2_O (amino acids 1–457, comprising the extracellular domain, Sino Biological 10064-HCCH). For acquisition, an FCS-selected C-Apochromat 40×/1.20 objective with water immersion (Zeiss) was used. The pinhole was set to 1 AU and the point spread function was adjusted to yield maximum photon count. FCS was performed 50 μm above the glass surface in small-volume plates (Greiner 788896) at 21 ± 0.5 °C. Fluorescence intensities were recorded with a point scan at 20 MHz with the GaAsP photomultiplier (Zeiss). Experimental data were autocorrelated and fitted with one- or two-component 3D translational included in the FCS software module of ZEN2010 (Zeiss). Time-lapse recordings of slow diffusing Alexa488-C3 were fitted using linear regression. For end-point recordings, the mean effective concentration (pEC_50_) was determined with a logistic fit.

### Staining of Endogenous BACE1 in Brain Slices

Mice at postnatal day 16 (P16) were anaesthetized with Isofluran (Piramal) prior to decapitation. Whole brains were dissected, cryopreserved in 20% (w/v) sucrose for 20 h, covered with Tissue-Tek (Sakura Finetek), and incubated in −40 °C methylbutane (Roth) for 90 s. For BACE1 antibody staining whole brains were incubated in 4% PFA/PBS and cryopreserved in 20% (w/v) sucrose for 48 h before cryofixation. Frozen tissue was sliced with a microtome (Leica CM 3050S) into 14 μm sagittal sections, placed on poly d-lysine slides (VWR), and stored at −20 °C until use. Before staining, slices were thawed shortly, rehydrated with PBS, and incubated for 45 min in PBS with 5% bovine serum albumin (BSA; Sigma-Aldrich) at room temperature. To reduce the volume, slices were circumvented with silicone grease (Dow Corning). Slices were then incubated with 250 nM Alexa-C3 compound in HBS, pH 7.4, for 1 h at 4 °C in the presence or absence of 20 μM BACE1 inhibitor C3 (inhibitor IV, Merck, Calbiochem). Sections were then washed overnight at 4 °C with HBS, pH 7.4 with 5% BSA before being mounted with DAPI-containing medium (C. Roth) and sealed with Twinsil (Picodent). For BACE1 labeling with antibodies, brain slices were permeabilized for 30 min with PBS containing 0.5% Triton X-100 and permeabilized subsequently with PBS containing 1% BSA (w/v), 5% donkey normal serum, and 0.1% Triton X-100. Brain slices were incubated at room temperature overnight with primary antibody (monoclonal; rabbit anti-BACE (D10E5); 1:100 or 1:250; Cell Signaling; #5606), diluted in PBS containing 0.1% Triton X-100 and 1% BSA. Slides were washed three times in PBS and incubated in secondary antibody (monoclonal; donkey anti-rabbit Alexa-Fluor 488; 1:500; Molecular Probes) at room temperature for 60 min in PBS containing 0.1% Triton X-100 and 1% BSA. Slides were rinsed three times with PBS, mounted and sealed.

For quantification of hippocampal BACE1 staining, mean intensities of the mossy fiber bundle were divided by the mean intensity of the molecular layer of the dentate gyrus. Mean intensities were calculated from rectangular regions of interest. In addition, analysis was performed on images of hippocampal BACE1 labeling with SiR-BACE1 recorded previously.^31^

### Acceptor-Photobleaching FRET in HEK293T Cells

HEK293T cells were plated on poly-d-lysine-coated 1.5H borosilicate coverslips (VWR) and transfected the next day using jetPEI (Polyplus-transfection) with 200 ng of each plasmid. After 48 h, cells were fixed for 15 min with PBS with 4% PFA (C. Roth) and washed three times with PBS before staining with 300 nM Alexa568-C3 and 100 nM Alexa488-C3 for 15 min at room temperature. Coverslips were mounted with Roti-Mount Fluor Care (C. Roth), sealed with Twinsil (Picodent) and stored at 4 °C for up to 48 h before imaging. Single cells were imaged using the 63×/1.40 oil Plan-Apochromat (Zeiss). Acceptor-photobleaching of Alexa568-C3 was performed with the 561 nm laser. Emission was analyzed from two channels set between 516 and 524 nm and 595–603 nm, respectively. Exposure time, laser intensity, and gain settings were kept constant for all experiments. The background fluorescence was subtracted using a cell free region. Correction of acceptor-/donor-ratio fluctuations was established with an additional correction factor, provided in an additional experiment. In this experiment, cells with similar intensity levels in the donor channel (±2%) and variable intensity levels in the acceptor channel were measured. The slope of the linear regression provides the correction factor. Confocal imaging was performed 48 h after transfection.

### Total Internal Reflection Fluorescence (TIRF) Microscopy

CHO K1 cells were seeded 72 h prior to recordings onto fibronectin-coated (Sigma-Aldrich, F1141) Glass Bottom dishes (Greiner, 627861) and transfected 48 h before imaging with 50 ng of wild-type hBACE1 using jetPEI (Polyplus). Sample preparation was performed on ice directly preceding imaging. Cells were incubated with 200 nM Alexa-C3 compounds in PBS for 30 min and fixed using 4% PFA/0.2% glutaraldehyde solution^65^ and then imaged in HBS pH 7.4. After each step, samples were washed multiple times using ice-cold PBS pH 7.4.

Single molecule TIRF microscopy was performed using an inverse-stage TIRF setup (Nikon Eclipse Ti-E) using a 488 nm laser (Coherent, 150 mW) set to 20% power output and a 561 nm laser (Coherent, 50 mW). Excitation light was circularly polarized using an achromatic λ/4 wave plate (Thorlabs, AQWP05M-630) in order to reduce variation in fluorophore emission.^64^ The microscope was equipped with a dual-band 488/561 beam splitter (AHF Analysentechnik), ET GFP/mCherry dual-band emission filter (AHF Analysentechnik), Apo TIRF 100×/NA 1,49 oil immersion DIC objective (Nikon), and a iXon DU-897 back illuminated EMCCD camera (Andor Technology). After selecting cells with well distinguishable fluorescence spots, time lapses of 1000 frames with 70 ms exposure time were recorded utilizing the camera’s full, unbinned EMCCD chip with EM-gain set to 300 and cooled down to −100 °C.

### Single Fluorophore Detection and Bleaching Step Analysis

Time-lapse recordings were background-subtracted using ImageJ^45^ (rolling ball radius 50 pixel). Intensities over time of single fluorescent spots were extracted using GMimPro (version 2022)^46^ using the following settings: 3 × 3 spots, threshold 25, and no background subtraction. From time series, bleaching step detection was performed using the python quickPBSA package.^47^ Settings were as follows: threshold 75, subtracted false, length_laststep 10, percentile_step [0, 95], mult_threshold 2, maxmult 1. All bleaching steps were manually reviewed. From the bleaching step analysis, the mean relative frequencies were determined. We approximated the obtained histogram with a sum of binomial distributions (eq 1) utilizing the COBYLA algorithm^66^ within the scipy optimization package^67^:
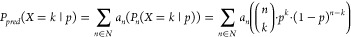
1with *P*_*pred*_(X = *k* | *p*) being the probability to observe a number of bleaching steps of BACE1 multimers. The order *n* of the binomial functions was set according to the possible number *n* ∈ *N* of BACE1 molecules in a diffraction-limited spot, i.e., *N* = {2,4,6} for BACE1 dimers, *N* = {3,6} for trimers and *N* = {2,3,4,5,6} for a combination of dimers and trimers. Bleaching step counts *k* > 6 were excluded. The fit was repeated varying the labeling efficiency *p* in 0.01 increments. The mismatch between prediction and observed distribution is represented by the sum of squared errors (SSE; eq 2):
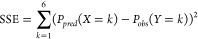
2

### Statistical Analysis

Data are presented as mean ± SEM if not otherwise stated. Statistical significance between means was calculated using the two-tailed Student′s *t* test.

## Supporting Information Available

NMR spectra of all compounds, purity assessment for Alexa488-C3, Alexa568-C3, and Alexa647-C3 via LCMS; Supplemental Figures 1–3 (PDF)
Molecular formular strings and values for *IC*_50_ and *K*_D_ measurements(CSV)

## ABBREVIATIONS USED

Aβ: amyloid β
AD: Alzheimer′s disease
APP: amyloid precursor protein
AU: arbitrary units
BACE1: β-site of APP cleaving enzyme 1
BSA: bovine serum albumin
C3: BACE1-inhibitor IV
CA: cornu ammonis
CHO-K1 cells: Chinese hamster ovary K1 cells
DG: dentate gyrus
ECD: extracellular domain
EGFP: enhanced green fluorescent protein
FCS: fluorescence correlation spectroscopy
FRET: Förster resonance energy transfer
HBS: HEPES-buffered saline
HEK-293T cells: human embryonic kidney cells 293 with SV40 large T antigen
IHC: immunohistochemistry
KCNQ: voltage-gated potassium channel subfamily
LSM: laser scanning microscope
MFB: mossy fiber bundle
msfGFP: monomeric green fluorescent protein superfolder
PBS: phosphate-buffered saline
PML: plasma membrane lawn
SD: standard deviation
SEM: standard error of the mean
SiR647: silicone rhodamine 647
SSE: sum of squared errors
STED: stimulated emission depletion
TIRF: total internal reflection fluorescence

## References

[ref1] Hardy J. A.; Higgins G. A. Alzheimer’s Disease: The Amyloid Cascade Hypothesis. Science 1992, 256 (5054), 184–185. 10.1126/science.1566067. 1566067

[ref2] Selkoe D. J.; Hardy J. The Amyloid Hypothesis of Alzheimer’s Disease at 25 Years. EMBO Mol. Med. 2016, 8 (6), 595–608. 10.15252/emmm.201606210. 27025652 PMC4888851

[ref3] Vassar R.; Bennett B. D.; Babu-Khan S.; Kahn S.; Mendiaz E. A.; Denis P.; Teplow D. B.; Ross S.; Amarante P.; Loeloff R.; Luo Y.; Fisher S.; Fuller J.; Edenson S.; Lile J.; Jarosinski M. A.; Biere A. L.; Curran E.; Burgess T.; Louis J. C.; Collins F.; Treanor J.; Rogers G.; Citron M. Beta-Secretase Cleavage of Alzheimer’s Amyloid Precursor Protein by the Transmembrane Aspartic Protease BACE. Science 1999, 286 (5440), 735–741. 10.1126/science.286.5440.735. 10531052

[ref4] Hussain I.; Powell D.; Howlett D. R.; Tew D. G.; Meek T. D.; Chapman C.; Gloger I. S.; Murphy K. E.; Southan C. D.; Ryan D. M.; Smith T. S.; Simmons D. L.; Walsh F. S.; Dingwall C.; Christie G. Identification of a Novel Aspartic Protease (Asp 2) as Beta-Secretase. Mol. Cell. Neurosci. 1999, 14 (6), 419–427. 10.1006/mcne.1999.0811. 10656250

[ref5] Sinha S.; Anderson J. P.; Barbour R.; Basi G. S.; Caccavello R.; Davis D.; Doan M.; Dovey H. F.; Frigon N.; Hong J.; Jacobson-Croak K.; Jewett N.; Keim P.; Knops J.; Lieberburg I.; Power M.; Tan H.; Tatsuno G.; Tung J.; Schenk D.; Seubert P.; Suomensaari S. M.; Wang S.; Walker D.; Zhao J.; McConlogue L.; John V. Purification and Cloning of Amyloid Precursor Protein Beta-Secretase from Human Brain. Nature 1999, 402 (6761), 537–540. 10.1038/990114. 10591214

[ref6] Yan R.; Bienkowski M. J.; Shuck M. E.; Miao H.; Tory M. C.; Pauley A. M.; Brashler J. R.; Stratman N. C.; Mathews W. R.; Buhl A. E.; Carter D. B.; Tomasselli A. G.; Parodi L. A.; Heinrikson R. L.; Gurney M. E. Membrane-Anchored Aspartyl Protease with Alzheimer’s Disease β-Secretase Activity. Nature 1999, 402 (6761), 533–537. 10.1038/990107. 10591213

[ref7] Yan R.; Vassar R. Targeting the β Secretase BACE1 for Alzheimer’s Disease Therapy. Lancet. Neurol. 2014, 13 (3), 319–329. 10.1016/S1474-4422(13)70276-X. 24556009 PMC4086426

[ref8] Abbott A. Conquering Alzheimer’s: A Look at the Therapies of the Future. Nature 2023, 616 (7955), 26–28. 10.1038/d41586-023-00954-w. 37016121

[ref9] Bazzari F. H.; Bazzari A. H. BACE1 Inhibitors for Alzheimer’s Disease: The Past, Present and Any Future? Molecules 2022, 27 ( (24), ). 8823 10.3390/molecules27248823. 36557955 PMC9785888

[ref10] McDade E.; Voytyuk I.; Aisen P.; Bateman R. J.; Carrillo M. C.; De Strooper B.; Haass C.; Reiman E. M.; Sperling R.; Tariot P. N.; Yan R.; Masters C. L.; Vassar R.; Lichtenthaler S. F. The Case for Low-Level BACE1 Inhibition for the Prevention of Alzheimer Disease. Nat. Rev. Neurol. 2021, 17 (11), 703–714. 10.1038/s41582-021-00545-1. 34548654

[ref11] Karisetty B. C.; Bhatnagar A.; Armour E. M.; Beaver M.; Zhang H.; Elefant F. Amyloid-β Peptide Impact on Synaptic Function and Neuroepigenetic Gene Control Reveal New Therapeutic Strategies for Alzheimer’s Disease. Front. Mol. Neurosci. 2020, 13, 577622 10.3389/fnmol.2020.577622. 33304239 PMC7693454

[ref12] Zhu K.; Xiang X.; Filser S.; Marinković P.; Dorostkar M. M.; Crux S.; Neumann U.; Shimshek D. R.; Rammes G.; Haass C.; Lichtenthaler S. F.; Gunnersen J. M.; Herms J. Beta-Site Amyloid Precursor Protein Cleaving Enzyme 1 Inhibition Impairs Synaptic Plasticity via Seizure Protein 6. Biol. Psychiatry 2018, 83 (5), 428–437. 10.1016/j.biopsych.2016.12.023. 28129943

[ref13] Das B.; Singh N.; Yao A. Y.; Zhou J.; He W.; Hu X.; Yan R. BACE1 Controls Synaptic Function through Modulating Release of Synaptic Vesicles. Mol. Psychiatry 2021, 26 (11), 6394–6410. 10.1038/s41380-021-01166-2. 34158621 PMC8760050

[ref14] Das B.; Yan R. Role of BACE1 in Alzheimer’s Synaptic Function. Transl. Neurodegener. 2017, 6, 23 10.1186/s40035-017-0093-5. 28855981 PMC5575945

[ref15] Hitt B. D.; Jaramillo T. C.; Chetkovich D. M.; Vassar R. BACE1–/– Mice Exhibit Seizure Activity That Does Not Correlate with Sodium Channel Level or Axonal Localization. Mol. Neurodegener. 2010, 5, 31 10.1186/1750-1326-5-31. 20731874 PMC2933677

[ref16] Cheret C.; Willem M.; Fricker F. R.; Wende H.; Wulf-Goldenberg A.; Tahirovic S.; Nave K.-A.; Saftig P.; Haass C.; Garratt A. N.; Bennett D. L.; Birchmeier C. Bace1 and Neuregulin-1 Cooperate to Control Formation and Maintenance of Muscle Spindles. EMBO J. 2013, 32 (14), 2015–2028. 10.1038/emboj.2013.146. 23792428 PMC3715864

[ref17] Hu X.; Das B.; Hou H.; He W.; Yan R. BACE1 Deletion in the Adult Mouse Reverses Preformed Amyloid Deposition and Improves Cognitive Functions. J. Exp. Med. 2018, 215 (3), 927–940. 10.1084/jem.20171831. 29444819 PMC5839766

[ref18] Taylor H. A.; Przemylska L.; Clavane E. M.; Meakin P. J. BACE1: More than Just a β-Secretase. Obes. Rev. 2022, 23 (7), e13430 10.1111/obr.13430. 35119166 PMC9286785

[ref19] Dominguez D.; Tournoy J.; Hartmann D.; Huth T.; Cryns K.; Deforce S.; Serneels L.; Camacho I. E.; Marjaux E.; Craessaerts K.; Roebroek A. J. M.; Schwake M.; D’Hooge R.; Bach P.; Kalinke U.; Moechars D.; Alzheimer C.; Reiss K.; Saftig P.; De Strooper B. Phenotypic and Biochemical Analyses of BACE1- and BACE2-Deficient Mice*. J. Biol. Chem. 2005, 280 (35), 30797–30806. 10.1074/jbc.M505249200. 15987683

[ref20] Dierich M.; Hartmann S.; Dietrich N.; Moeser P.; Brede F.; Johnson Chacko L.; Tziridis K.; Schilling A.; Krauss P.; Hessler S.; Karch S.; Schrott-Fischer A.; Blumer M.; Birchmeier C.; Oliver D.; Moser T.; Schulze H.; Alzheimer C.; Leitner M. G.; Huth T. β-Secretase BACE1 Is Required for Normal Cochlear Function. J. Neurosci. 2019, 39 (45), 9013–9027. 10.1523/JNEUROSCI.0028-19.2019. 31527119 PMC6832675

[ref21] Hefter D.; Ludewig S.; Draguhn A.; Korte M. Amyloid, APP, and Electrical Activity of the Brain. Neuroscientist 2020, 26 (3), 231–251. 10.1177/1073858419882619. 31779518 PMC7222965

[ref22] Lehnert S.; Hartmann S.; Hessler S.; Adelsberger H.; Huth T.; Alzheimer C. Ion Channel Regulation by β-Secretase BACE1 - Enzymatic and Non-Enzymatic Effects beyond Alzheimer’s Disease. Channels (Austin). 2016, 10 (5), 365–378. 10.1080/19336950.2016.1196307. 27253079 PMC5001140

[ref23] Hartmann S.; Zheng F.; Kyncl M. C.; Karch S.; Voelkl K.; Zott B.; D’Avanzo C.; Lomoio S.; Tesco G.; Kim D. Y.; Alzheimer C.; Huth T. β-Secretase BACE1 Promotes Surface Expression and Function of Kv3.4 at Hippocampal Mossy Fiber Synapses. J. Neurosci. 2018, 38 (14), 3480–3494. 10.1523/JNEUROSCI.2643-17.2018. 29507146 PMC5895038

[ref24] Gazzi T.; Brennecke B.; Atz K.; Korn C.; Sykes D.; Forn-Cuni G.; Pfaff P.; Sarott R. C.; Westphal M. V.; Mostinski Y.; Mach L.; Wasinska-Kalwa M.; Weise M.; Hoare B. L.; Miljuš T.; Mexi M.; Roth N.; Koers E. J.; Guba W.; Alker A.; Rufer A. C.; Kusznir E. A.; Huber S.; Raposo C.; Zirwes E. A.; Osterwald A.; Pavlovic A.; Moes S.; Beck J.; Nettekoven M.; Benito-Cuesta I.; Grande T.; Drawnel F.; Widmer G.; Holzer D.; van der Wel T.; Mandhair H.; Honer M.; Fingerle J.; Scheffel J.; Broichhagen J.; Gawrisch K.; Romero J.; Hillard C. J.; Varga Z. V.; van der Stelt M.; Pacher P.; Gertsch J.; Ullmer C.; McCormick P. J.; Oddi S.; Spaink H. P.; Maccarrone M.; Veprintsev D. B.; Carreira E. M.; Grether U.; Nazaré M. Detection of Cannabinoid Receptor Type 2 in Native Cells and Zebrafish with a Highly Potent, Cell-Permeable Fluorescent Probe. Chem. Sci. 2022, 13 (19), 5539–5545. 10.1039/D1SC06659E. 35694350 PMC9116301

[ref25] Sarott R. C.; Westphal M. V.; Pfaff P.; Korn C.; Sykes D. A.; Gazzi T.; Brennecke B.; Atz K.; Weise M.; Mostinski Y.; Hompluem P.; Koers E.; Miljuš T.; Roth N. J.; Asmelash H.; Vong M. C.; Piovesan J.; Guba W.; Rufer A. C.; Kusznir E. A.; Huber S.; Raposo C.; Zirwes E. A.; Osterwald A.; Pavlovic A.; Moes S.; Beck J.; Benito-Cuesta I.; Grande T.; Ruiz de Martín Esteban S.; Yeliseev A.; Drawnel F.; Widmer G.; Holzer D.; van der Wel T.; Mandhair H.; Yuan C. Y.; Drobyski W. R.; Saroz Y.; Grimsey N.; Honer M.; Fingerle J.; Gawrisch K.; Romero J.; Hillard C. J.; Varga Z. V.; van der Stelt M.; Pacher P.; Gertsch J.; McCormick P. J.; Ullmer C.; Oddi S.; Maccarrone M.; Veprintsev D. B.; Nazaré M.; Grether U.; Carreira E. M. Development of High-Specificity Fluorescent Probes to Enable Cannabinoid Type 2 Receptor Studies in Living Cells. J. Am. Chem. Soc. 2020, 142 (40), 16953–16964. 10.1021/jacs.0c05587. 32902974

[ref26] Ast J.; Novak A. N.; Podewin T.; Fine N. H. F.; Jones B.; Tomas A.; Birke R.; Roßmann K.; Mathes B.; Eichhorst J.; Lehmann M.; Linnemann A. K.; Hodson D. J.; Broichhagen J. Expanded LUXendin Color Palette for GLP1R Detection and Visualization In Vitro and In Vivo. JACS Au 2022, 2 (4), 1007–1017. 10.1021/jacsau.2c00130. 35557759 PMC9088800

[ref27] Huber M. E.; Wurnig S.; Toy L.; Weiler C.; Merten N.; Kostenis E.; Hansen F. K.; Schiedel M. Fluorescent Ligands Enable Target Engagement Studies for the Intracellular Allosteric Binding Site of the Chemokine Receptor CXCR2. J. Med. Chem. 2023, 66 (14), 9916–9933. 10.1021/acs.jmedchem.3c00769. 37463496 PMC10388362

[ref28] Jatzlau J.; Burdzinski W.; Trumpp M.; Obendorf L.; Roßmann K.; Ravn K.; Hyvönen M.; Bottanelli F.; Broichhagen J.; Knaus P. A Versatile Halo- and SNAP-Tagged BMP/TGFβ Receptor Library for Quantification of Cell Surface Ligand Binding. Commun. Biol. 2023, 6 (1), 34 10.1038/s42003-022-04388-4. 36635368 PMC9837045

[ref29] Fang M.; Wu O.; Cupp-Sutton K. A.; Smith K.; Wu S. Elucidating Protein-Ligand Interactions in Cell Lysates Using High-Throughput Hydrogen-Deuterium Exchange Mass Spectrometry with Integrated Protein Thermal Depletion. Anal. Chem. 2023. 95 1805 10.1021/acs.analchem.2c04266. PMC10323047 36608260

[ref30] Elek M.; Dubiel M.; Mayer L.; Zivkovic A.; Müller T. J. J.; Stark H. BOPPY-Based Novel Fluorescent Dopamine D2 and D3 Receptor Ligands. Bioorg. Med. Chem. Lett. 2022, 59, 128573 10.1016/j.bmcl.2022.128573. 35063632

[ref31] Karch S.; Broichhagen J.; Schneider J.; Böning D.; Hartmann S.; Schmid B.; Tripal P.; Palmisano R.; Alzheimer C.; Johnsson K.; Huth T. A New Fluorogenic Small-Molecule Labeling Tool for Surface Diffusion Analysis and Advanced Fluorescence Imaging of β-Site Amyloid Precursor Protein-Cleaving Enzyme 1 Based on Silicone Rhodamine: SiR-BACE1. J. Med. Chem. 2018, 61 (14), 6121–6139. 10.1021/acs.jmedchem.8b00387. 29939737

[ref32] Malamas M. S.; Robichaud A.; Erdei J.; Quagliato D.; Solvibile W.; Zhou P.; Morris K.; Turner J.; Wagner E.; Fan K.; Olland A.; Jacobsen S.; Reinhart P.; Riddell D.; Pangalos M. Design and Synthesis of Aminohydantoins as Potent and Selective Human β-Secretase (BACE1) Inhibitors with Enhanced Brain Permeability. Bioorg. Med. Chem. Lett. 2010, 20 (22), 6597–6605. 10.1016/j.bmcl.2010.09.029. 20880704

[ref33] Lukinavičius G.; Umezawa K.; Olivier N.; Honigmann A.; Yang G.; Plass T.; Mueller V.; Reymond L.; Corrêa I. R.; Luo Z.-G.; Schultz C.; Lemke E. A.; Heppenstall P.; Eggeling C.; Manley S.; Johnsson K. A Near-Infrared Fluorophore for Live-Cell Super-Resolution Microscopy of Cellular Proteins. Nat. Chem. 2013, 5 (2), 132–139. 10.1038/nchem.1546. 23344448

[ref34] Stachel S. J.; Coburn C. A.; Steele T. G.; Jones K. G.; Loutzenhiser E. F.; Gregro A. R.; Rajapakse H. A.; Lai M.-T.; Crouthamel M.-C.; Xu M.; Tugusheva K.; Lineberger J. E.; Pietrak B. L.; Espeseth A. S.; Shi X.-P.; Chen-Dodson E.; Holloway M. K.; Munshi S.; Simon A. J.; Kuo L.; Vacca J. P. Structure-Based Design of Potent and Selective Cell-Permeable Inhibitors of Human Beta-Secretase (BACE-1). J. Med. Chem. 2004, 47 (26), 6447–6450. 10.1021/jm049379g. 15588077

[ref35] Stachel S. J.; Coburn C. A.; Sankaranarayanan S.; Price E. A.; Wu G.; Crouthamel M.; Pietrak B. L.; Huang Q.; Lineberger J.; Espeseth A. S.; Jin L.; Ellis J.; Holloway M. K.; Munshi S.; Allison T.; Hazuda D.; Simon A. J.; Graham S. L.; Vacca J. P. Macrocyclic Inhibitors of Beta-Secretase: Functional Activity in an Animal Model. J. Med. Chem. 2006, 49 (21), 6147–6150. 10.1021/jm060884i. 17034118

[ref36] Hessler S.; Zheng F.; Hartmann S.; Rittger A.; Lehnert S.; Völkel M.; Nissen M.; Edelmann E.; Saftig P.; Schwake M.; Huth T.; Alzheimer C. β-Secretase BACE1 Regulates Hippocampal and Reconstituted M-Currents in a β-Subunit-like Fashion. J. Neurosci. 2015, 35 (8), 3298–3311. 10.1523/JNEUROSCI.3127-14.2015. 25716831 PMC6605557

[ref37] Huth T.; Schmidt-Neuenfeldt K.; Rittger A.; Saftig P.; Reiss K.; Alzheimer C. Non-Proteolytic Effect of Beta-Site APP-Cleaving Enzyme 1 (BACE1) on Sodium Channel Function. Neurobiol. Dis. 2009, 33 (2), 282–289. 10.1016/j.nbd.2008.10.015. 19056495

[ref38] Kandalepas P. C.; Sadleir K. R.; Eimer W. A.; Zhao J.; Nicholson D. A.; Vassar R. The Alzheimer’s β-Secretase BACE1 Localizes to Normal Presynaptic Terminals and to Dystrophic Presynaptic Terminals Surrounding Amyloid Plaques. Acta Neuropathol. 2013, 126 (3), 329–352. 10.1007/s00401-013-1152-3. 23820808 PMC3753469

[ref39] Causevic M.; Dominko K.; Malnar M.; Vidatic L.; Cermak S.; Pigoni M.; Kuhn P.-H.; Colombo A.; Havas D.; Flunkert S.; McDonald J.; Gunnersen J. M.; Hutter-Paier B.; Tahirovic S.; Windisch M.; Krainc D.; Lichtenthaler S. F.; Hecimovic S. BACE1-Cleavage of Sez6 and Sez6L Is Elevated in Niemann-Pick Type C Disease Mouse Brains. PLoS One 2018, 13 (7), e0200344 10.1371/journal.pone.0200344. 29979789 PMC6034874

[ref40] Voytyuk I.; Mueller S. A.; Herber J.; Snellinx A.; Moechars D.; van Loo G.; Lichtenthaler S. F.; De Strooper B. BACE2 Distribution in Major Brain Cell Types and Identification of Novel Substrates. Life Sci. 2018, 1 (1), e201800026 10.26508/lsa.201800026. PMC6238391 30456346

[ref41] Liebsch F.; Aurousseau M. R. P.; Bethge T.; McGuire H.; Scolari S.; Herrmann A.; Blunck R.; Bowie D.; Multhaup G. Full-Length Cellular β-Secretase Has a Trimeric Subunit Stoichiometry, and Its Sulfur-Rich Transmembrane Interaction Site Modulates Cytosolic Copper Compartmentalization. J. Biol. Chem. 2017, 292 (32), 13258–13270. 10.1074/jbc.M117.779165. 28637867 PMC5555187

[ref42] Westmeyer G. G.; Willem M.; Lichtenthaler S. F.; Lurman G.; Multhaup G.; Assfalg-Machleidt I.; Reiss K.; Saftig P.; Haass C. Dimerization of Beta-Site Beta-Amyloid Precursor Protein-Cleaving Enzyme. J. Biol. Chem. 2004, 279 (51), 53205–53212. 10.1074/jbc.M410378200. 15485862

[ref43] Wu P.; Brand L. Resonance Energy Transfer: Methods and Applications. Anal. Biochem. 1994, 218 (1), 1–13. 10.1006/abio.1994.1134. 8053542

[ref44] Berney C.; Danuser G. FRET or No FRET: A Quantitative Comparison. Biophys. J. 2003, 84 (6), 3992–4010. 10.1016/S0006-3495(03)75126-1. 12770904 PMC1302980

[ref45] Schneider C. A.; Rasband W. S.; Eliceiri K. W. NIH Image to ImageJ: 25 Years of Image Analysis. Nat. Methods 2012, 9 (7), 671–675. 10.1038/nmeth.2089. 22930834 PMC5554542

[ref46] Mashanov G. I.; Molloy J. E. Automatic Detection of Single Fluorophores in Live Cells. Biophys. J. 2007, 92 (6), 2199–2211. 10.1529/biophysj.106.081117. 17208981 PMC1861788

[ref47] Hummert J.; Yserentant K.; Fink T.; Euchner J.; Ho Y. X.; Tashev S. A.; Herten D.-P. Photobleaching Step Analysis for Robust Determination of Protein Complex Stoichiometries. Mol. Biol. Cell 2021, 32 (21), ar35 10.1091/mbc.E20-09-0568. 34586828 PMC8693960

[ref48] McGuire H.; Aurousseau M. R. P.; Bowie D.; Blunck R. Automating Single Subunit Counting of Membrane Proteins in Mammalian Cells. J. Biol. Chem. 2012, 287 (43), 35912–35921. 10.1074/jbc.M112.402057. 22930752 PMC3476259

[ref49] Hampel H.; Vassar R.; De Strooper B.; Hardy J.; Willem M.; Singh N.; Zhou J.; Yan R.; Vanmechelen E.; De Vos A.; Nisticò R.; Corbo M.; Imbimbo B. P.; Streffer J.; Voytyuk I.; Timmers M.; Tahami Monfared A. A.; Irizarry M.; Albala B.; Koyama A.; Watanabe N.; Kimura T.; Yarenis L.; Lista S.; Kramer L.; Vergallo A. The β-Secretase BACE1 in Alzheimer’s Disease. Biol. Psychiatry 2021, 89 (8), 745–756. 10.1016/j.biopsych.2020.02.001. 32223911 PMC7533042

[ref50] Frisoni G. B.; Altomare D.; Thal D. R.; Ribaldi F.; van der Kant R.; Ossenkoppele R.; Blennow K.; Cummings J.; van Duijn C.; Nilsson P. M.; Dietrich P.-Y.; Scheltens P.; Dubois B. The Probabilistic Model of Alzheimer Disease: The Amyloid Hypothesis Revised. Nat. Rev. Neurosci. 2022, 23 (1), 53–66. 10.1038/s41583-021-00533-w. 34815562 PMC8840505

[ref51] Malamas M. S.; Barnes K.; Hui Y.; Johnson M.; Lovering F.; Condon J.; Fobare W.; Solvibile W.; Turner J.; Hu Y.; Manas E. S.; Fan K.; Olland A.; Chopra R.; Bard J.; Pangalos M. N.; Reinhart P.; Robichaud A. J. Novel Pyrrolyl 2-Aminopyridines as Potent and Selective Human Beta-Secretase (BACE1) Inhibitors. Bioorg. Med. Chem. Lett. 2010, 20 (7), 2068–2073. 10.1016/j.bmcl.2010.02.075. 20223661

[ref52] Tan J. Z. A.; Fourriere L.; Wang J.; Perez F.; Boncompain G.; Gleeson P. A. Distinct Anterograde Trafficking Pathways of BACE1 and Amyloid Precursor Protein from the TGN and the Regulation of Amyloid-β Production. Mol. Biol. Cell 2020, 31 (1), 27–44. 10.1091/mbc.E19-09-0487. 31746668 PMC6938271

[ref53] Birke R.; Ast J.; Roosen D. A.; Lee J.; Roßmann K.; Huhn C.; Mathes B.; Lisurek M.; Bushiri D.; Sun H.; Jones B.; Lehmann M.; Levitz J.; Haucke V.; Hodson D. J.; Broichhagen J. Sulfonated Red and Far-Red Rhodamines to Visualize SNAP- and Halo-Tagged Cell Surface Proteins. Org. Biomol. Chem. 2022, 20 (30), 5967–5980. 10.1039/D1OB02216D. 35188523 PMC9346974

[ref54] Zhao J.; Fu Y.; Yasvoina M.; Shao P.; Hitt B.; O’Connor T.; Logan S.; Maus E.; Citron M.; Berry R.; Binder L.; Vassar R. Beta-Site Amyloid Precursor Protein Cleaving Enzyme 1 Levels Become Elevated in Neurons around Amyloid Plaques: Implications for Alzheimer’s Disease Pathogenesis. J. Neurosci. 2007, 27 (14), 3639–3649. 10.1523/JNEUROSCI.4396-06.2007. 17409228 PMC6672403

[ref55] Kuhn P.-H.; Koroniak K.; Hogl S.; Colombo A.; Zeitschel U.; Willem M.; Volbracht C.; Schepers U.; Imhof A.; Hoffmeister A.; Haass C.; Roßner S.; Bräse S.; Lichtenthaler S. F. Secretome Protein Enrichment Identifies Physiological BACE1 Protease Substrates in Neurons. EMBO J. 2012, 31 (14), 3157–3168. 10.1038/emboj.2012.173. 22728825 PMC3400020

[ref56] Müller S. A.; Shmueli M. D.; Feng X.; Tüshaus J.; Schumacher N.; Clark R.; Smith B. E.; Chi A.; Rose-John S.; Kennedy M. E.; Lichtenthaler S. F. The Alzheimer’s Disease-Linked Protease BACE1Modulates Neuronal IL-6 Signaling through Shedding of the Receptor Gp130. Mol. Neurodegener. 2023, 18 (1), 13 10.1186/s13024-023-00596-6. 36810097 PMC9942414

[ref57] Zhou L.; Barão S.; Laga M.; Bockstael K.; Borgers M.; Gijsen H.; Annaert W.; Moechars D.; Mercken M.; Gevaer K.; De Strooper B. The Neural Cell Adhesion Molecules L1 and CHL1 Are Cleaved by BACE1 Protease in Vivo. J. Biol. Chem. 2012, 287 (31), 25927–25940. 10.1074/jbc.M112.377465. 22692213 PMC3406677

[ref58] Kim D. Y.; Carey B. W.; Wang H.; Ingano L. A. M.; Binshtok A. M.; Wertz M. H.; Pettingell W. H.; He P.; Lee V. M.-Y.; Woolf C. J.; Kovacs D. M. BACE1 Regulates Voltage-Gated Sodium Channels and Neuronal Activity. Nat. Cell Biol. 2007, 9 (7), 755–764. 10.1038/ncb1602. 17576410 PMC2747787

[ref59] Wang X.; Wang C.; Pei G. α-Secretase ADAM10 Physically Interacts with β-Secretase BACE1 in Neurons and Regulates CHL1 Proteolysis. J. Mol. Cell Biol. 2018, 10 (5), 411–422. 10.1093/jmcb/mjy001. 29325091

[ref60] Liu L.; Ding L.; Rovere M.; Wolfe M. S.; Selkoe D. J. A Cellular Complex of BACE1 and γ-Secretase Sequentially Generates Aβ from Its Full-Length Precursor. J. Cell Biol. 2019, 218 (2), 644–663. 10.1083/jcb.201806205. 30626721 PMC6363461

[ref61] Jin S.; Agerman K.; Kolmodin K.; Gustafsson E.; Dahlqvist C.; Jureus A.; Liu G.; Fälting J.; Berg S.; Lundkvist J.; Lendahl U. Evidence for Dimeric BACE-Mediated APP Processing. Biochem. Biophys. Res. Commun. 2010, 393 (1), 21–27. 10.1016/j.bbrc.2010.01.064. 20097169

[ref62] Multhaup G. Amyloid Precursor Protein and BACE Function as Oligomers. Neurodegener. Dis. 2006, 3 (4–5), 270–274. 10.1159/000095266. 17047367

[ref63] Schmechel A.; Strauss M.; Schlicksupp A.; Pipkorn R.; Haass C.; Bayer T. A.; Multhaup G. Human BACE Forms Dimers and Colocalizes with APP. J. Biol. Chem. 2004, 279 (38), 39710–39717. 10.1074/jbc.M402785200. 15247262

[ref64] Tian Y.; Halle J.; Wojdyr M.; Sahoo D.; Scheblykin I. G. Quantitative Measurement of Fluorescence Brightness of Single Molecules. Methods Appl. Fluoresc. 2014, 2 (3), 035003 10.1088/2050-6120/2/3/035003. 29148471

[ref65] Tanaka K. A. K.; Suzuki K. G. N.; Shirai Y. M.; Shibutani S. T.; Miyahara M. S. H.; Tsuboi H.; Yahara M.; Yoshimura A.; Mayor S.; Fujiwara T. K.; Kusumi A. Membrane Molecules Mobile Even after Chemical Fixation. Nat. Methods 2010, 7 (11), 865–866. 10.1038/nmeth.f.314. 20881966

[ref66] Powell M. J. D. A Direct Search Optimization Method That Models the Objective and Constraint Functions by Linear Interpolation; Springer, 1994.

[ref67] Virtanen P.; Gommers R.; Oliphant T. E.; Haberland M.; Reddy T.; Cournapeau D.; Burovski E.; Peterson P.; Weckesser W.; Bright J.; van der Walt S. J.; Brett M.; Wilson J.; Millman K. J.; Mayorov N.; Nelson A. R. J.; Jones E.; Kern R.; Larson E.; Carey C. J.; Polat İ.; Feng Y.; Moore E. W.; VanderPlas J.; Laxalde D.; Perktold J.; Cimrman R.; Henriksen I.; Quintero E. A.; Harris C. R.; Archibald A. M.; Ribeiro A. H.; Pedregosa F.; van Mulbregt P. SciPy 1.0 Contributors. SciPy 1.0: Fundamental Algorithms for Scientific Computing in Python. Nat. Methods 2020, 17 (3), 352–272. 10.1038/s41592-020-0772-5. 32015543 PMC7056644

