# Supplementary material for: Multicolor, Cell-Impermeable, and High Affinity BACE1 Inhibitor Probes Enable Superior Endogenous Staining and Imaging of Single Molecules
Source: J Med Chem. 2024 Jun 6;67(12):10152–67. doi: 10.1021/acs.jmedchem.4c00339 (PMC11215771; doi:10.1021/acs.jmedchem.4c00339)
Supplement: Supplementary file 1 — jm4c00339_si_001.pdf [file jm4c00339_si_001.pdf]

SUPPORTING INFORMATION

Multi-color, cell-impermeable and high affinity BACE1 inhibitor probes enable superior endogenous staining and imaging of single molecules

Florian Stockinger[1], Pascal Poc[2,3], Alexander Möhwald[1], Sandra Karch[1], Stephanie Häfner[4,5], Christian Alzheimer[1], Guillaume Sandoz[4,5], Tobias Huth[1,#,*], Johannes Broichhagen[2,3,#,*]

[1]Institut für Physiologie und Pathophysiologie, Friedrich-Alexander-Universität Erlangen-Nürnberg, 91054 Erlangen, Germany.

[2] Department of Chemical Biology, Max Planck Institute for Medical Research, 69120 Heidelberg, Germany.

[3] Leibniz-Forschungsinstitut für Molekulare Pharmakologie, 13125 Berlin, Germany.

[4] Université Côte d'Azur, CNRS, INSERM, iBV, 06108 Nice Cedex 2, France

[5] Laboratories of Excellence, Ion Channel Science and Therapeutics, 06108 Nice Cedex 2, France

[6] Present address: Rudolf Schoenheimer Institute of Biochemistry, Department of General Biochemistry, Medical Faculty, Leipzig University, Johannisallee 30, 04103 Leipzig, Germany.

[#]share senior authorship

*Correspondence should be addressed to:
broichhagen@fmp-berlin.de or tobias.huth@fau.de

ORCIDs:

Florian Stockinger	0009-0002-9101-7491
Pascal Poc	0000-0001-8137-4853
Alexander Möhwald	0000-0002-8540-3591
Sandra Karch	0000-0002-5101-3181
Stephanie Häfner	0000-0003-0501-2193
Christian Alzheimer	0000-0003-3910-5072
Guillaume Sandoz	0000-0003-1251-0852
Tobias Huth:	0000-0002-6414-8923
Johannes Broichhagen:	0000-0003-3084-6595

Table of Contents

Multi-color, cell-impermeable and high affinity BACE1 inhibitor probes enable superior endogenous staining and imaging of single molecules *i*

1. Chemistry **iv**

1.2.1. Dimethyl 5-((3-chloropropyl)sulfonamido)isophthalate (2) *v*

1.2.2. Dimethyl 5-((3-chloro-N-methylpropyl)sulfonamido)isophthalate (3) *v*

1.2.3. Dimethyl 5-((3-azido-N-methylpropyl)sulfonamido)isophthalate (4) *vi*

1.2.4. 3-((3-Azido-N-methylpropyl)sulfonamido)-5-(methoxycarbonyl)benzoic acid (5) *vi*

1.2.5. Methyl (R)-3-((3-azido-N-methylpropyl)sulfonamido)-5-((1-phenylethyl)-carbamoyl)benzoate (6) *vi*

1.2.6. (R)-3-((3-Azido-N-methylpropyl)sulfonamido)-5-((1-phenylethyl)carbamoyl)-benzoic acid (7) *vii*

1.2.7. (2R,3S)-3-Amino-1-(cyclopropylamino)-4-phenylbutan-2-ol (11) *vii*

1.2.8. 5-((3-Azido-N-methylpropyl)sulfonamido)-N1-((2S,3R)-4-(cyclopropylamino)-3-hydroxy-1-phenylbutan-2-yl)-N3-((R)-1-phenylethyl)isophthalamide (8) *viii*

1.2.9. 5-((3-Amino-N-methylpropyl)sulfonamido)-N1-((2S,3R)-4-(cyclopropylamino)-3-hydroxy-1-phenylbutan-2-yl)-N3-((R)-1-phenylethyl)isophthalamide (9) *viii*

1.2.10. 2-(6-Amino-3-iminio-4,5-disulfo-3H-xanthen-9-yl)-4-((3-(N-(3-(((2S,3R)-4-(cyclopropylamino)-3-hydroxy-1-phenylbutan-2-yl)carbamoyl)-5-(((R)-1-phenylethyl)carbamoyl)phenyl)-N-methylsulfamoyl)propyl)carbamoyl)benzoate (Alexa488-C3) *ix*

1.2.11. 4-((3-(N-(3-(((2S,3R)-4-(Cyclopropylamino)-3-hydroxy-1-phenylbutan-2-yl)carbamoyl)-5-(((R)-1-phenylethyl)carbamoyl)phenyl)-N-methylsulfamoyl)propyl)carbamoyl)-2-(1,2,2,10,10,11-hexamethyl-4,8-bis(sulfomethyl)-3,4,8,9,10,11-hexahydro-2H-pyrano[3,2-g:5,6-g']diquinolin-1-ium-6-yl)benzoate (Alexa568-C3) *ix*

1.2.12. 3-(6-((3-(N-(3-(((2S,3R)-4-(Cyclopropylamino)-3-hydroxy-1-phenylbutan-2-yl)carbamoyl)-5-(((R)-1-phenylethyl)carbamoyl)phenyl)-N-methylsulfamoyl)propyl)amino)-6-oxohexyl)-2-((1E,3E)-5-((E)-3,3-dimethyl-5-sulfo-1-(3-sulfopropyl)indolin-2-ylidene)penta-1,3-dien-1-yl)-3-methyl-5-sulfo-1-(3-sulfopropyl)-3H-indol-1-ium (Alexa647-C3) *ix*

2. NMR spectra **xi**

2.8. 5-((3-Amino-N-methylpropyl)sulfonamido)-N1-((2S,3R)-4-(cyclopropylamino)-3-hydroxy-1-phenylbutan-2-yl)-N3-((R)-1-phenylethyl)isophthalamide (9) *xx*

3. LCMS traces **xxi**

3.1. 2-(6-Amino-3-iminio-4,5-disulfo-3H-xanthen-9-yl)-4-((3-(N-(3-(((2S,3R)-4-(cyclopropylamino)-3-hydroxy-1-phenylbutan-2-yl)carbamoyl)-5-(((R)-1-phenylethyl)carbamoyl)phenyl)-N-methylsulfamoyl)propyl)carbamoyl)benzoate (Alexa488-C3) *xxi*

3.2. 4-((3-(N-(3-(((2S,3R)-4-(Cyclopropylamino)-3-hydroxy-1-phenylbutan-2-yl)carbamoyl)-5-(((R)-1-phenylethyl)carbamoyl)phenyl)-N-methylsulfamoyl)propyl)carbamoyl)-2-(1,2,2,10,10,11-hexamethyl-4,8-bis(sulfomethyl)-

3,4,8,9,10,11-hexahydro-2H-pyrano[3,2-g:5,6-g']diquinolin-1-ium-6-yl)benzoate (Alexa568-C3) *xxi*
3.3. 3-(6-((3-(N-(3-(((2S,3R)-4-(Cyclopropylamino)-3-hydroxy-1-phenylbutan-2-yl)carbamoyl)-5-(((R)-1-phenylethyl)carbamoyl)phenyl)-N-methylsulfamoyl)propyl)amino)-6-oxohexyl)-2-((1E,3E)-5-((E)-3,3-dimethyl-5-sulfo-1-(3-sulfopropyl)indolin-2-ylidene)penta-1,3-dien-1-yl)-3-methyl-5-sulfo-1-(3-sulfopropyl)-3H-indol-1-ium (Alexa647-C3) *xxi*
4. Supplemental Figures **xxiii**

1. Chemistry

1.1. General

All chemical reagents and anhydrous solvents for synthesis were purchased from commercial suppliers (Sigma-Aldrich, Fluka, Acros, Fluorochem, TCI) and were used without further purification or distillation. If necessary, solvents were degassed either by freeze-pump-thaw or by bubbling N_2 through the vigorously stirred solution for several minutes. For 5,6-regioisomers of Alexa488 and Alexa658 dyes, the 6-regioisomers are shown. All compounds are >95% pure by HPLC analysis.

NMR spectra were recorded in deuterated solvents on a Bruker AVANCE III HD 400 equipped with a CryoProbe and calibrated to residual solvent peaks ($^1H/^{13}C$ in ppm): $CDCl_3$ (7.26/77.00), DMSO-d_6 (2.50/39.52), acetone-d_6 (2.05/29.84), MeOD-d_4 (3.31/49.00). Multiplicities are abbreviated as follows: s = singlet, d = doublet, t = triplet, q = quartet, p = pentet, br = broad, m = multiplet. Coupling constants *J* are reported in Hz. Spectra are reported based on appearance, not on theoretical multiplicities derived from structural information.

UPLC-UV/Vis for purity assessment was performed on an Agilent 1260 Infinity II LC System equipped with Agilent SB- C18 column (1.8 µm, 2.1 × 50 mm). Buffer A: 0.1% FA in H_2O Buffer B: 0.1% FA acetonitrile. The typical gradient was from 10% B for 0.5 min → gradient to 95% B over 5 min → 95% B for 0.5 min → gradient to 99% B over 1 min with 0.8 mL/min flow. Chromatograms were imported into Graphpad Prism10 and plotted.

High resolution mass spectrometry was performed using a Bruker maXis II ETD hyphenated with a Shimadzu Nexera system. The instruments were controlled via Brukers otofControl 4.1 and Hystar 4.1 SR2 (4.1.31.1) software. The acquisition rate was set to 3 Hz and the following source parameters were used for positive mode electrospray ionization: End plate offset = 500 V; capillary voltage = 3800 V; nebulizer gas pressure = 45 psi; dry gas flow = 10 L/min; dry temperature = 250 °C. Transfer, quadrupole and collision cell settings are mass range dependent and were fine-adjusted with consideration of the respective analyte's molecular weight. For internal calibration sodium format clusters were used. Samples were desalted *via* fast liquid chromatography. A Supelco Titan™ C18 UHPLC Column, 1.9 µm, 80 Å pore size, 20 × 2.1 mm and a 2 min gradient from 10 to 98% aqueous MeCN with 0.1% FA (H_2O: Carl Roth GmbH + Co. KG ROTISOLV® Ultra LC-MS; MeCN: Merck KGaA LiChrosolv® Acetonitrile hypergrade for LC-MS; FA - Merck KGaA LiChropur® Formic acid 98%- 100% for LC-MS) was used for separation. Sample dilution in 10% aqueous ACN (hyper grade) and injection volumes were chosen dependent of the analyte's ionization efficiency. Hence, on-column loadings resulted between 0.25–5.0 ng. Automated internal re-calibration and data analysis of the recorded spectra were performed with Bruker's DataAnalysis 4.4 SR1 software.

Preparative RP-HPLC was performed on a Waters e2695 system equipped with a 2998 PDA detector for product collection (at 220, 490, 550 or 650 nm) on either a semi-preparative Supelco Ascentis® C18 HPLC Column (5 µm, 250 × 21.2 mm) or on an analytical Supelco Ascentis® C18 HPLC Column (3 µm, 150 × 2.1 mm). Buffer A: 0.1% TFA in H_2O Buffer B: MeCN. The typical gradient for semi-preparative was from 10% B for 5 min → gradient to 90% B over 45 min → 90% B for 5 min → gradient to 99% B over 5 min with 8 mL/min flow. The typical gradient for analytical was from 10% B for 5 min → gradient to 90% B over 30 min → 90% B for 5 min → gradient to 99% B over 5 min with 4 mL/min flow.

Flash column chromatography (FCC) was performed on a Biotage Isolera One with pre-packed silica columns (0.040–0.063 mm, 230-400 mesh, Silicycle). Reactions and chromatography fractions were monitored by thin layer chromatography (TLC) on Merck silica gel 60 F254 glass plates. The spots were visualized under UV light at 254 nm.

1.2. Synthesis

1.2.1. Dimethyl 5-((3-chloropropyl)sulfonamido)isophthalate (2)

A round bottom flask was charged with dimethyl 5-aminoisophthalate (**1**) (5.00 g, 23.9 mmol, 1.0 equiv.), 75 mL DCM and 25 mL pyridine. 3-Chloropropane-1-sulfonyl chloride (4.23 g, 23.9 mmol, 1.0 equiv.) was added dropwise to the suspension under vigorous stirring, the reaction mixture was stirred for additional 4 h at r.t. while turning red. The mixture was quenched by addition of 200 mL aqueous HCl (1 M) and extracted with DCM (2 x 200 mL). The combined organic layers were washed with 200 mL aqueous HCl (1 M) and brine, filtered over $MgSO_4$ and dried to obtain 6.94 g (20.6 mmol) of the desired product as a red powder in 86% yield.

^{1}H NMR (400 MHz, acetone-d_6): δ [ppm] = 8.35–8.30 (m, 1H), 8.22 (d, J = 1.5 Hz, 2H), 3.93 (s, 6H), 3.74 (t, J = 6.5 Hz, 2H), 3.51–3.30 (m, 2H), 2.36–2.22 (m, 2H).

^{13}C NMR (101 MHz, acetone-d_6): δ [ppm] = 166.0, 140.1, 132.8, 126.2, 125.0, 52.9, 49.7, 43.5, 27.8.

HRMS (ESI): calc. for $C_{13}H_{17}ClNO_6S$ $[M+H]^+$: 350.0460 and 352.0430, found: 350.0460 and 352.0431.

1.2.2. Dimethyl 5-((3-chloro-*N*-methylpropyl)sulfonamido)isophthalate (3)

A flame-dried round bottom Schlenk flask was charged with 6.44 g (18.4 mmol, 1.0 equiv.) of **2** and dissolved in 100 mL DMF under a nitrogen atmosphere and cooled to 0 °C. MeI (2.39 mL, 5.44 g, 38.4 mmol, 2.1 equiv.) was added dropwise under vigorous stirring, before 920 mg (23.0 mmol, 1.25 equiv.) of NaH (60% in mineral oil) was added portionwise. The reaction mixture turned dark purple and was allowed to warm to r.t. under stirring over 3 h. 200 mL EtOAc were added and was washed with dH_2O (2 x 250 mL) and brine, filtered over $MgSO_4$ and dried to obtain 5.56 g (15.3 mmol) of the desired product as a yellow oil that solidified upon standing in 83% yield.

^{1}H NMR (400 MHz, $CDCl_3$): δ [ppm] = 8.58 (t, J = 1.5 Hz, 1H), 8.22 (d, J = 1.5 Hz, 2H), 3.95 (s, 6H), 3.64 (t, J = 6.1 Hz, 2H), 3.40 (s, 3H), 3.26–3.13 (m, 2H), 2.33–2.24 (m, 2H).

^{13}C NMR (101 MHz, $CDCl_3$): δ [ppm] = 165.3, 141.9, 131.9, 131.0, 129.2, 52.6, 47.0, 42.7, 38.2, 26.3.

HRMS (ESI): calc. for $C_{14}H_{19}ClNO_6S$ $[M+H]^+$: 364.0616 and 366.0587, found: 364.0613 and 366.0585.

1.2.3. Dimethyl 5-((3-azido-*N*-methylpropyl)sulfonamido)isophthalate (4)

A flame-dried round bottom Schlenk flask was charged with 356 mg (0.99 mmol, 1.0 equiv.) of **3** and dissolved in 30 mL DMF under a nitrogen atmosphere. NaN_3 (74.0 mg, 1.14 mmol, 1.15 equiv.) was added and the reaction mixture was heated to 80 °C under stirring for 3 h, before dH_2O (500 mL) was added and desired product was sedimented by centrifugation (4,000 rpm for 60 min), the supernatant was collected and the residue was dried to obtain the desired product as a yellow oil that solidifies upon standing. The aqueous layer was re-extracted with 600 mL of EtOAc, which was dried over $MgSO_4$ before all volatiles were removed and dH_2O (200 mL) was added and more product was sedimented by centrifugation (4,000 rpm for 60 min) to obtain a total of 258 mg (0.70 mmol) of the desired product as an orange solid in 70% yield.

^{1}H NMR (400 MHz, $CDCl_3$): δ [ppm] = 8.60 (t, J = 1.5 Hz, 1H), 8.22 (d, J = 1.5 Hz, 2H), 3.95 (s, 6H), 3.47 (t, J = 6.4 Hz, 2H), 3.19–2.97 (m, 2H), 2.10–2.03 (m, 2H).

^{13}C NMR (101 MHz, $CDCl_3$): δ [ppm] = 165.3, 141.9, 131.9, 131.0, 129.2, 52.7, 49.5, 46.7, 38.2, 23.1.

HRMS (ESI): calc. for $C_{14}H_{19}N_4O_6S$ $[M+H]^+$: 371.1020, found: 371.1020.

1.2.4. 3-((3-Azido-*N*-methylpropyl)sulfonamido)-5-(methoxycarbonyl)benzoic acid (5)

A round bottom flask was charged with 173 mg (467 µmol, 1.0 equiv.) of **4** and dissolved in 5 mL THF, 5 mL MeOH and 240 µL of aqueous NaOH (2 M). The reaction mixture was stirred o.n. at r.t. before all volatiles were removed *in vacuo* and to obtain 159 mg (420 µmol, Na-salt) of the desired product sufficiently pure as a white foam in 90% yield.

^{1}H NMR (400 MHz, MeOD-d_4): δ [ppm] = 8.53 (t, J = 1.5 Hz, 1H), 8.20 (dd, J = 2.3, 1.5 Hz, 1H), 8.10 (dd, J = 2.3, 1.6 Hz, 1H), 3.93 (s, 3H), 3.48–3.42 (m, 2H), 3.39 (s, 3H), 3.25–3.17 (m, 2H), 2.11–1.88 (m, 2H).

^{13}C NMR (101 MHz, MeOD-d_4): δ [ppm] = 172.8, 167.7, 142.9, 141.3, 132.0, 131.8, 130.4, 129.9, 52.8, 50.7, 47.5, 38.6, 24.3.

HRMS (ESI): calc. for $C_{13}H_{17}N_4O_6S$ $[M+H]^+$: 357.0863, found: 357.0862.

1.2.5. Methyl (*R*)-3-((3-azido-*N*-methylpropyl)sulfonamido)-5-((1-phenylethyl)-carbamoyl)benzoate (6)

A round bottom flask was charged with 159 mg (420 µmol, 1.0 equiv.) of **5** (Na-salt), 61 mg (40 µL, 504 µmol, 1.2 equiv.) of (*R*)-1-phenylethan-1-amine and 223 mg (504 µmol, 1.2 equiv.) of BOP dissolved in 5 mL DCM and 220 µL (1.26 mmol, 3.0 equiv.) of DIPEA. The reaction mixture was stirred for 2 h at r.t. before it was filtered and directly subjected to FCC (DCM/MeOH, gradient from 100/0 → 90/10 over 15 CV) to obtain 118 mg (257 µmol) of the desired product as a clear oil in 61% yield.

^{1}H NMR (400 MHz, $CDCl_3$): δ [ppm] = 8.20 (dt, J = 3.4, 1.5 Hz, 1H), 8.09 (dd, J = 2.3, 1.4 Hz, 1H), 8.07–7.96 (m, 1H), 7.40–7.35 (m, 2H), 7.35–7.29 (m, 2H), 7.26–7.21 (m, 1H), 6.96 (d, J = 7.7 Hz, 1H), 5.28 (m, 1H), 3.86 (s, 3H), 3.42 (t, J = 6.5 Hz, 2H), 3.32 (s, 3H), 3.14–3.05 (m, 2H), 2.19–1.88 (m, 2H), 1.59 (d, J = 6.9 Hz, 3H).

^{13}C NMR (101 MHz, $CDCl_3$): δ [ppm] = 165.4, 164.3, 142.8, 141.9, 135.9, 131.4, 129.9, 129.1, 128.6, 127.4, 126.2, 125.9, 52.5, 49.6, 49.3, 46.5, 38.0, 22.9, 21.5.

HRMS (ESI): calc. for $C_{21}H_{26}N_5O_5S$ $[M+H]^+$: 460.1649, found: 460.1649.

1.2.6. (*R*)-3-((3-Azido-*N*-methylpropyl)sulfonamido)-5-((1-phenylethyl)carbamoyl)-benzoic acid (7)

A round bottom flask was charged with 118 mg (257 µmol, 1.0 equiv.) of **6** and dissolved in 2 mL THF, 2 mL MeOH and 130 µL of aqueous NaOH (2 M). The reaction mixture was stirred o.n. at r.t. before 20 µL of glacial HOAc was added and all volatiles were removed *in vacuo*. The residue was dissolved in DMF:dH_2O (9:1) and subjected to RP-HPLC (MeCN:H_2O + 0.1% TFA, gradient 10:90 → 90:10 over 60 min, flow 8 mL/min, λ = 220 nm) to obtain 83 mg (186 µmol) of the desired product after lyophilization as a white powder in 72% yield.

^{1}H NMR (400 MHz, DMSO-d_6): δ [ppm] = 9.12 (d, J = 7.8 Hz, 1H), 8.44–8.37 (m, 1H), 8.15–8.12 (m, 1H), 8.12–8.06 (m, 1H), 7.46–7.38 (m, 2H), 7.33 (t, J = 7.6 Hz, 2H), 7.23 (td, J = 7.0, 1.4 Hz, 1H), 5.20 (p, J = 7.2 Hz, 1H), 3.44 (t, J = 6.7 Hz, 2H), 3.35 (s, 3H), 3.29–3.22 (m, 2H), 1.88 (dq, J = 9.9, 6.9 Hz, 2H), 1.50 (d, J = 7.1 Hz, 3H).

^{13}C NMR (101 MHz, DMSO-d_6): δ [ppm] = 166.3, 163.9, 144.6, 141.8, 135.8, 131.9, 129.2, 129.1, 128.3, 126.7, 126.3, 126.1, 48.9, 48.7, 46.0, 37.7, 22.7, 22.0.

HRMS (ESI): calc. for $C_{20}H_{23}N_5O_5S$ $[M+H]^+$: 446.1493, found: 446.1493.

1.2.7. (2*R*,3*S*)-3-Amino-1-(cyclopropylamino)-4-phenylbutan-2-ol (11)

11 was prepared according to a literature procedure[1] with slight modifications: in a round bottom flask, 1.2 mL (17.23 mmol, 8.6 equiv.) of cyclopropyl amine were added to a solution of 526 mg (1.99 mmol, 1.0 equiv.) (2*S*,3*S*)-1,2-epoxy-3-(Boc-amino)-4-phenylbutane (**10**) in 6 mL of *i*PrOH. The white suspension was stirred at 50 °C for 16 h. The reaction mixture was evaporated to dryness to afford a white solid. The solid was dissolved in 4 mL of DCM and 1 mL neat TFA and stirred for 1 h. The solvent was evaporated, and the resulting oil was loaded on 15 g silica gel, washed with 40 mL of 5% MeOH:DCM and eluted with 30% MeOH:DCM (50 mL). The solvent was evaporated to afford a yellow oil. The resulting oil was dissolved in 15 mL of dH_2O:MeCN (9:1) and freeze-dried to obtain 670 mg of a crude product (double TFA salt: 1.0 mmol, 50%) as a yellowish oil which was used without further purification.

HRMS (ESI): calc. for $C_{13}H_{21}N_2O$ $[M+H]^+$: 221.1648, found: 221.1648.

1.2.8. 5-((3-Azido-*N*-methylpropyl)sulfonamido)-*N*¹-((2*S*,3*R*)-4-(cyclopropylamino)-3-hydroxy-1-phenylbutan-2-yl)-*N*³-((*R*)-1-phenylethyl)isophthalamide (8)

A round bottom flask was charged with 74.0 mg (164 µmol, 1.2 equiv.; calculated as double TFA salt) of **11** and 61.0 mg (137 mmol, 1.0 equiv.) of **7** dissolved in 2 mL DMF and 100 µL (74 mg, 573 mmol, 4.2 equiv.) DIPEA, to which 73.0 mg (164 µmol, 1.2 equiv.) of BOP was added in one portion and the reaction mixture was stirred at r.t. for 4 h. 100 µL HOAc and 200 µL of water were added and the mixture was subjected to RP-HPLC (MeCN:H_2O + 0.1% TFA, gradient 10:90 → 90:10 over 60 min, flow 8 mL/min, λ = 220 nm) to obtain 36.0 mg (55.6 µmol) of the desired product as a white powder after lyophilization in 41% yield.

^{1}H NMR (400 MHz, DMSO-d$_6$): *δ* [ppm] = 9.00 (d, *J* = 7.9 Hz, 1H), 8.73 (br s, 1H), 8.68–8.59 (m, 1H), 8.54 (d, *J* = 8.8 Hz, 1H), 8.27–8.11 (m, 1H), 8.09–7.97 (m, 1H), 7.93–7.83 (m, 1H), 7.43–7.37 (m, 2H), 7.37–7.30 (m, 2H), 7.29–7.18 (m, 5H), 7.17–7.09 (m, 1H), 5.91 (br s, 1H), 5.18 (p, *J* = 7.1 Hz, 1H), 4.28–4.09 (m, 1H), 3.98–3.82 (m, 1H), 3.47 (t, *J* = 6.7 Hz, 2H), 3.33 (s, 3H), 3.30–3.21 (m, 2H), 3.21–3.09 (m, 1H), 3.09–2.93 (m, 1H), 2.83 (dd, *J* = 13.8, 10.8 Hz, 1H), 2.78–2.66 (m, 1H), 2.03–1.82 (m, 2H), 1.50 (d, *J* = 7.1 Hz, 3H), 1.03–0.68 (m, 4H).

^{13}C NMR (101 MHz, DMSO-d$_6$): *δ* [ppm] = 165.7, 164.8, 145.0, 141.9, 139.3, 136.1, 135.6, 129.6, 128.7, 128.6, 128.2, 128.0, 127.2, 126.6, 126.5, 125.6, 69.1, 55.0, 51.4, 49.4, 49.1, 46.5, 38.3, 35.7, 30.4, 23.1, 22.5, 3.8, 3.5 (two distinct ^{13}C signals from cyclopropyl CH_2-groups confirmed by HSQC).

HRMS (ESI): calc. for $C_{33}H_{42}N_7O_5S$ $[M+H]^+$: 648.2963, found: 648.2958.

1.2.9. 5-((3-Amino-*N*-methylpropyl)sulfonamido)-*N*¹-((2*S*,3*R*)-4-(cyclopropylamino)-3-hydroxy-1-phenylbutan-2-yl)-*N*³-((*R*)-1-phenylethyl)isophthalamide (9)

A round bottom flask was charged with 16.0 mg (24.7 µmol, 1.0 equiv.) of **8** dissolved in 4 mL THF before 36.0 mg (136 µmol, 5.5 equiv.) of triphenylphosphine was added in one portion and the solution was stirred at r.t. for 16 h. The solvent was evaporated to afford a white solid that was redissolved in 500 µL and 500 µL aqueous saturated Na_2CO_3 to obtain a suspension that was stirred for 1 h at r.t. before all volatiles were evaporated and the white residue taken up in 500 µL DMF and 300 µL water and subjected to RP-HPLC (MeCN:H_2O + 0.1% TFA, gradient 10:90 → 90:10 over 60 min, flow 8 mL/min, λ = 220 nm) to obtain 3.7 mg (5.9 µmol) of the desired product after lyophilization as a white powder in 24% yield.

^{1}H NMR (400 MHz, DMSO-d$_6$): *δ* [ppm] = 9.01 (d, *J* = 8.0 Hz, 1H), 8.71 (br s, 1H), 8.62 (br s, 2H), 8.55 (d, *J* = 8.9 Hz, 1H), 8.19 (d, *J* = 1.6 Hz, 1H), 7.99 (t, *J* = 1.8 Hz, 1H), 7.88 (t, *J* = 1.8 Hz, 1H), 7.75 (br s, 3H), 7.43–7.37 (m, 2H), 7.33 (dd, *J* = 8.5, 6.7 Hz, 2H), 7.28–7.20 (m, 5H), 7.18–7.08 (m, 1H), 5.90 (s, 1H), 5.18 (p, *J* = 7.2 Hz, 1H), 4.34–4.08 (m, 1H), 3.88 (t, *J* = 8.8 Hz, 1H), 3.32 (s, 3H), 3.30 (m, 2H), 3.14 (dd, *J* = 14.0, 3.3 Hz, 1H), 2.95–2.87 (m, 2H), 2.83 (dd, *J* = 13.9, 10.8 Hz, 1H), 2.73 (t, *J* = 5.3 Hz, 1H), 1.97 (p, *J* = 7.7 Hz, 2H), 1.50 (d, *J* = 7.0 Hz, 3H), 1.05–0.60 (m, 4H).

^{13}**C NMR** (101 MHz, DMSO-d$_6$): δ [ppm] = 165.2, 164.4, 144.6, 141.3, 138.9, 135.7, 135.2, 129.1, 128.3, 128.1, 128.0, 127.4, 126.8, 126.1, 126.0, 125.1, 68.6, 54.6, 50.9, 48.7, 45.7, 37.9, 37.5, 35.4, 29.9, 22.0, 21.1, 3.4, 3.0. (two distinct ^{13}C signals from cyclopropyl CH$_2$-groups confirmed by HSQC)

HRMS (ESI): calc. for $C_{33}H_{45}N_5O_5S$ $[M+2H]^{2+}$: 311.6565, found: 311.6565.

1.2.10. 2-(6-Amino-3-iminio-4,5-disulfo-3*H*-xanthen-9-yl)-4-((3-(*N*-(3-(((2*S*,3*R*)-4-(cyclopropylamino)-3-hydroxy-1-phenylbutan-2-yl)carbamoyl)-5-(((*R*)-1-phenylethyl)carbamoyl)phenyl)-*N*-methylsulfamoyl)propyl)carbamoyl)benzoate (Alexa488-C3)

A round bottom flask was charged with 100 µL of **9** (200 µg/100 µL DMF, 200 µg, 322 nmol, 1.0 equiv.), 1.0 µL of DIPEA (740 µg, 95 µmol, 295 equiv.), and 20 µL of Alexa488 NHS ester (Thermo Fisher #A20000, 100 µg/10 µL DMSO, 200 µg, 317 nmol, 1.0 equiv.) were mixed at r.t. for 30 min. 250 µL of water and 250 µL of MeCN were added, and the mixture was directly subjected to RP-HPLC (MeCN:H$_2$O + 0.1% TFA, gradient 10:90 → 90:10 over 45 min, flow 4 mL/min, λ = 500 nm) to obtain 193 nmol of the desired product as an orange powder after lyophilization in 62% yield. Purity: 97%.

HRMS (ESI): calc. for $C_{54}H_{56}N_7O_{15}S_3$ $[M+H]^+$: 1138.2991, found: 1138.2993.

1.2.11. 4-((3-(*N*-(3-(((2*S*,3*R*)-4-(Cyclopropylamino)-3-hydroxy-1-phenylbutan-2-yl)carbamoyl)-5-(((*R*)-1-phenylethyl)carbamoyl)phenyl)-*N*-methylsulfamoyl)propyl)carbamoyl)-2-(1,2,2,10,10,11-hexamethyl-4,8-bis(sulfomethyl)-3,4,8,9,10,11-hexahydro-2*H*-pyrano[3,2-*g*:5,6-*g'*]diquinolin-1-ium-6-yl)benzoate (Alexa568-C3)

A round bottom flask was charged with 100 µL of **9** (200 µg/100 µL DMF, 200 µg, 322 nmol, 1.27 equiv.), 1.0 µL of DIPEA (740 µg, 95 µmol, 375 equiv.), and 20 µL of Alexa568 NHS ester (Thermo Fisher #A20003, 100 µg/10 µL DMSO, 200 µg, 253 nmol, 1.0 equiv.) were mixed at r.t. for 30 min. 250 µL of water and 250 µL of MeCN were added, and the mixture was directly subjected to RP-HPLC (MeCN:H$_2$O + 0.1% TFA, gradient 10:90 → 90:10 over 45 min, flow 4 mL/min, λ = 570 nm) to obtain 169 nmol of the desired product as a purple powder after lyophilization in 67% yield. Purity: 97%.

HRMS (ESI): calc. for $C_{66}H_{73}N_7O_{15}S_3$ $[M+2H]^{2+}$: 649.7158, found: 649.7154.

1.2.12. 3-(6-((3-(*N*-(3-(((2*S*,3*R*)-4-(Cyclopropylamino)-3-hydroxy-1-phenylbutan-2-yl)carbamoyl)-5-(((*R*)-1-phenylethyl)carbamoyl)phenyl)-*N*-methylsulfamoyl)propyl)amino)-6-oxohexyl)-2-((1*E*,3*E*)-5-((*E*)-3,3-dimethyl-5-sulfo-1-(3-sulfopropyl)indolin-2-ylidene)penta-1,3-dien-1-yl)-3-methyl-5-sulfo-1-(3-sulfopropyl)-3*H*-indol-1-ium (Alexa647-C3)

A round bottom flask was charged with 22 µL of **9** (550 µg/100 µL DMF, 121 µg, 195 nmol, 1.86 equiv.), 1.39 µL of DIPEA (2 µL/50 µL DMF, 41 µg, 320 nmol, 3.05 equiv.) and 100 µL of Alexa647 NHS ester (Thermo Fisher #A20006, 100 µg/100 µL DMF, 100 µg, 105 nmol, 1.0 equiv.) were mixed at r.t. for 30 min. 100 µL of water was added, and the mixture was directly subjected to RP-HPLC (MeCN:H_2O + 0.1% TFA, gradient 1:99 → 90:10 over 45 min, flow 4 mL/min, λ = 650 nm) to obtain 60 nmol of the desired product as a blue powder after lyophilization in 57% yield. Purity: 99%.

HRMS (ESI): calc. for $C_{69}H_{89}N_7O_{18}S_5$ $[M+H]^{2+}$: 731.7428, found: 731.7423.

2. NMR spectra

2.1. Dimethyl 5-((3-chloropropyl)sulfonamido)isophthalate (2)

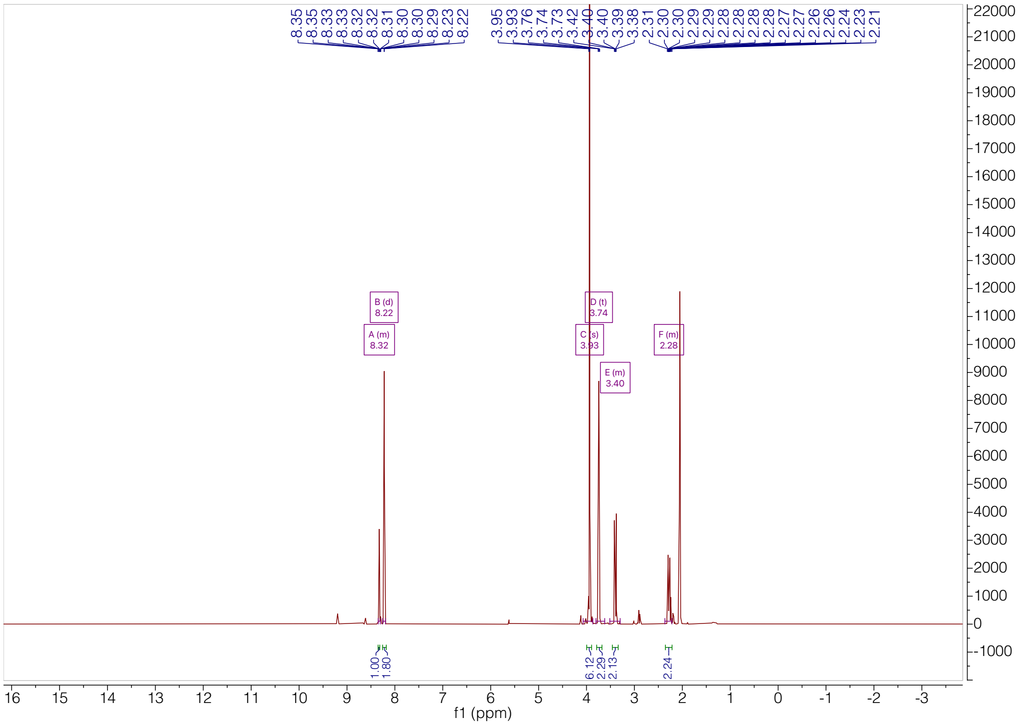

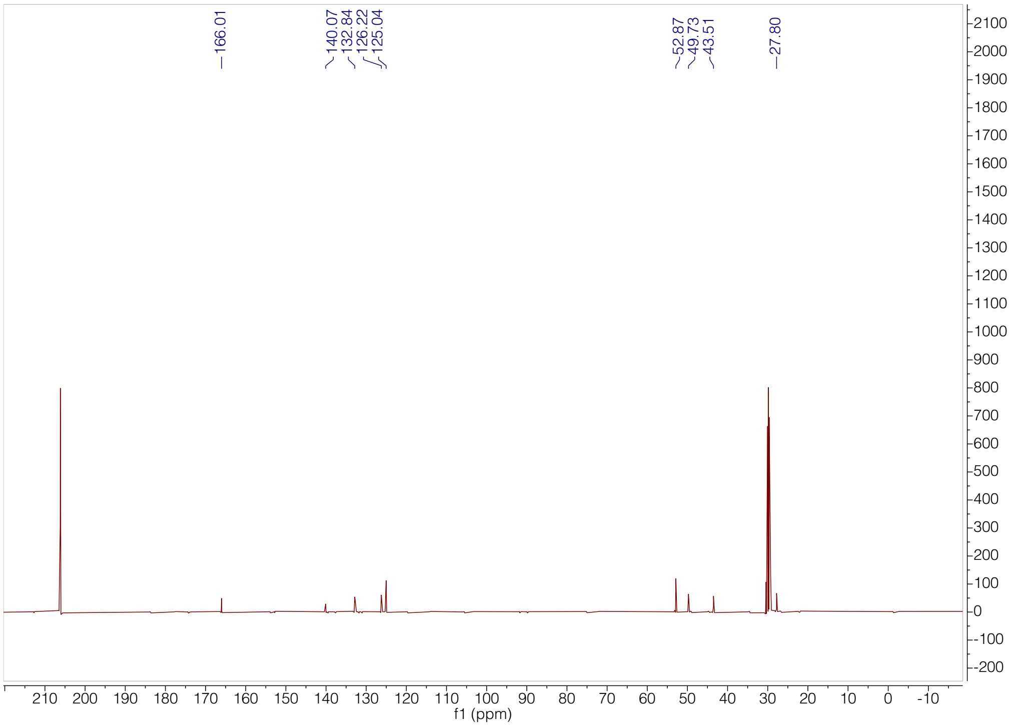

2.2. Dimethyl 5-((3-chloro-*N*-methylpropyl)sulfonamido)isophthalate (3)

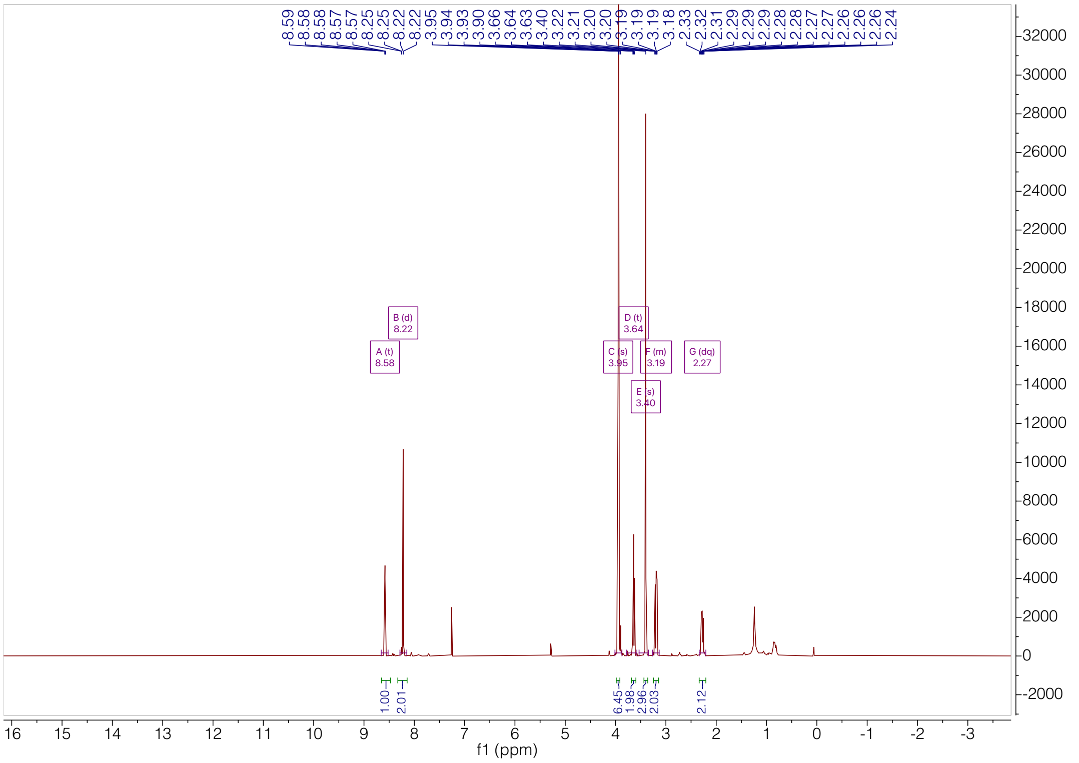

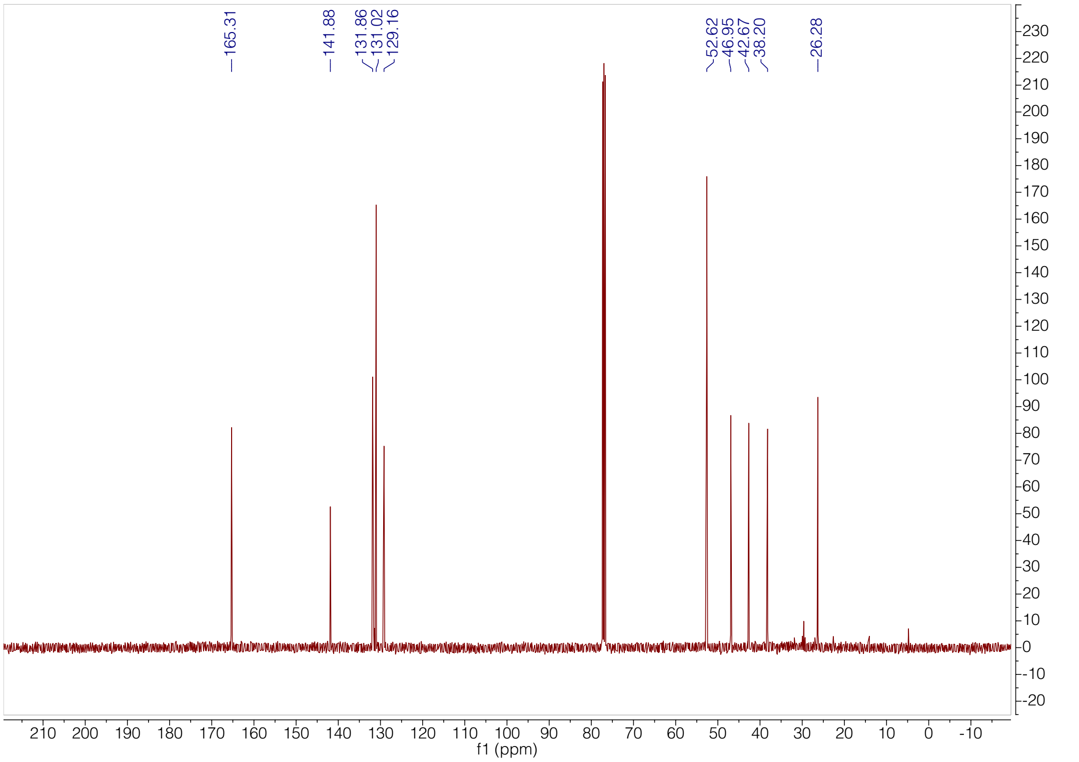

2.3. Dimethyl 5-((3-azido-*N*-methylpropyl)sulfonamido)isophthalate (4)

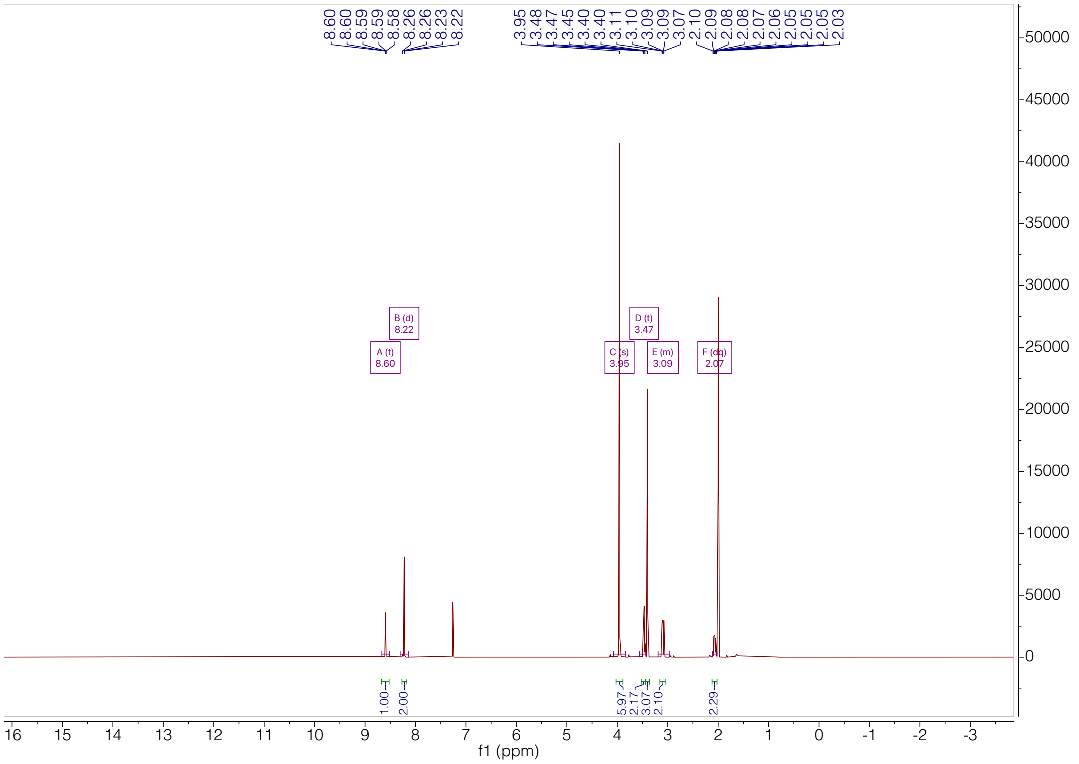

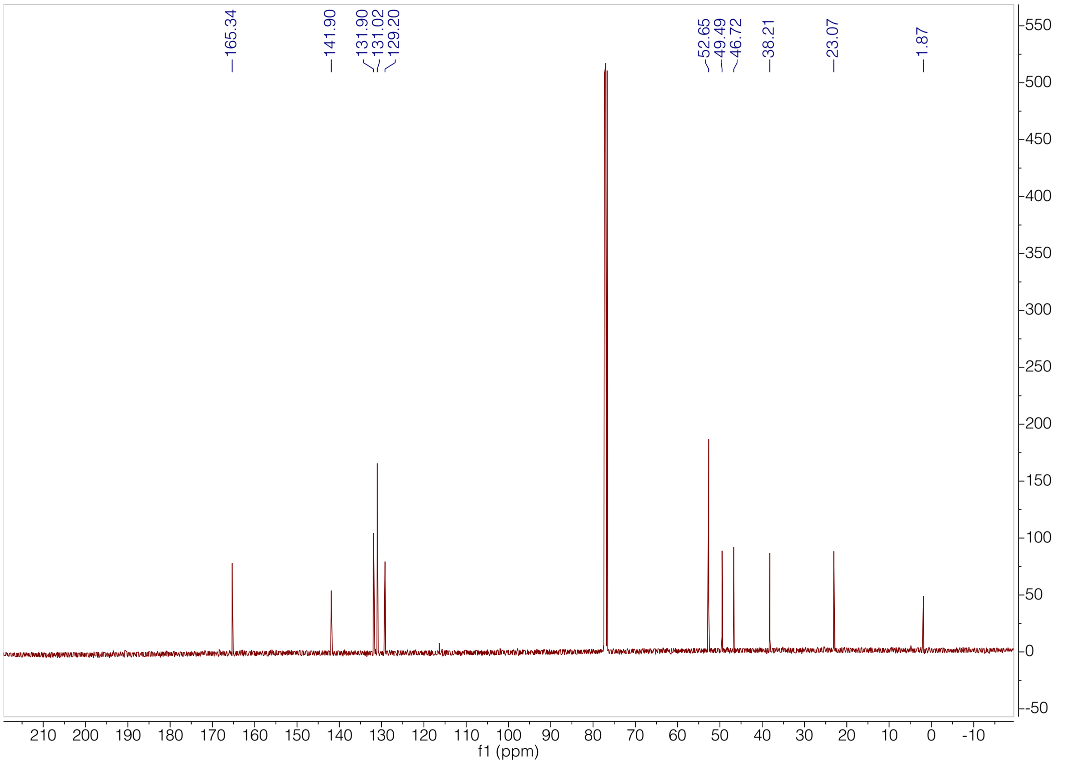

2.4. 3-((3-Azido-*N*-methylpropyl)sulfonamido)-5-(methoxycarbonyl)benzoic acid (5)

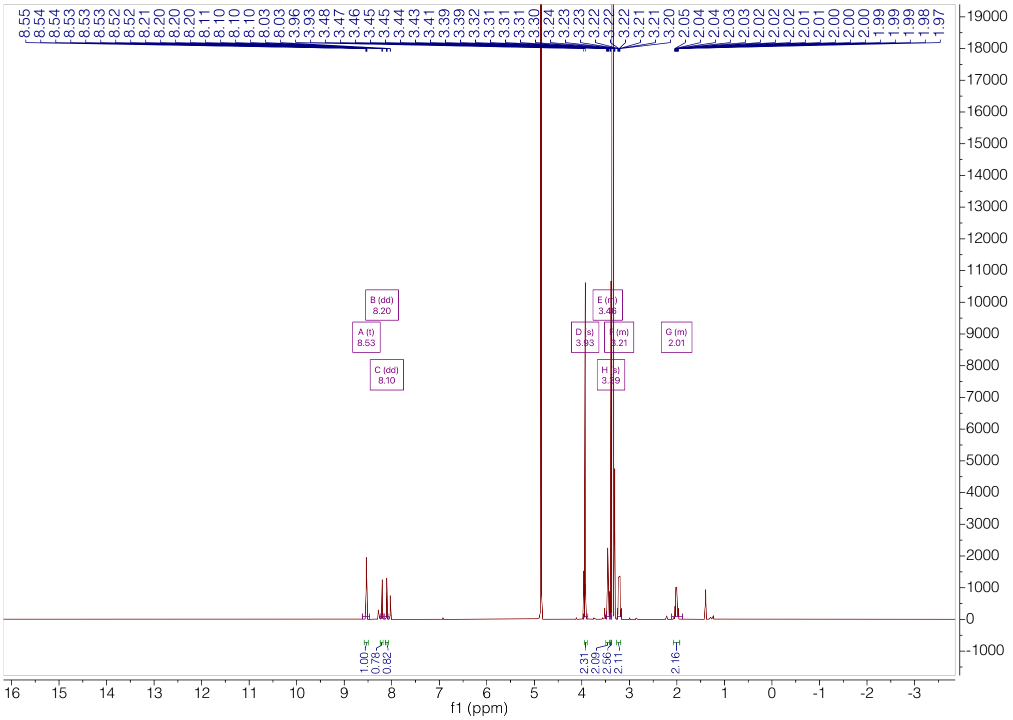

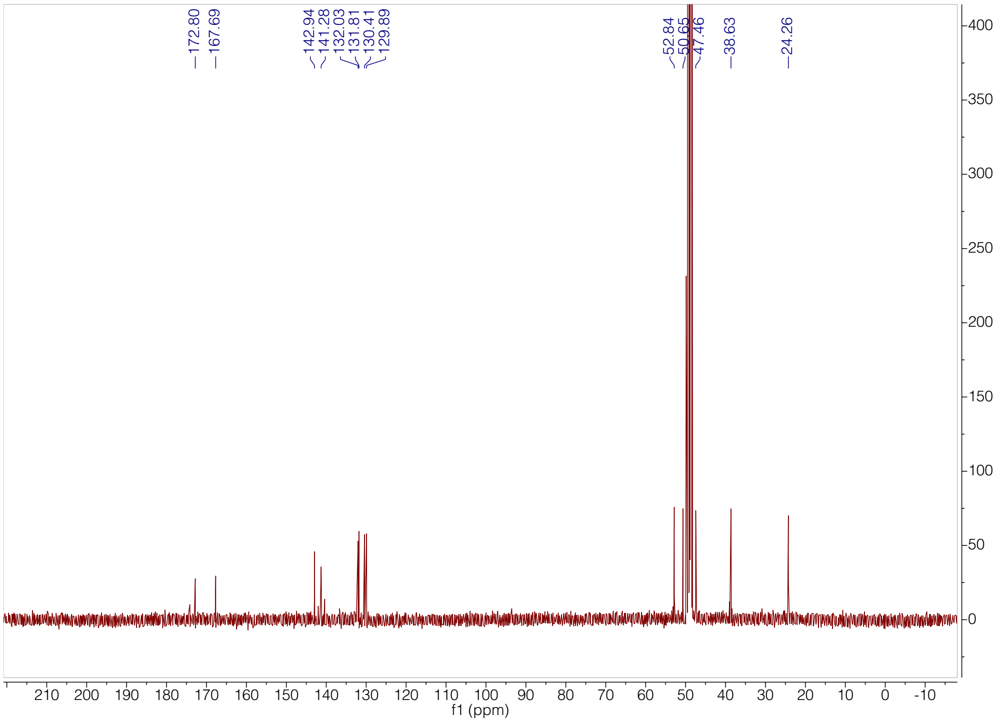

2.5. Methyl (*R*)-3-((3-azido-*N*-methylpropyl)sulfonamido)-5-((1-phenylethyl)-carbamoyl)benzoate (6)

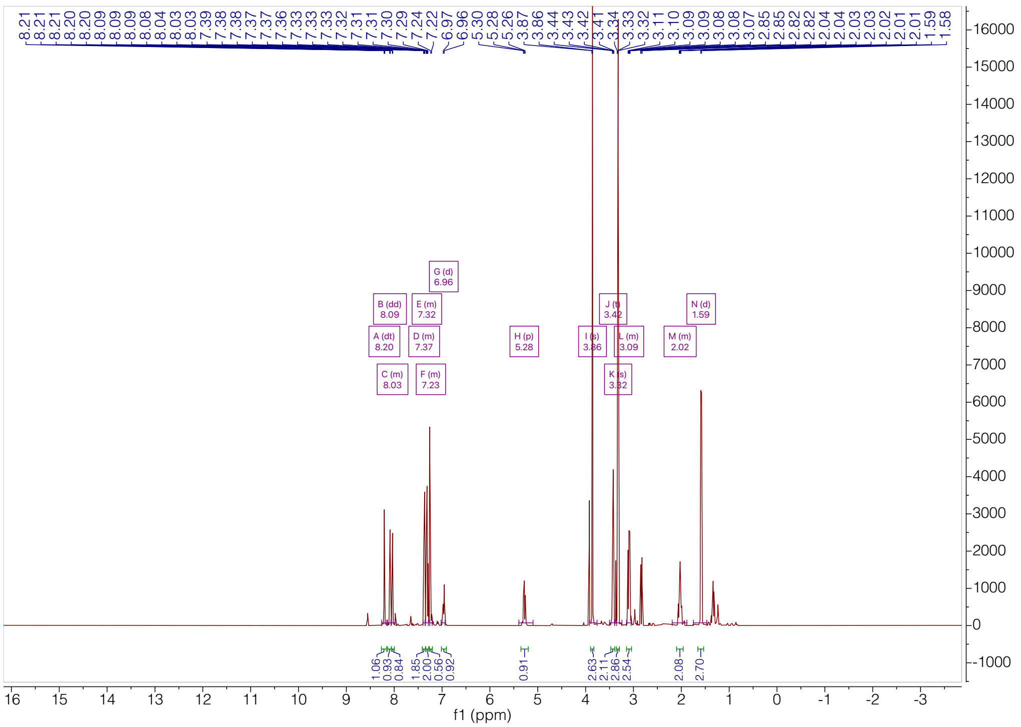

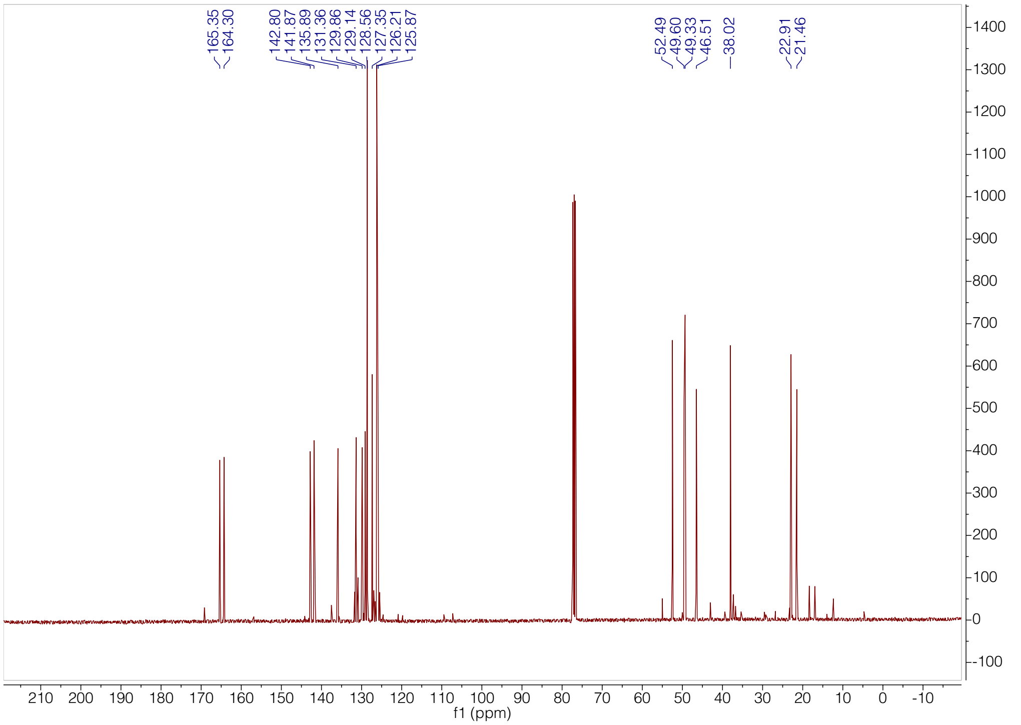

2.6. (*R*)-3-((3-Azido-*N*-methylpropyl)sulfonamido)-5-((1-phenylethyl)carbamoyl)-benzoic acid (7)

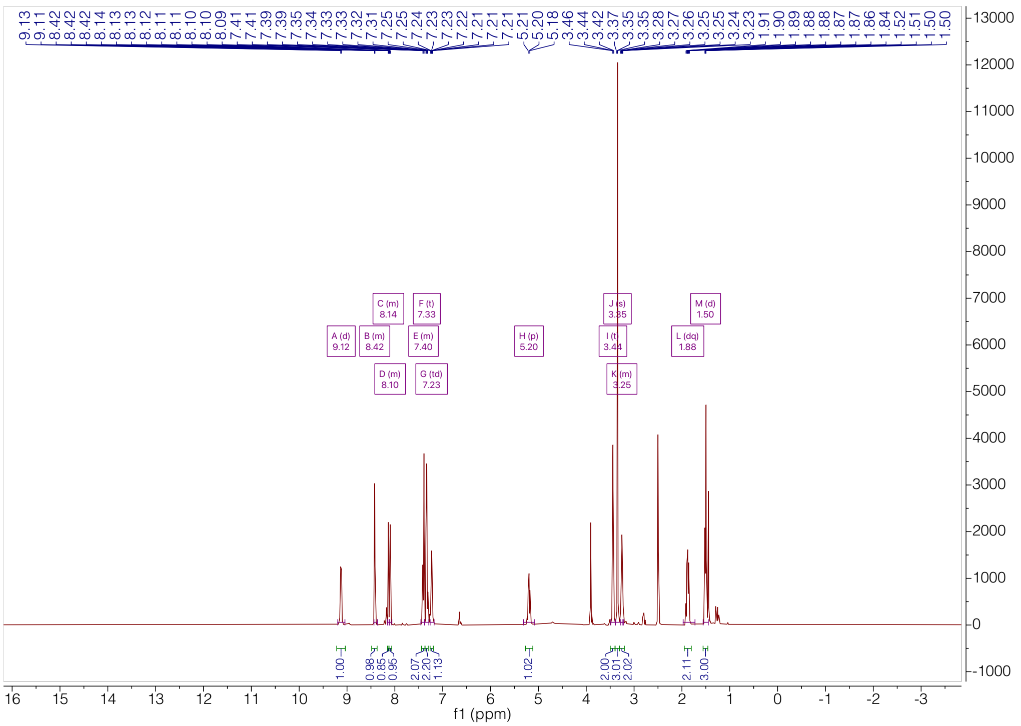

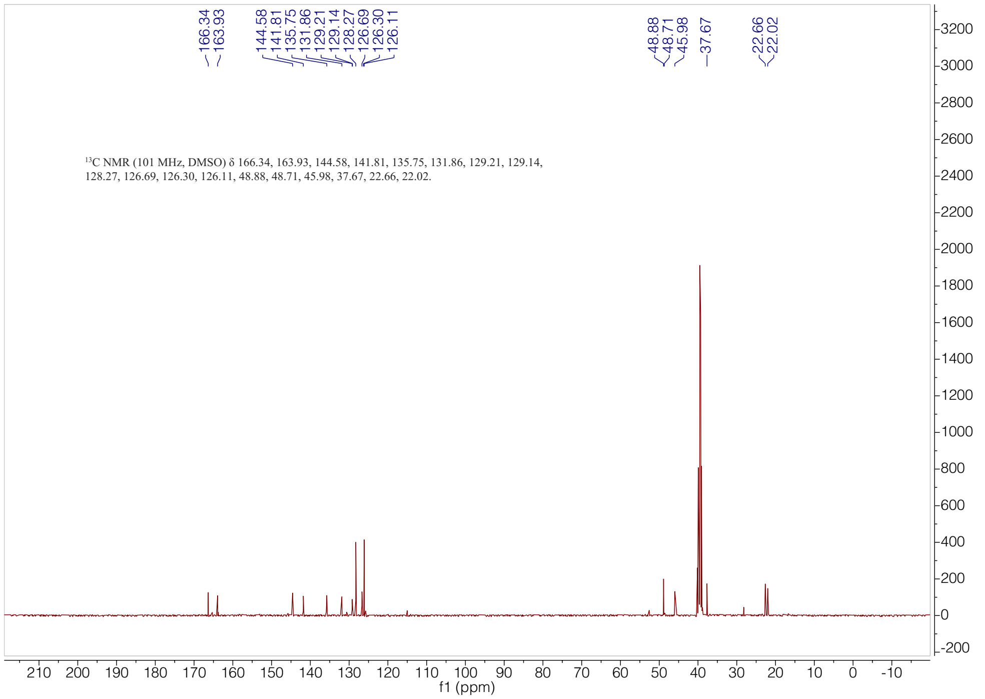

2.7. 5-((3-Azido-*N*-methylpropyl)sulfonamido)-*N*[1]-((2*S*,3*R*)-4-(cyclopropylamino)-3-hydroxy-1-phenylbutan-2-yl)-*N*[3]-((*R*)-1-phenylethyl)isophthalamide (8)

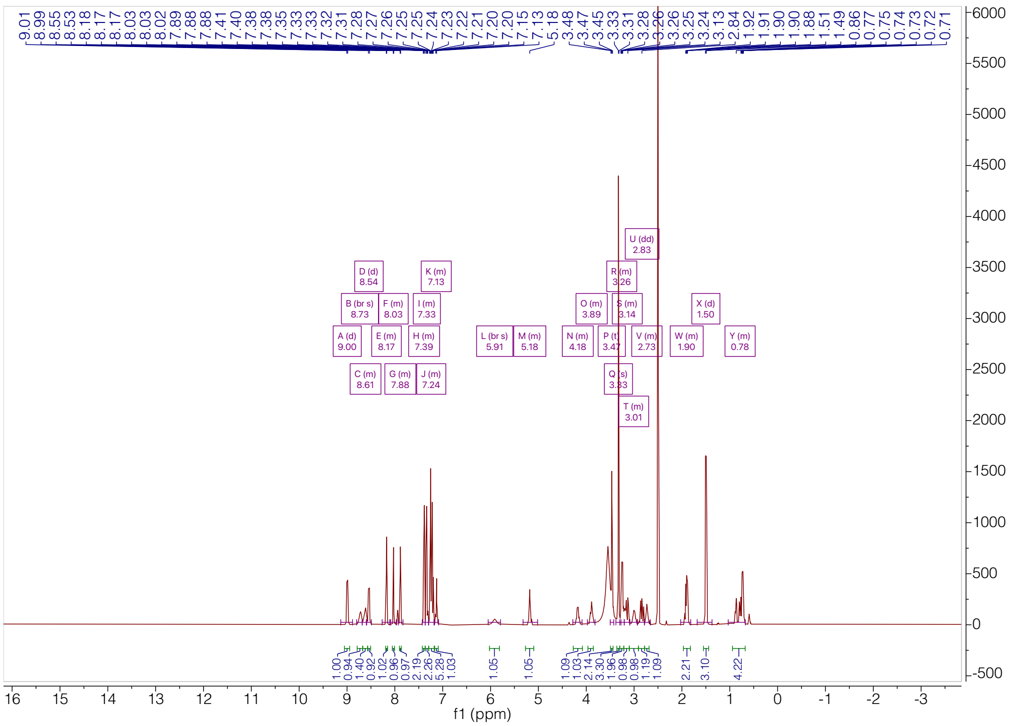

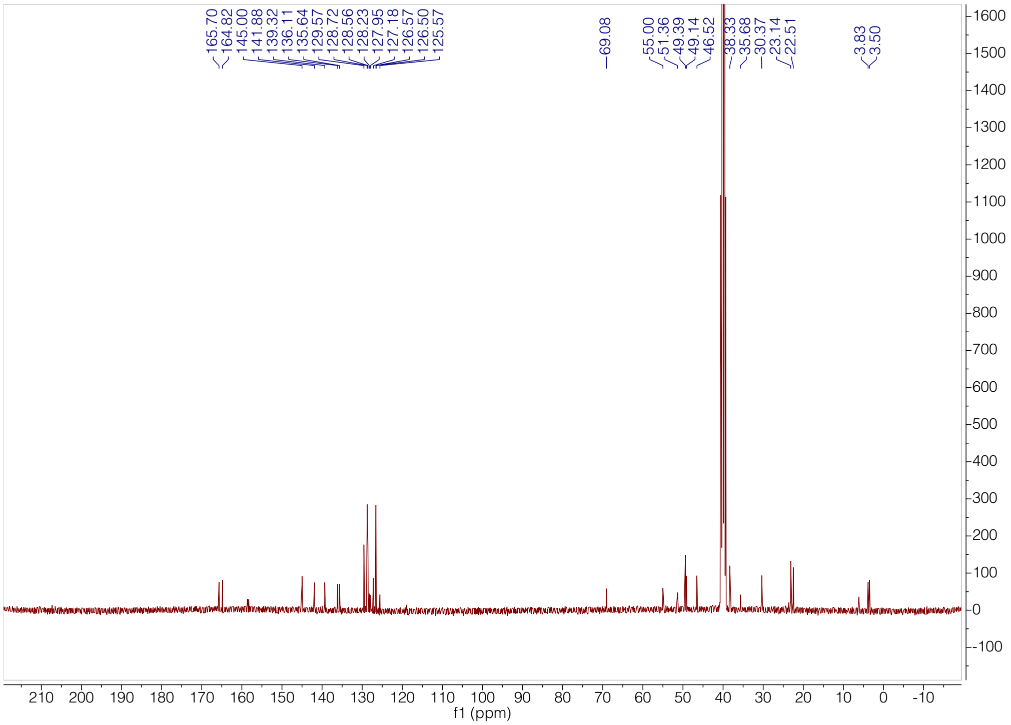

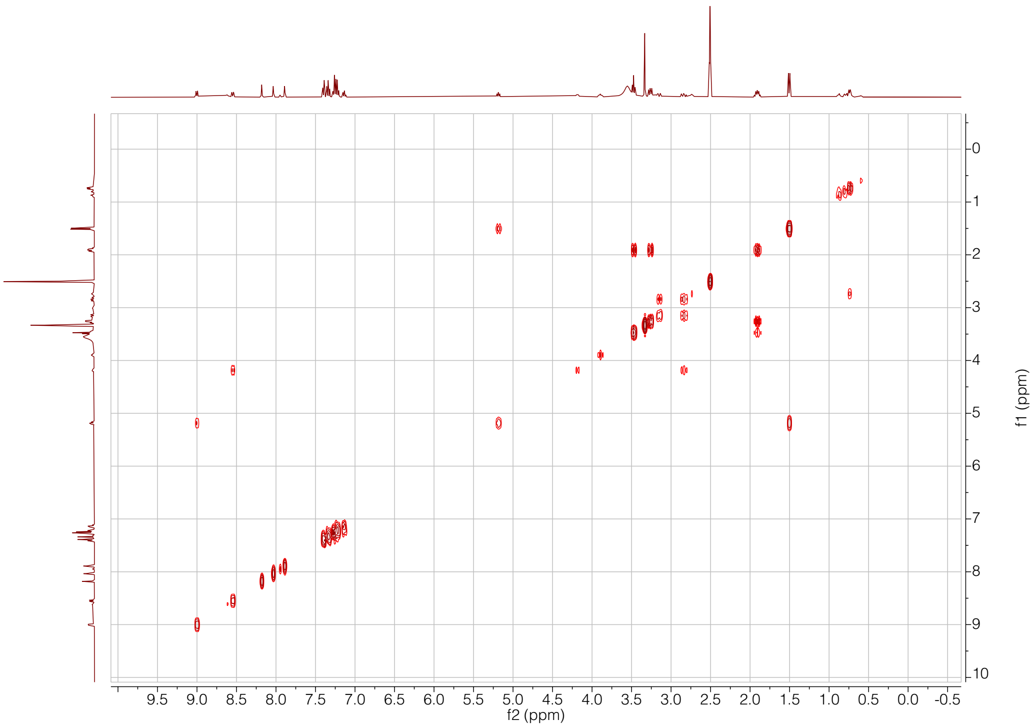

COSY

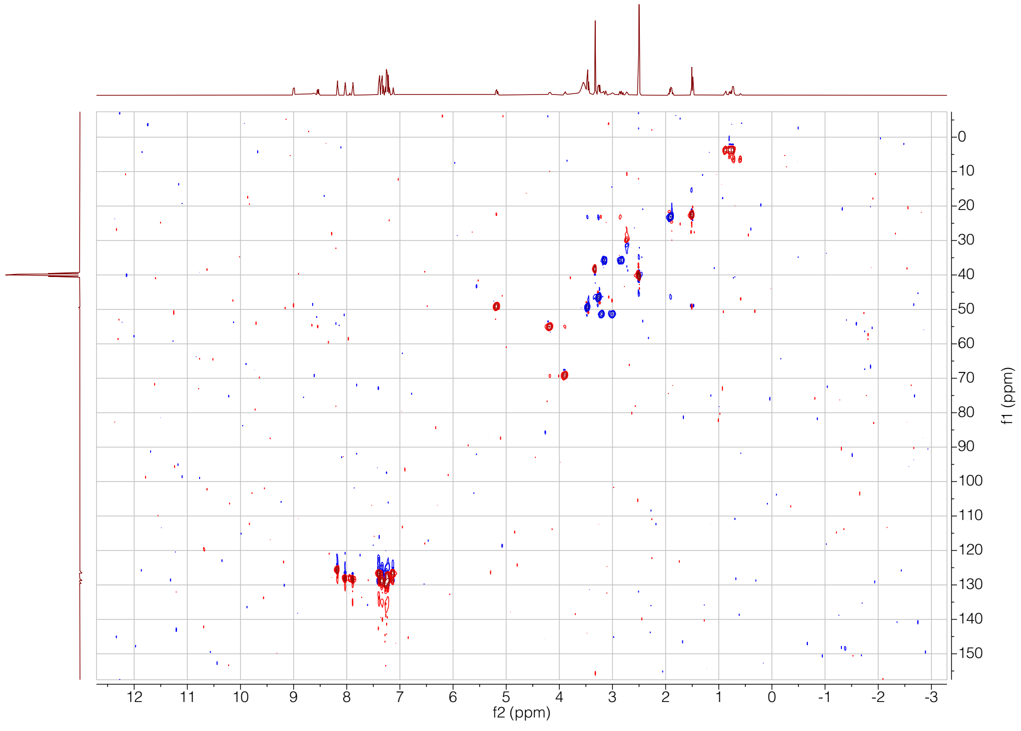

HSQC

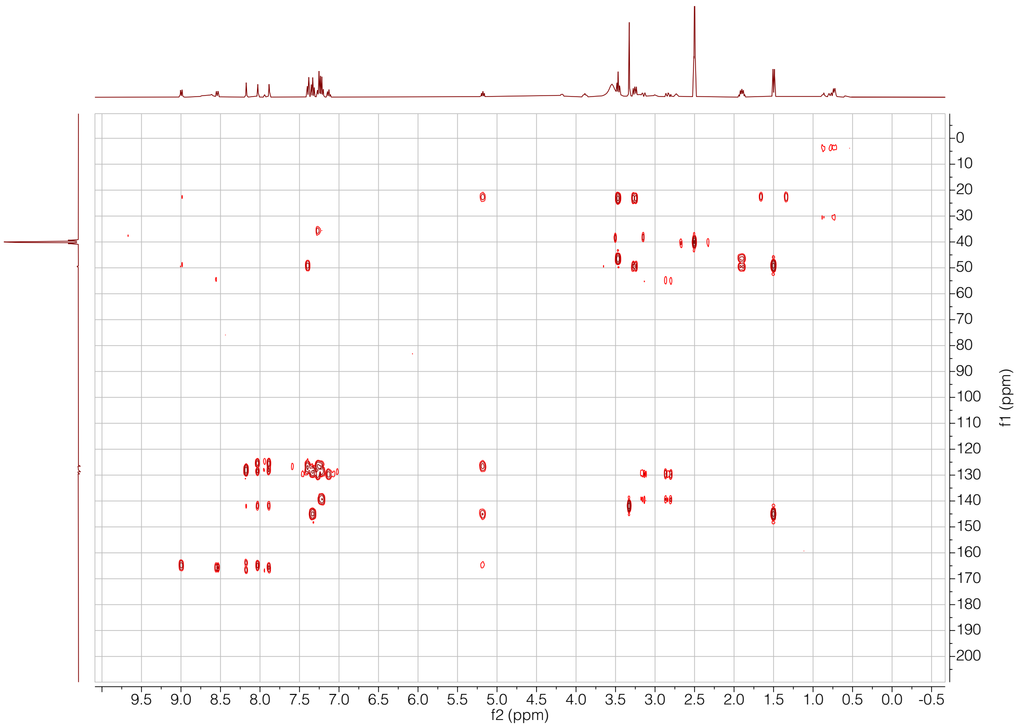

HMBC

2.8. 5-((3-Amino-*N*-methylpropyl)sulfonamido)-*N*[1]-((2*S*,3*R*)-4-(cyclopropylamino)-3-hydroxy-1-phenylbutan-2-yl)-*N*[3]-((*R*)-1-phenylethyl)isophthalamide (9)

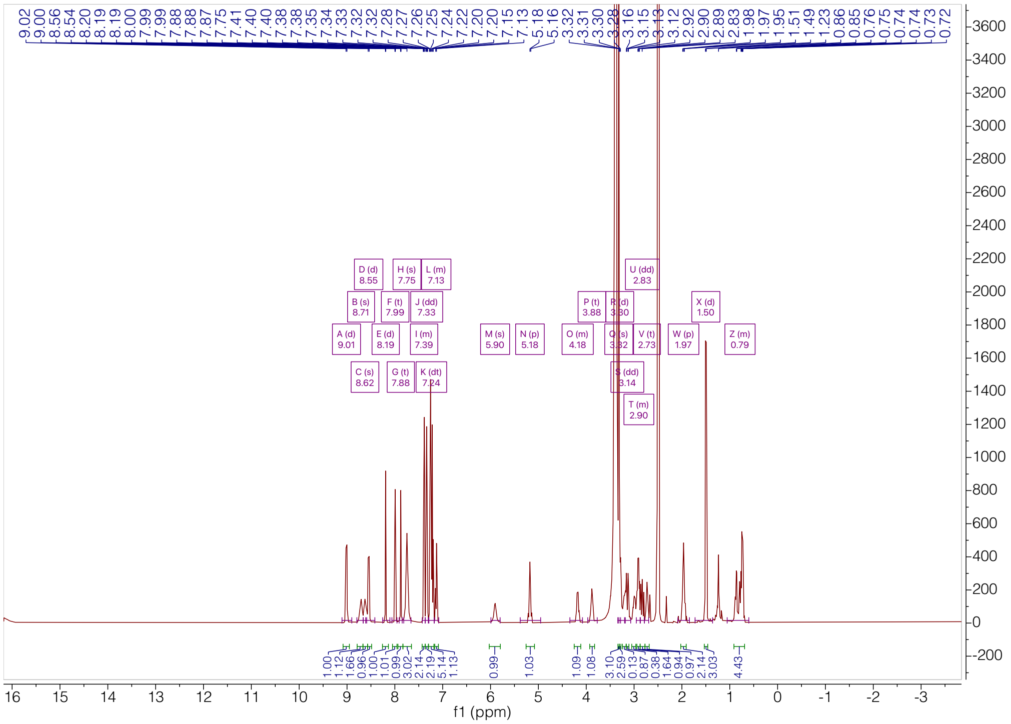

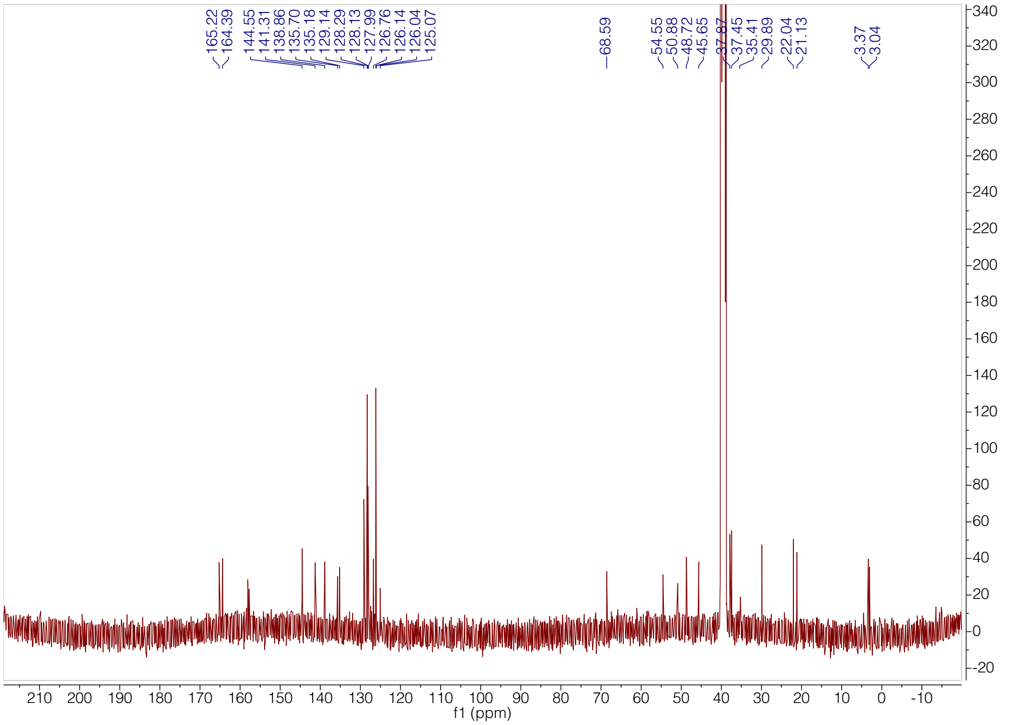

3. LCMS traces

3.1. 2-(6-Amino-3-iminio-4,5-disulfo-3*H*-xanthen-9-yl)-4-((3-(*N*-(3-(((2*S*,3*R*)-4-(cyclopropylamino)-3-hydroxy-1-phenylbutan-2-yl)carbamoyl)-5-(((*R*)-1-phenylethyl)carbamoyl)phenyl)-*N*-methylsulfamoyl)propyl)carbamoyl)benzoate (Alexa488-C3)

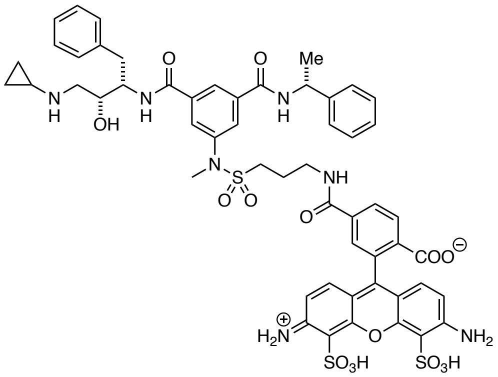

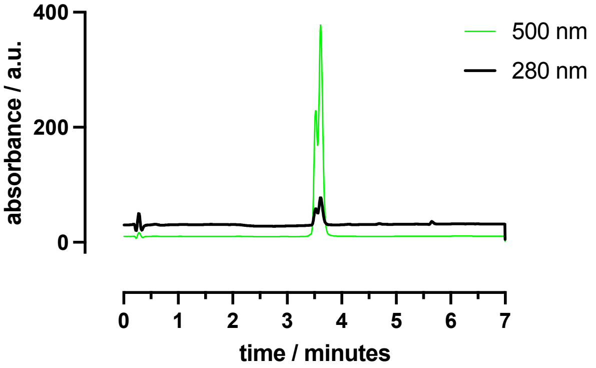

3.2. 4-((3-(*N*-(3-(((2*S*,3*R*)-4-(Cyclopropylamino)-3-hydroxy-1-phenylbutan-2-yl)carbamoyl)-5-(((*R*)-1-phenylethyl)carbamoyl)phenyl)-*N*-methylsulfamoyl)propyl)carbamoyl)-2-(1,2,2,10,10,11-hexamethyl-4,8-bis(sulfomethyl)-3,4,8,9,10,11-hexahydro-2*H*-pyrano[3,2-*g*:5,6-*g*′]diquinolin-1-ium-6-yl)benzoate (Alexa568-C3)

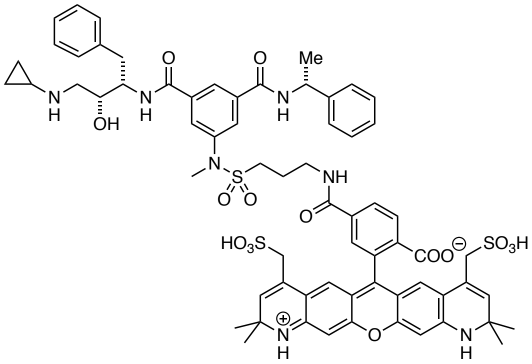

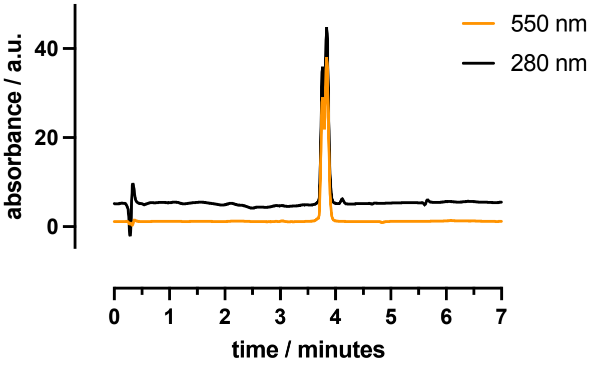

3.3. 3-(6-((3-(*N*-(3-(((2*S*,3*R*)-4-(Cyclopropylamino)-3-hydroxy-1-phenylbutan-2-yl)carbamoyl)-5-(((*R*)-1-phenylethyl)carbamoyl)phenyl)-*N*-methylsulfamoyl)propyl)amino)-6-oxohexyl)-2-((1*E*,3*E*)-5-((*E*)-3,3-dimethyl-5-sulfo-1-(3-sulfopropyl)indolin-2-ylidene)penta-1,3-dien-1-yl)-3-methyl-5-sulfo-1-(3-sulfopropyl)-3*H*-indol-1-ium (Alexa647-C3)

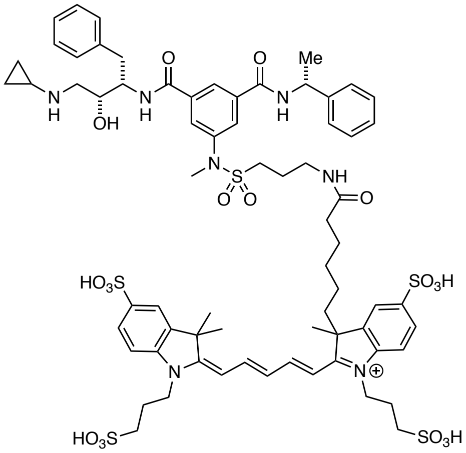

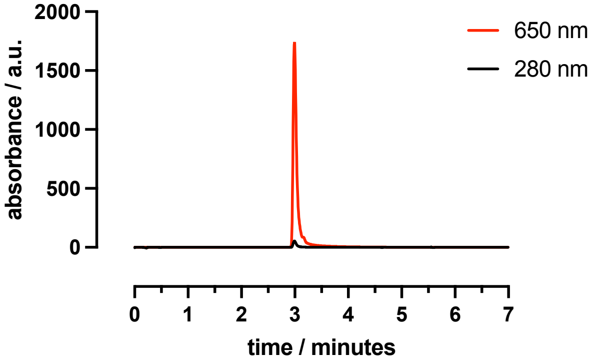

2000
1500
1000
500
0
absorbance / a.u.
650 nm
280 nm
0
1
2
3
4
5
6
7
time / minutes

4. Supplemental Figures

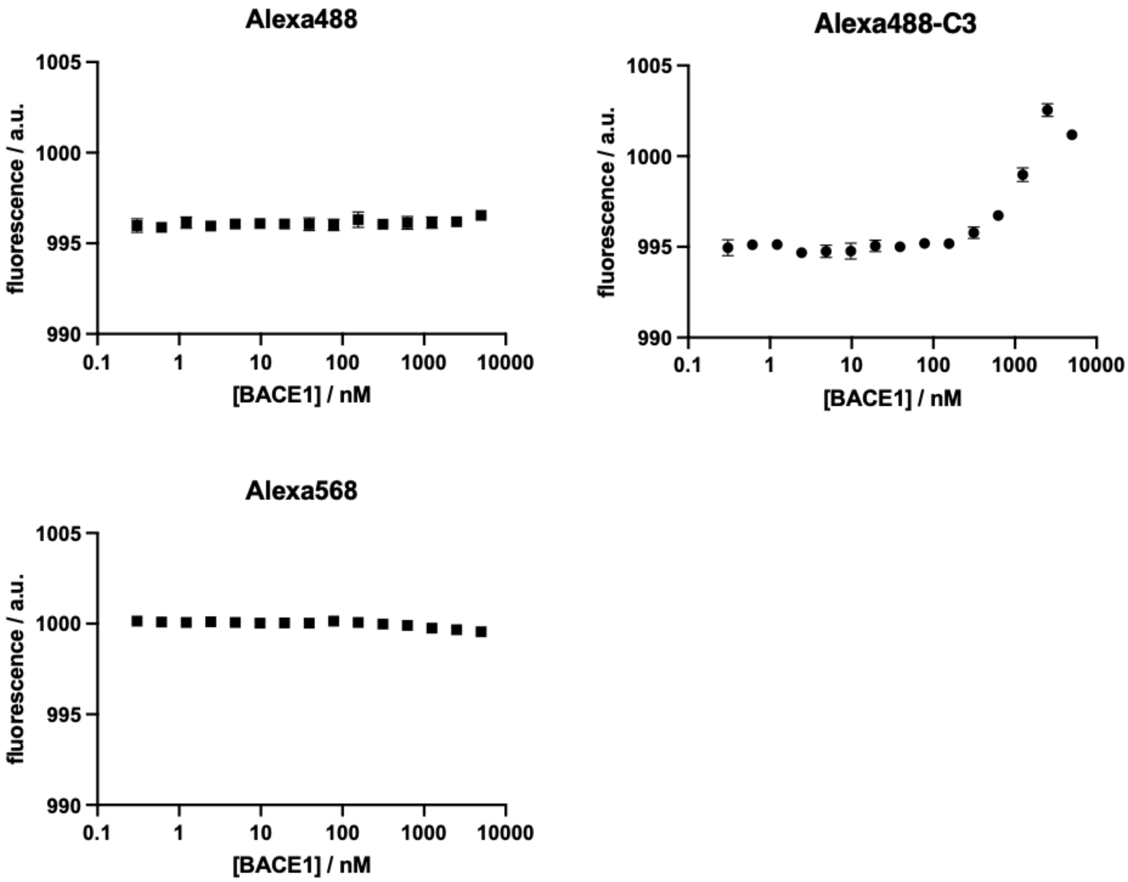

Figure S1: **Nanotemper binding assay of Alexa488, Alexa568 and Alexa488-C3.**

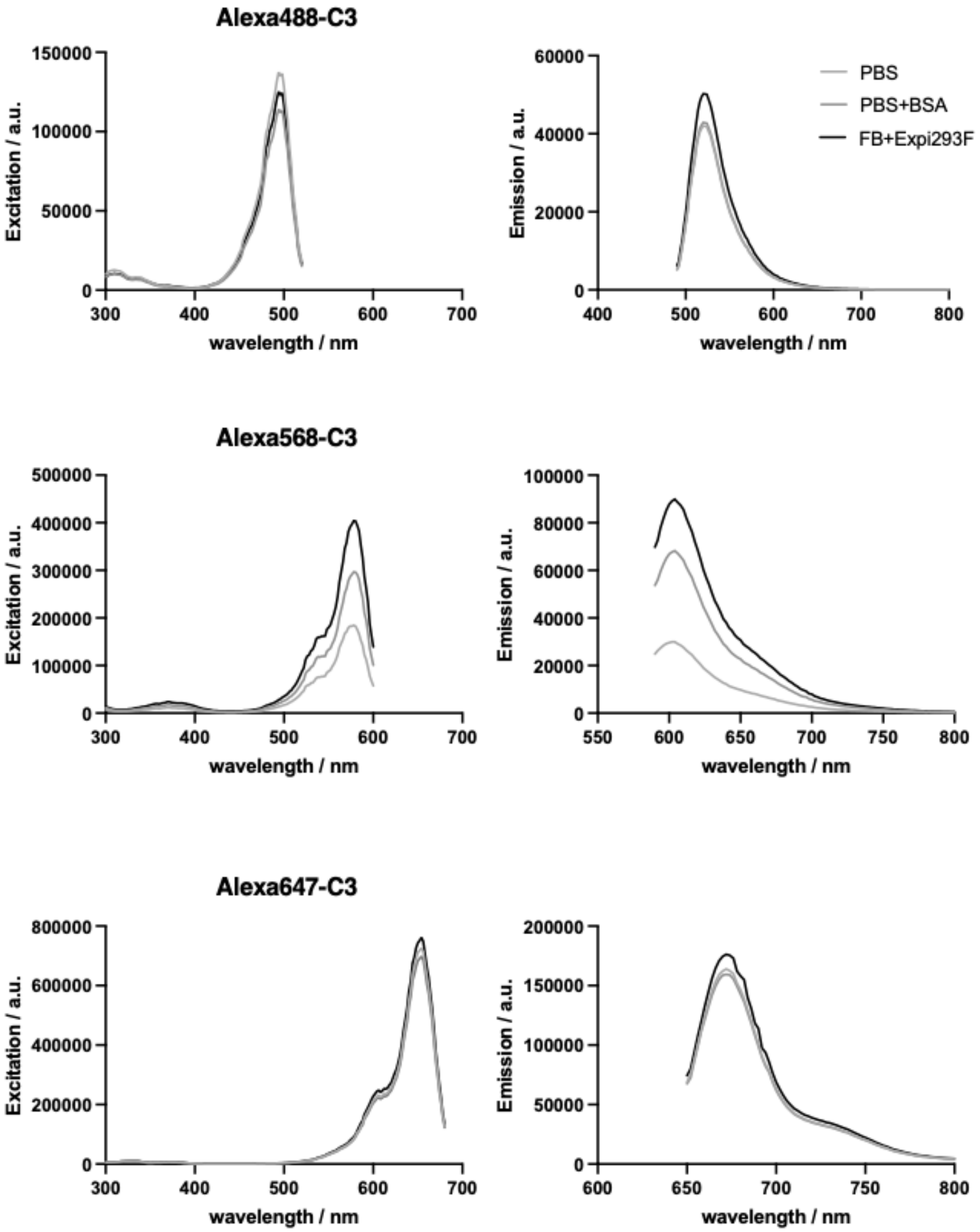

Figure S2: **Fluorescence excitation and emission profiles for Alexa-C3 compounds in PBS, PBS+BSA, and in the presence of Expi293F cells in Fluorobrite**

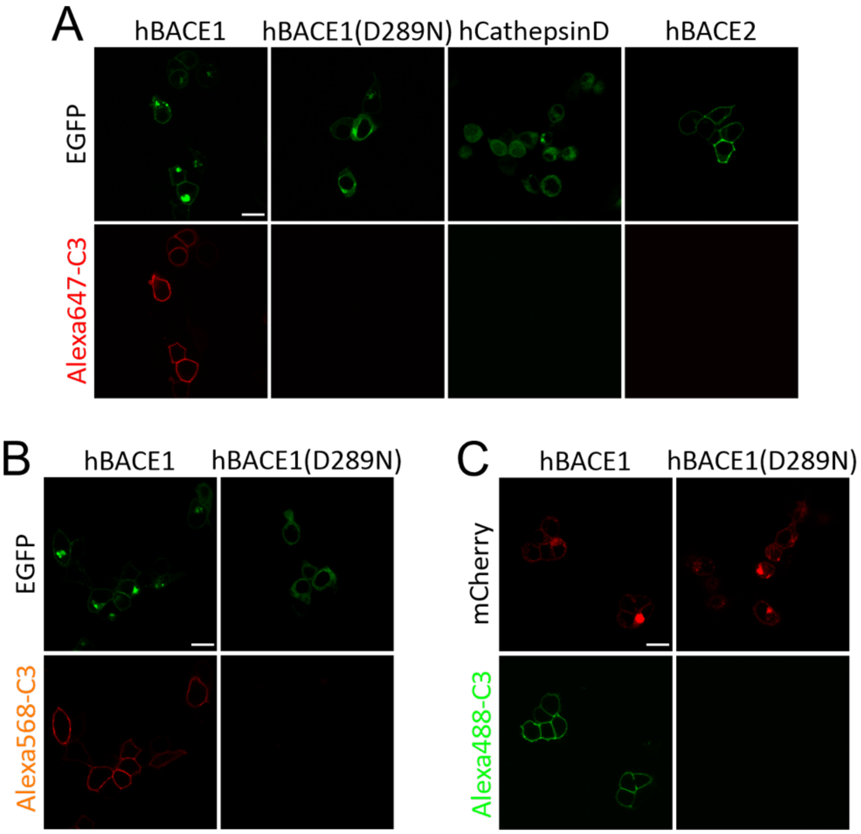

Figure S3: **Specificity of Alexa-C3 staining with an extended Field of view.** Cells were transfected with EGFP or mCherry c-terminal fusion constructs of BACE1, BACE2, BACE1 D289N, or cathepsin D constructs, respectively. 24 hours after transfection living cells were stained with (A) 100 nM Alexa647-C3, (B) 10 nM Alexa568-C3, or (C) 50 nM Alexa488-C3. Scale bars represent 20 µm. Emission was collected at 493-598 nm (for EGFP), 572-712 nm (for mCherry), 490-568 nm (for Alexa488-C3), 572-712 nm (for Alexa568-C3), and 638-755 nm (for Alexa647-C3). Samples were excited at 488 nm, 561nm and 633nm, respectively.
